# A Refined Well-Posedness Result for the Modified KdV Equation in the Fourier–Lebesgue Spaces

**DOI:** 10.1007/s10884-021-10050-0

**Published:** 2021-07-30

**Authors:** Andreia Chapouto

**Affiliations:** School of Mathematics, The University of Edinburgh, James Clerk Maxwell Building, The King’s Buildings, Peter Guthrie Tait Road, Edinburgh, EH9 3FD UK

**Keywords:** Modified Korteweg-de Vries equation, Global well-posedness, Fourier–Lebesgue spaces, 35Q53

## Abstract

We study the well-posedness of the complex-valued modified Korteweg-de Vries equation (mKdV) on the circle at low regularity. In our previous work (2021), we introduced the second renormalized mKdV equation, based on the conservation of momentum, which we proposed as the correct model to study the complex-valued mKdV outside $H^\frac{1}{2}({\mathbb {T}})$. Here, we employ the method introduced by Deng et al. (Commun Math Phys 384(1):1061–1107, 2021) to prove local well-posedness of the second renormalized mKdV equation in the Fourier–Lebesgue spaces ${\mathcal {F}}L^{s,p}({\mathbb {T}})$ for $s\ge \frac{1}{2}$ and $1\le p <\infty$. As a byproduct of this well-posedness result, we show ill-posedness of the complex-valued mKdV without the second renormalization for initial data in these Fourier–Lebesgue spaces with infinite momentum.

## Introduction

### Modified Korteweg-de Vries Equation

We consider the Cauchy problem for the complex-valued modified Korteweg-de Vries equation (mKdV) on the one-dimensional torus ${\mathbb {T}}={\mathbb {R}}/ (2\pi {\mathbb {Z}})$:

1.1 $$\begin{aligned} {\left\{ \begin{array}{ll} \partial _t u + \partial _x^3 u = \pm |u|^2 \partial _x u, \\ u\vert _{t=0} = u_0, \end{array}\right. } \quad (t,x) \in {\mathbb {R}}\times {\mathbb {T}}, \end{aligned}$$

where *u* is a complex-valued function. This equation (1.1) appears as a model for the dynamical evolution of nonlinear lattices, fluid dynamics, and plasma physics (see [8, 17, 34], for example). In the context of nonlinear fiber optics, (1.1) describes the transmission of electromagnetic waves in nematic liquid crystals, where the rescaled variables *t* and *x* represent distance and time, respectively. In the following, we choose the standard convention, referring to $x\in {\mathbb {T}}$ as the spatial variable and to $t\in {\mathbb {R}}$ as the temporal variable. Also, note that the complex-valued mKdV equation (1.1), also known as the mKdV equation of Hirota [19], is a completely integrable complex-valued generalization of the usual mKdV equation

1.2 $$\begin{aligned} \partial _t u + \partial _x^3 u = \pm u^2 \partial _x u. \end{aligned}$$

Indeed, real-valued solutions of (1.1) are also solutions of (1.2).

In this paper, we continue the study of the well-posedness of the periodic complex-valued mKdV equation (1.1) initiated in [4], by adapting the method introduced by Deng et al. [9] to the mKdV equation (1.1). Before stating our main results, we review the known well-posedness theory for the mKdV equation (1.1). When posed on the real line, (1.1) satisfies the following scaling symmetry: if *u* is a solution of (1.1) then $u^\lambda (t,x) = \lambda u(\lambda ^3t, \lambda x)$, $\lambda >0$, is also a solution of (1.1). This symmetry induces the scaling critical regularity $s_{\text {crit}} = - \frac{1}{2}$ in the sense that the ${\dot{H}}^{-\frac{1}{2}}({\mathbb {R}})$-norm is left invariant under the scaling. Consequently, it is commonly conjectured that the mKdV equation (1.1) is locally well-posed in the subcritical regime, i.e., in $H^s({\mathcal {M}})$ for $s>-\frac{1}{2}$, ${\mathcal {M}} = {\mathbb {R}}$ or ${\mathbb {T}}$. On the real line, ${\mathcal {M}}={\mathbb {R}}$, the complex-valued mKdV equation (1.1) has been a long standing topic of interest [11, 13, 16, 22]. In a recent breakthrough, Harrop-Griffiths et al. [16] showed the optimal global well-posedness of (1.1) in $H^s({\mathbb {R}})$ for $s>-\frac{1}{2}$ by exploiting the complete integrability of the equation.

In the periodic setting, ${\mathcal {M}}={\mathbb {T}}$, the real-valued mKdV equation (1.2) has garnered more attention than its complex-valued counterpart (1.1) [1, 3, 5, 6, 20, 21, 26–29, 35, 36]. Exploiting the conservation of mass $\mu \big (u(t)\big ) = \frac{1}{2\pi } \int _{\mathbb {T}}|u(t)|^2 dx$, Bourgain [1] introduced the first renormalized mKdV equation (mKdV1):

1.3 $$\begin{aligned} \partial _t u + \partial _x^3 u = \pm \big ( |u|^2 - \mu (u) \big )\partial _x u, \end{aligned}$$

and established its local well-posedness in $H^s({\mathbb {T}})$ for $s\ge \frac{1}{2}$. Note that mKdV1 (1.3) is equivalent to mKdV (1.1) in $L^2({\mathbb {T}})$ in the following sense: $u\in C\big ({\mathbb {R}};L^2({\mathbb {T}})\big )$ is a solution of (1.1) if and only if ${\mathcal {G}}_1(u)(t,x) := u \big (t,x\mp \mu \big (u(t)\big )t \big )$ is a solution of (1.3). The failure of $C^3$-continuity of the solution map [3, 5] outside $H^\frac{1}{2}({\mathbb {T}})$ requires more robust techniques to improve Bourgain’s result. In particular, the best known result in the Sobolev scale is due to Kappeler and Topalov [21] who exploited the completely integrable structure of (1.2). They showed that the real-valued defocusing mKdV equation, (1.2) with ‘$+$’, is globally well-posed in $H^s({\mathbb {T}})$ for $s\ge 0$. These solutions are almost periodic in time and the data-to-solution map extends continuously from smooth solutions to solutions in $H^s({\mathbb {T}})$ (see [21, 25, 32] for more details on this notion of solution). In [26], Molinet showed that the solutions in [21] are indeed distributional solutions and proved the ill-posedness of (1.2) below $L^2({\mathbb {T}})$ in the sense of failure of continuity of the solution map (see also [35]). This ill-posedness result motivated the study of (1.2) in alternative function spaces, namely in the Fourier–Lebesgue spaces ${\mathcal {F}}L^{s,p}({\mathbb {T}})$ defined by the following norm

1.4 $$\begin{aligned} \Vert f\Vert _{{\mathcal {F}}L^{s,p}} = \Vert \langle n \rangle ^s \widehat{f} (n) \Vert _{\ell ^p_n}, \end{aligned}$$

where $\langle \cdot \rangle = (1 + |\cdot |^2)^\frac{1}{2}$. In fact, Kappeler and Molnar [20] established local well-posedness of the real-valued defocusing mKdV1 (1.3) in ${\mathcal {F}}L^{s,p}({\mathbb {T}})$ for $s\ge 0$ and $1 \le p <\infty$ (see also [29]). These solutions are the unique limit of classical solutions, as those in [21], almost periodic in time, and global-in-time for small initial data. Since ${\dot{{\mathcal {F}}L}}^{s,p}({\mathbb {R}})$ scales like ${\dot{H}}^\sigma ({\mathbb {R}})$ for $\sigma = s + \frac{1}{p} - \frac{1}{2}$, this result is almost critical. Lastly, note that the results in [20, 21] also extend to the real-valued focusing equation (with ‘−’) for small initial data.

In our previous work [4], we established a sharp contrast between the real and the complex-valued settings. In particular, we showed that $H^\frac{1}{2}({\mathbb {T}})$ is the limit for the local well-posedness of the complex-valued mKdV equation (1.1), as it is ill-posed outside $H^\frac{1}{2}({\mathbb {T}})$ in the sense of non-existence of solutions. This result is closely related to the momentum

1.5 $$\begin{aligned} P\big (u(t)\big ) = \frac{1}{2\pi } {{\,\mathrm{Im}\,}}\int _{\mathbb {T}}\overline{u}(t) \partial _xu(t) \, dx, \end{aligned}$$

a formally conserved quantity of the equation. Note that the momentum is only well-defined in the Fourier–Lebesgue spaces ${\mathcal {F}}L^{s,p}({\mathbb {T}})$ which embed continuously in $H^\frac{1}{2}({\mathbb {T}})$, i.e., when $\frac{1}{2} \le s <1$ and $1 \le p < \frac{1}{1-s}$, or $s \ge 1$ and $1 \le p <\infty$. Outside $H^\frac{1}{2}({\mathbb {T}})$, the momentum may no longer be conserved or even finite. In our first main result, we extend the ill-posedness of the complex-valued mKdV equation (1.1) to a larger class of spaces.

#### Theorem 1.1

Let $\frac{1}{2} \le s< 1$ and $\frac{1}{1-s}\le p < \infty$. Suppose that $u_0\in {\mathcal {F}}L^{s,p}({\mathbb {T}})$ has infinite momentum in the sense that

1.6 $$\begin{aligned} \limsup _{N\rightarrow \infty } |P({\mathbf {P}}_{\le N} u_0 )| = \infty , \end{aligned}$$

where ${\mathbf {P}}_{\le N}$ denotes the projection onto the spatial frequencies $\{|n| \le N\}$ (see Definition 2.1). Then, for any $T>0$, there exists no distributional solution $u\in C([-T,T];{\mathcal {F}}L^{s,p}({\mathbb {T}}))$ to the complex-valued mKdV equation (1.1) satisfying the following conditions:

(i) $$u\vert _{t=0} = u_0$$,
(ii) The smooth global solutions $\{u_N\}_{N\in {\mathbb {N}}}$ of mKdV (1.1), with $u_N\vert _{t=0} = {\mathbf {P}}_{\le N}u_0$, satisfy $u_N \rightarrow u$ in $C([-T,T];{\mathcal {F}}L^{s,p}({\mathbb {T}}))$.

#### Remark 1.2

(i) The momentum is identically zero for real-valued solutions, therefore it does not play a role in the low regularity well-posedness of the real-valued mKdV equation (1.2). Consequently, the ill-posedness result in Theorem 1.1 is a phenomenon specific to the complex-valued mKdV equation (1.1).
  In addition, note that for $2<p<\infty$, $H^\frac{1}{2} ({\mathbb {T}}) \subsetneq {\mathcal {F}}L^{\frac{1}{2},p}({\mathbb {T}}) \subsetneq L^2({\mathbb {T}})$, therefore Theorem 1.1 applies to data outside $H^\frac{1}{2}({\mathbb {T}})$.
(ii) In Remark 1.2 (iii) in [28], the authors claim that local well-posedness in $H^s({\mathbb {T}})$ for $s>\frac{1}{3}$ extends to the complex-valued solutions of (1.1). In light of the ill-posedness result in Theorem 1.1, this does not seem to be the case. In fact, it appears that the only local well-posedness result for the real-valued mKdV equation (1.2) which extends to its complex-valued counterpart (1.1), prior to the second renormalization in (1.8), is that of Bourgain [1].

In view of the ill-posedness of (1.1), in [4], we proposed an alternative model for the complex-valued mKdV equation (1.1) at low regularity. Similarly to the first gauge transform ${\mathcal {G}}_1$ which exploited the conservation of mass, we introduced a second renormalization of the equation through the following gauge transformation depending on the momentum

1.7 $$\begin{aligned} {\mathcal {G}}_2(u)(t,x) = e^{\mp iP(u)t} u(t,x). \end{aligned}$$

If $u \in C\big ({\mathbb {R}}; H^\frac{1}{2}({\mathbb {T}})\big )$, the momentum is finite and conserved $P\big (u(t)\big ) = P(u_0)$, thus the gauge transformation ${\mathcal {G}}_2$ is invertible and *u* solves (1.1) if and only if ${\mathcal {G}}_2 \circ {\mathcal {G}}_1 ( u )$ solves the second renormalized mKdV equation (mKdV2):

1.8 $$\begin{aligned} \partial _t u + \partial _x^3 u = \pm \Big (|u|^2 \partial _x u - \mu (u) \partial _x u - i P(u) u \Big ). \end{aligned}$$

The effect of the gauge transformation ${\mathcal {G}}_2$ is to remove certain resonant frequency interactions in the nonlinearity which are responsible for the ill-posedness result in Theorem 1.1. We proved that, unlike mKdV1 (1.3), mKdV2 (1.8) is locally well-posed in ${\mathcal {F}}L^{s,p}({\mathbb {T}})$ for $\frac{1}{2} \le s < \frac{3}{4}$ and $1\le p < \frac{4}{3-4s}$, or $s\ge \frac{3}{4}$ and $1 \le p <\infty$. Our main result in this paper is the improved global well-posedness of (1.8), shown without exploiting the complete integrability of the equation.

#### Theorem 1.3

The mKdV2 equation (1.8) is locally well-posed in ${\mathcal {F}}L^{s,p}({\mathbb {T}})$ for any $s\ge \frac{1}{2}$ and $1\le p <\infty$. Moreover, the data-to-solution map is locally Lipschitz continuous.

#### Remark 1.4

(i) The solutions constructed in Theorem 1.3 satisfy the Duhamel formulation:
  $$\begin{aligned} u(t) = S(t) u_0 \pm \int _0^t S(t-t') {\mathcal {N}}(u, \overline{u},u)(t') \, dt', \end{aligned}$$
  for $t\in [-T,T]$ for some $T = T(\Vert u_0\Vert _{{\mathcal {F}}L^{\frac{1}{2},p}}) >0$, where *S*(*t*) denotes the linear propagator and ${\mathcal {N}}(u, \overline{u}, u)$ corresponds to the right-hand side of (1.8). See Sect. 2 for further details.
(ii) Theorem 1.3 is sharp with respect to the method, since the data-to-solution map fails to be locally uniformly continuous in ${\mathcal {F}}L^{s,p}({\mathbb {T}})$ for any $s<\frac{1}{2}$, $1\le p <\infty$ (see [4]). Without imposing uniform continuous dependence on the initial data, we expect it to be possible to lower *s* in Theorem 1.3. In a forthcoming work, we intend to pursue the question of local well-posedness of mKdV2 (1.8) in ${\mathcal {F}}L^{s,p}({\mathbb {T}})$ for $s<\frac{1}{2}$ by combining the method introduced by Deng et al. [9] and the energy method in [27, 28, 36].
(iii) To show Theorem 1.3, we follow the method introduced by Deng et al. [9], which is based on constructing solutions *u* with a particular structure. As a consequence, uniqueness holds conditionally in a sub-manifold of $X^{\frac{1}{2},\frac{1}{2}}_{p,2-}$ (see Definition 2.2) determined by the structure imposed on *u*. In Sobolev spaces, unconditional uniqueness holds in $H^s({\mathbb {T}})$ for $s\ge \frac{1}{3}$ (see [24, 27]). It would be of interest to consider the problem of unconditional uniqueness of mKdV2 (1.8) in the Fourier–Lebesgue spaces.
(iv) For the range of (*s*, *p*) in Theorem 1.3, the mass $\mu (u)$ is conserved and therefore one can still establish local well-posedness of (1.8) without removing the term $\mu (u)\partial _xu$ from the nonlinearity at the cost of losing the local Lipschitz continuity of the solution map. In contrast, this renormalization is essential in [20] when taking data in ${\mathcal {F}}L^{0,p}({\mathbb {T}})$ with $2< p <\infty$, for example. Analogously, the second renormalization introduced by ${\mathcal {G}}_2$ is crucial in Theorem 1.3 when $\frac{1}{2} \le s < 1$ and $\frac{1}{1-s} \le p <\infty$. In the remaining regimes for (*s*, *p*), since the initial data is in $H^\frac{1}{2}({\mathbb {T}})$ and the momentum is conserved, the renormalization is only required to guarantee the local Lipschitz continuity in Theorem 1.3.

Using the a priori bounds established by Oh and Wang [31], which generalize the result by Killip et al. [23] to the Fourier–Lebesgue setting, we extend the solutions in Theorem 1.3 globally-in-time.

#### Theorem 1.5

The mKdV2 equation (1.8) is globally well-posed in ${\mathcal {F}}L^{s,p}({\mathbb {T}})$ for $s\ge \frac{1}{2}$ and $1\le p <\infty$.

For real-valued solutions *u*, the momentum $P(u) \equiv 0$ which implies that ${\mathcal {G}}_2(u) \equiv u$. Consequently, the previous results on the complex-valued mKdV2 equation (1.3) in Theorems 1.3 and 1.5 also apply to the real-valued setting.

#### Corollary 1.6

The real-valued mKdV1 equation (1.3) is globally well-posed in ${\mathcal {F}}L^{s, p}({\mathbb {T}})$ for $s\ge \frac{1}{2}$ and $1\le p <\infty$.

#### Remark 1.7

In [20], Kappeler–Molnar established the global well-posedness of the real-valued mKdV1 equation (1.3) with small initial data. The Corollary 1.6 extends this result (with limited range of *s*) to the large data setting. Furthermore, our solutions satisfy the Duhamel formulation, establishing that the solutions in ${\mathcal {F}}L^{s,p}({\mathbb {T}})$ for $s\ge \frac{1}{2}$ constructed in [20] are distributional solutions. The remaining solutions in [20] are not yet known to be distributional.

We conclude this section by stating some further remarks.

#### Remark 1.8

(i) The ill-posedness result in Theorem 1.1 follows an argument by Guo and Oh [15]. The proof combines the local well-posedness of mKdV2 (1.8) in Theorem 1.3 and the rapid oscillation of the phase in the gauge transformation ${\mathcal {G}}_2$ (1.7), due to the assumption on the momentum (1.6). Thus, since Theorem 1.1 follows from [4, 15], we omit the details.
(ii) The scaling heuristics described above can be transported to the Fourier–Lebesgue setting. The critical regularity is given by $s_{\text {crit}} (p)= - \frac{1}{p}$. We can also compare the scaling of the two families of spaces to conclude that ${\dot{{\mathcal {F}}L}}^{s,p}({\mathbb {R}})$ scales like ${\dot{H}}^\sigma ({\mathbb {R}})$ for $\sigma = s - \frac{1}{2} + \frac{1}{p}$. From these heuristics, we see that the results in Theorem 1.3 and Corollary 1.6 are at the scale of $L^2({\mathbb {T}})$. At this time, we do not know how to prove an almost critical result for (1.1), but we hope to pursue this question in the future.

#### Remark 1.9

In [18], Herr introduced a gauge transformation to study the low regularity Cauchy problem for the derivative nonlinear Schrödinger equation (DNLS). This gauge transformation removes singular contributions from the nonlinearity, as ${\mathcal {G}}_2$ introduced in (1.7). Unlike that of DNLS, the gauge transformation ${\mathcal {G}}_2$ depends explicitly on the momentum, which is ill-defined at low regularity, preventing us from freely recovering solutions of (1.1) from those of (1.8). We overcame this difficulty, not present for DNLS, by introducing the following notion of finite momentum at low regularity: we say that *f* has finite momentum if

$$\begin{aligned} P({\mathbf {P}}_{\le N} f) \text { converges as } N\rightarrow \infty , \end{aligned}$$

and denote the limit by *P*(*f*).

By imposing this notion of finite momentum to initial data in ${\mathcal {F}}L^{s,p}({\mathbb {T}})$ for $s\ge \frac{1}{2}$ and $1\le p <3$, we showed that the corresponding solutions of mKdV2 (1.8) have finite and conserved momentum. The restriction follows from an energy estimate, which we do not know how to improve at the moment. As a consequence of conservation of momentum, we proved existence of distributional solutions of mKdV (1.1) by using the gauge transformation ${\mathcal {G}}_2$ and a limiting argument. We expect a similar result to hold for the full range of well-posedness, $s\ge \frac{1}{2}$ and $1\le p<\infty$, if the momentum of the initial data is finite. Since the main focus of this paper is on improving the previous well-posedness result of mKdV2 (1.8), we will not discuss further how to recover solutions of the complex-valued mKdV equation (1.1) from those of mKdV2 (1.8).

### Outline of the Strategy

In our previous work on the complex-valued mKdV equation [4], we proved local well-posedness of mKdV2 (1.8) in ${\mathcal {F}}L^{s,p}({\mathbb {T}})$ for $s\ge \frac{1}{2}$ and $1\le p <4$ by using the Fourier restriction norm method. The solutions are constructed through a contraction mapping argument on the Duhamel formulation

1.9 $$\begin{aligned} u(t) = S(t) u_0 \pm \int _0^t S(t-t') {\mathcal {N}}(u, \overline{u}, u) (t') \, dt' =: S(t)u_0 \pm D {\mathcal {N}}(u, \overline{u}, u)(t), \end{aligned}$$

where *S*(*t*) denotes the linear propagator, *D* the Duhamel operator, and

$$\begin{aligned} {\mathcal {N}}(u_1,u_2,u_3) = \partial _xu_1 \Big ( u_2 u_3 - \frac{1}{2\pi } \int _{\mathbb {T}}u_2 u_3 \, dx \Big ) - i \Big (\frac{1}{2\pi }{{\,\mathrm{Im}\,}}\int _{\mathbb {T}}\partial _xu_1\, u_2 \, dx \Big )u_3. \end{aligned}$$

Note that the nonlinearity of mKdV2 (1.8) corresponds to ${\mathcal {N}}(u, \overline{u},u)$. In particular, we look for solutions in some $X^{s,b}$-spaces adapted to the Fourier–Lebesgue setting (see Definition 2.2). A key ingredient in proving the local well-posedness is a nonlinear estimate of the form

1.10 $$\begin{aligned} \big \Vert D {\mathcal {N}}(u_1,u_2,u_3) \big \Vert _{X^{s,b}_{p,q} } \lesssim \prod _{j=1}^3 \Vert u_j\Vert _{X^{s,b}_{p,q}}, \end{aligned}$$

for $b=\frac{1}{2}$ and $q=2$. The main difficulty resides in controlling the derivative in the nonlinearity. Since the Duhamel operator has smoothing in time but not space, we want to exploit the multilinear dispersion by using the modulations, i.e., the weights $\langle \tau - n^3 \rangle$ in the norm (2.1). The need to use the time smoothing to help control the time derivative imposes the restriction $1\le p <4$ and we do not know how to overcome it within this framework.

In this paper, we apply the method introduced by Deng et al. [9] to the mKdV2 equation (1.8) extending our previous local well-posedness result to ${\mathcal {F}}L^{s,p}({\mathbb {T}})$ for $s\ge \frac{1}{2}$ and $4\le p <\infty$. This method was introduced to improve the local well-posedness results of Grünrock and Herr [12] for DNLS. Motivated by the probabilistic setting, we construct solutions *u* centered around a smoother-in-time function *w*. Instead of solving the Duhamel formulation, we will solve a system of equations

1.11 $$\begin{aligned} {\left\{ \begin{array}{ll} u = w + F(u,w), \\ w = S(t)u_0 \pm D {\mathcal {N}}(u) - F(u,w), \end{array}\right. } \end{aligned}$$

where *F*(*u*, *w*) is a nonlinear functional to be determined. Centering solutions around a suitably chosen function was seen in the context of probabilistic PDEs (with random initial data or stochastic forcing), for example, in the works of Bourgain [2], Da Prato and Debussche [7] and Gubinelli et al. [14]. There, the center *w* is an explicitly known random object that introduces smoothing in space in the remainder pieces. The lack of randomness in our setting forces us to consider a moving center and solve the system (1.11). In particular, the first equation in (1.11) imposes structure to $u=u[w]$ parametrized by *w*, while the second finds the correct *w* for which *u* solves the Duhamel formulation (1.9).

The main difficulty in this method is choosing the correct structure for *u*, or equivalently, the correct nonlinear functional *F*(*u*, *w*). This choice, which is not prescribed by the method, must allow us to solve the first equation for *u* while avoiding the bad frequency regions for the nonlinear estimate (1.10). There are three main points in establishing and solving the system (1.11): (i) choosing the frequency regions of the nonlinear terms in *F*(*u*, *w*); (ii) modifying the Duhamel operator to induce smoothing in space; (iii) using second iteration to solve the equation for *w*.

We first consider (i). In certain regions of the frequency space, the nonlinear estimate (1.10) holds for any fixed $2 \le p <\infty$ and some $b=1-$ and $q = \infty -$ (see Remark 6.3 for more details). These frequency regions will be included in the equation for *w*, which we hope to solve for $w\in X^{s, 1-}_{p,\infty -} \subset X^{s, \frac{1}{2}}_{p,2-}$ and $u\in X^{s, \frac{1}{2}}_{p,2-} \subset C\big ({\mathbb {R}}; {\mathcal {F}}L^{s, p}({\mathbb {T}})\big )$. For the remaining frequency regions, we cannot show the trilinear estimate (1.10) in $X^{s,b}_{p,q} \subset C\big ({\mathbb {R}}; {\mathcal {F}}L^{s,p}({\mathbb {T}})\big )$, regardless of the choice of *b* and *q*. These contributions should then appear in *F*(*u*, *w*), in order to be estimated in the weaker $X^{s,\frac{1}{2}}_{p,2-}$-norm. In addition, we require the terms in *F*(*u*, *w*) to have a smoother *w* term associated with the derivative and the largest frequency. Consequently, *F*(*u*, *w*) includes terms that essentially look like the following

1.12 $$\begin{aligned} \sum _N {\mathbf {P}}_{ N} \partial _x w \cdot {\mathbf {P}}_{\ll N} \overline{u} \cdot {\mathbf {P}}_{\ll N} u , \qquad \sum _N {\mathbf {P}}_{ N} \partial _x w \cdot ({\mathbf {P}}_N \overline{w} \cdot {\mathbf {P}}_{\ll N} u+{\mathbf {P}}_N w \cdot {\mathbf {P}}_{\ll N} \overline{u}) ,\qquad \end{aligned}$$

where ${\mathbf {P}}_N$ and ${\mathbf {P}}_{\ll N}$ denote the Dirichlet projections onto the spatial frequencies $\{|n|\sim N\}$ and $\{|n| \ll N\}$, respectively; see Definition 2.1. These terms can roughly be seen as ‘paracontrolled’ by *w* (see [14] for details on paracontrolled distributions).

Unfortunately, the assumptions imposed on *F*(*u*, *w*) and the terms in (1.12) are not yet enough to show the estimate (1.10) for any $p\ge 2$. This leads us to (ii) and to the introduction of a modified Duhamel operator which not only has smoothing in time but also in space. The modification is introduced through a time convolution with a smooth function $\eta$ parameterized by the resonance relation $\Phi ({\overline{n}}_{123})$:

1.13 $$\begin{aligned} \int _0^t S(t-t') \eta \big ( \Phi ({\overline{n}}_{123}) (t-t') \big ) F(t') \, dt', \end{aligned}$$

where $\Phi ({\overline{n}}_{123}) = n^3 - n_1^3 - n_2^3 - n_3^3$. We then choose *F*(*u*, *w*) by applying the modified Duhamel operator in (1.13) to the terms in (1.12). Here, since the frequencies satisfy $|\Phi ({\overline{n}}_{123})| \gtrsim \max (|n_1|,|n_2|,|n_3|)^2$, the convolution with $\eta$ in (1.13) introduces negative powers of $|\Phi ({\overline{n}}_{123})|$ and consequently smoothing in space, at the cost of reduced smoothing in time (see Sect. 3 for more details). This smoothing effect allows us to solve the equation for *u* through a fixed point argument, for each fixed $w\in X^{s,1-}_{p,\infty -}$. Consequently, we obtain a function $u=u[w]$ parameterized by *w* that is not yet a solution to the mKdV2 equation (1.8). This will only follow after we have found the correct center *w*.

To solve the equation for *w*, we use a ‘partial’ iteration of the Duhamel formulation. For the terms that cannot be estimated directly in the $X^{s,1-}_{p,\infty -}$-norm, we replace $u=u[w]$ by its equation $w + F(u,w)$ first in the entries associated with the derivative and then the largest frequencies. This strategy induces smoothing, by introducing terms that depend on the modified Duhamel operator (1.13) and more *w* terms, at the cost of increasing the multilinearity of the terms begin estimated. This strategy resembles the second iteration method used by Bourgain [3], Oh [30] and Richards [33], for example. In particular, this leads to new cubic, quintic and septic terms that we can estimate in the stronger norm (see Sects. 4, 6).

In summary, the choice of *F*(*u*, *w*) requires a delicate balance between being able to solve the first equation for *u*, but also inducing sufficient spatial smoothing when using second iteration to solve the equation for *w*. This choice allows us to show the relevant estimates for any $2\le p <\infty$, even when we must use the smoothing in time to gain smoothing in space.

### Outline of the Paper

In Sect. 2 we introduce the relevant function spaces and some auxiliary results. In Sect. 3 we introduce the modified Duhamel operator and show relevant kernel estimates. In Sect. 4 we establish the equation for *u*. In addition, we explain how to use second iteration to obtain the equation for *w*, which is included in detail in “Appendix A”. The relevant estimates for the nonlinear terms in the equation for *u* are shown in Sect. 5. Lastly, Sect. 6 is dedicated to solving the equation for *w*.

## Preliminaries

### Notations and Function Spaces

We start by introducing some useful notations. We will use $\varphi : {\mathbb {R}}\rightarrow {\mathbb {R}}$ to denote a smooth time cutoff function, equal to 1 on $[-1,1]$ and 0 outside $[-2,2]$ and $\varphi _T (t) := \varphi (T^{-1}t)$, for $0\le T \le 1$. Lastly, let $A\lesssim B$ denote an estimate of the form $A\le CB$ for some constant $C>0$. Similarly, $A\sim B$ will denote $A\lesssim B$ and $B\lesssim A$, while $A\ll B$ will denote $A\le \varepsilon B$, for some small constant $0<\varepsilon \ll 1$. The notations $a+$ and $a-$ represent $a+\varepsilon$ and $a-\varepsilon$ for arbitrarily small $\varepsilon >0$, respectively.

Our conventions for the Fourier transform are as follows. The Fourier transform of $u: {\mathbb {R}}\times {\mathbb {T}}\rightarrow {\mathbb {C}}$ with respect to the space and time variable, respectively, are given by

$$\begin{aligned} {{\mathcal {F}}}_x u(t,n) = \frac{1}{2\pi } \int _{\mathbb {T}}u(t,x) e^{- inx} \ dx, \qquad {{\mathcal {F}}}_t u(\tau ,x) = \frac{1}{2\pi }\int _{\mathbb {R}}u(t,x) e^{-it\tau } \ dt. \end{aligned}$$

The space-time Fourier transform is denoted by ${{\mathcal {F}}}= {{\mathcal {F}}}_t {{\mathcal {F}}}_x$. We will also use $\widehat{u}$ to denote ${{\mathcal {F}}}_x u$, ${{\mathcal {F}}}_t u$ and ${{\mathcal {F}}}_{t,x} u$, but it will become clear which one it refers to from context, namely from the use of the spatial and time Fourier variables *n* and $\tau$, respectively.

#### Definition 2.1

Let $0 \le R < \infty$ and denote by ${\mathbf {P}}_{\le R}$ the Dirichlet projection onto spatial frequencies $\{|n| \le R\}$ defined as follows

$$\begin{aligned} {\mathbf {P}}_{\le R} f (x) : = D_R *f (x) = \sum _{|n| \le R} \widehat{f}(n) e^{inx}, \end{aligned}$$

where $D_R(x) = \sum _{|n| \le N} e^{inx}$ is the Dirichlet kernel. We also define ${\mathbf {P}}_{\ll R}:= {\mathbf {P}}_{\le \frac{1}{2} R}$ and ${\mathbf {P}}_{R} := {\mathbf {P}}_{\le R} - {\mathbf {P}}_{\le \frac{1}{2} R}$.

Now, we focus on the relevant spaces of functions. Let ${\mathcal {S}}({\mathbb {R}}\times {\mathbb {T}})$ denote the space of functions $u:{\mathbb {R}}\times {\mathbb {R}}\rightarrow {\mathbb {C}}$, with $u\in C^\infty ({\mathbb {R}}\times {\mathbb {T}})$ which satisfy

$$\begin{aligned} u(t,x+2\pi ) = u(t,x), \quad \sup _{(t,x) \in {\mathbb {R}}\times {\mathbb {T}}} | t^\alpha \partial _t^\beta \partial _x^\gamma u(t,x)| < \infty , \quad \alpha ,\beta ,\gamma \in {\mathbb {Z}}_{\ge 0}. \end{aligned}$$

#### Definition 2.2

(See [1, 12]) Let $s,b\in {\mathbb {R}}$, $1\le p,q\le \infty$. The space $X^{s,b}_{p,q}({\mathbb {R}}\times {\mathbb {T}})$, abbreviated $X^{s,b}_{p,q}$, is defined as the completion of ${\mathcal {S}}({\mathbb {R}}\times {\mathbb {T}})$ with respect to the norm

2.1 $$\begin{aligned} \Vert u\Vert _{X^{s,b}_{p,q}} = \big \Vert \langle n \rangle ^s \langle \tau - n^3 \rangle ^b \widehat{u}(\tau , n) \big \Vert _{\ell ^p_n L^q_\tau }. \end{aligned}$$

When $p=q=2$, the $X^{s,b}_{p,q}$-spaces defined above reduce to the standard $X^{s,b}$-spaces.

Before proceeding, we introduce the relevant spaces of functions and associated parameters. Let $0< \delta \ll 1$ be a small parameter to be chosen later, depending on $2< p <\infty$. We introduce the following parameters

$$\begin{aligned} b_0&= 1 - 2 \delta ,&b_1&= 1 - \delta , \\ q_0&= \frac{1}{4\delta },&q_1&= \frac{1}{4.5\delta }, \\ \frac{1}{r_0}&= \frac{1}{2} + \delta ,&\frac{1}{r_1}&= \frac{1}{2} + 2\delta ,&\frac{1}{r_2}&= \frac{1}{2} + 3\delta . \end{aligned}$$

Note that $b_0 < b_1$, $q_1 < q_0$ and $r_2< r_1 < r_0$. We will focus on showing the result for the endpoint $s=\frac{1}{2}$, see Remark 4.4 for more details. Consequently, we will conduct our analysis in the following $X^{s,b}_{p,q}$-spaces:

$$\begin{aligned} Y_0&= X^{\frac{1}{2}, \frac{1}{2}}_{p, r_0} ({\mathbb {R}}\times {\mathbb {T}}),&Y_1&= X^{\frac{1}{2}, \frac{1}{2}}_{p, r_1} ({\mathbb {R}}\times {\mathbb {T}}), \\ Z_0&= X^{\frac{1}{2}, b_0}_{p, q_0} ({\mathbb {R}}\times {\mathbb {T}}),&Z_1&= X^{\frac{1}{2}, b_1}_{p,q_0} ({\mathbb {R}}\times {\mathbb {T}}). \end{aligned}$$

Note that $$Z_0 \subset Y_0 \subset C\big ({\mathbb {R}}; {\mathcal {F}}L^{\frac{1}{2},p}({\mathbb {T}})\big )$$.

### Nonlinearity and Notion of Solution

The nonlinearity of (1.8) has the following spatial Fourier transform

2.2 $$\begin{aligned} \sum _{\begin{array}{c} n=n_1+n_2+n_3,\\ \Phi ({\overline{n}}_{123}) \ne 0 \end{array}} in_1 \widehat{u}(n_1) \widehat{\overline{u}}(n_2) \widehat{u} (n_3) - in |\widehat{u}(n)|^2 \widehat{u}(n), \end{aligned}$$

where ${\overline{n}}_{123} = (n_1,n_2,n_3)$ and $\Phi =\Phi ({\overline{n}}_{123})$ denotes the resonance relation

$$\begin{aligned} \Phi ({\overline{n}}_{123}) = n^3 - n_1^3 - n_2^3 - n_3^3 = 3(n_1+n_2)(n_1+n_3)(n_2+n_3), \end{aligned}$$

where the factorization holds if $n=n_1+n_2+n_3$. Consider the following trilinear operators

2.3 $$\begin{aligned} {{\mathcal {F}}}_x\big ( \mathcal {NR}_{\ge }(u_1,u_2,u_3) \big )(n)&= \sum _{\begin{array}{c} n=n_1+n_2+n_3,\\ \Phi ({\overline{n}}_{123}) \ne 0, \\ |n_2| \ge |n_3| \end{array}} in_1 \widehat{u}_1(n_1) \widehat{u}_2 (n_2) \widehat{u}_3(n_3), \nonumber \\ {{\mathcal {F}}}_x \big ( {\mathcal {R}}(u_1,u_2,u_3) \big ) (n)&= - in \widehat{u}_1 (n) \overline{\widehat{u}}_2 ( n) \widehat{u}_3 (n), \end{aligned}$$

and $\mathcal {NR}_>$ where we impose $|n_2| > |n_3|$ on the right-hand side of (2.3). Consequently, we can decompose the nonlinearity (2.2) into non-resonant and resonant contributions

$$\begin{aligned} {\mathcal {N}}(u, \overline{u}, u) = \mathcal {NR}_{\ge }(u, \overline{u}, u) + \mathcal {NR}_>(u,u,\overline{u})+ {\mathcal {R}}(u,u,u). \end{aligned}$$

Note that if $n_j$ is the spatial frequency corresponding to $u_j$, $j=1,2,3$, in $\mathcal {NR}_\ge$ (2.3), then $|n_2| \ge |n_3|$ and the sums are taken over the following set

$$\begin{aligned} {\mathbb {X}}(n) = \big \{(n_1,n_2,n_3)\in {\mathbb {Z}}^3 : \ n=n_1+n_2+n_3, \ |n_2| \ge |n_3|, \ \Phi ({\overline{n}}_{123}) \ne 0 \big \}, \end{aligned}$$

with the additional assumption $|n_2| > |n_3|$ for $\mathcal {NR}_>$. Consequently, we want to consider the following subregions of ${\mathbb {X}}(n)$:

$$\begin{aligned} {\mathbb {X}}_{A}(n)&= \big \{ (n_1,n_2,n_3) \in {\mathbb {X}}(n): \ |n_2| \ll |n_1| \big \}, \\ {\mathbb {X}}_{B}(n)&= \big \{ (n_1,n_2,n_3)\in {\mathbb {X}}(n): \ |n_3| \ll \min (|n|, |n_1|) \le \max (|n|, |n_1|) \sim |n_2| \big \}, \\ {\mathbb {X}}_{C}(n)&= \big \{ (n_1,n_2,n_3) \in {\mathbb {X}}(n): \ |n| \lesssim |n_3| \ll |n_1| \big \}, \\ {\mathbb {X}}_{D}(n)&= \big \{ (n_1,n_2,n_3) \in {\mathbb {X}}(n): \ |n_1| \lesssim |n_3| \big \}. \end{aligned}$$

For $*\in \{A, B, C, D\}$, let $\mathcal {NR}_{*, \ge }, \mathcal {NR}_{*, >}$ denote the restriction of the operators to ${\mathbb {X}}_*(n)$. We can write the non-resonant contributions of the nonlinearity as

$$\begin{aligned} \mathcal {NR}_{\ge } = \mathcal {NR}_{A, \ge } + \mathcal {NR}_{B, \ge } + \mathcal {NR}_{C, \ge } + \mathcal {NR}_{D, \ge } \end{aligned}$$

and equivalently for $\mathcal {NR}_{>}$. We also introduce the following notation

2.4 $$\begin{aligned} {\mathbb {X}}_*^\mu (n) := \big \{ {\overline{n}}_{123} \in {\mathbb {X}}_*(n): \ \Phi ({\overline{n}}_{123} ) = \mu \big \}. \end{aligned}$$

The following lemma clarifies the relation between the frequencies in the subregions introduced.

#### Lemma 2.3

The sets ${\mathbb {X}}_*(n)$, where $*\in \{A, B, C, D\}$, satisfy the following properties:

(i) $$(n_1, n_2, n_3)\in {\mathbb {X}}_A(n) \implies |n_3| \le |n_2| \ll |n_1|\sim |n| $$;
(ii) $$(n_1, n_2, n_3)\in {\mathbb {X}}_B(n) \implies |n_3| \ll |n| \lesssim |n_1| \sim |n_2| $$ or $$|n_3| \ll |n_1| \ll |n| \sim |n_2|$$;
(iii) $$(n_1, n_2, n_3)\in {\mathbb {X}}_C(n) \implies |n| \lesssim |n_3| \ll |n_2| \sim |n_1| $$;
(iv) $$(n_1, n_2, n_3)\in {\mathbb {X}}_D(n) \implies |n_1| \lesssim |n_3| \le |n_2| $$;
(v) $$(n_1, n_2, n_3)\in {\mathbb {X}}_A(n) \cup {\mathbb {X}}_B(n) \cup {\mathbb {X}}_C(n) \implies |\Phi ({\overline{n}}_{123})| \gtrsim \max (|n_1|, |n_2|)^2$$;
(vi) $$(n_1, n_2, n_3) \in {\mathbb {X}}_D(n) \implies |\Phi ({\overline{n}}_{123})| \gtrsim |n_2|$$ and $$\langle n \rangle ^\frac{1}{2} |n_1| \lesssim (\langle n_1 \rangle \langle n_2 \rangle \langle n_3 \rangle )^\frac{1}{2}$$.

#### Remark 2.4

Note that the nearly-resonant case when $|n_1|\sim |n_2|\sim |n_3|$ and $|\Phi ({\overline{n}}_{123})|\gtrsim \max (|n_1|, |n_2|, |n_3|)$ is included in ${\mathbb {X}}_D(n)$. There are other frequency interactions allowed in this region which are fully non-resonant, i.e., $|\Phi ({\overline{n}}_{123})| \gtrsim \max (|n_1| , |n_2|, |n_3|)^2$ holds. However, due to the condition in $\mathrm (vi)$, the resonance relation will not play a crucial role when estimating this contribution.

Lastly, we recall our notion of solution to (1.8). Let $D F(t,x) = \int _0^t S(t-t') F(t',x) \, dt'$ denote the Duhamel operator and its truncated version

$$\begin{aligned} {\mathcal {D}}F(t,x)&= \varphi (t) \cdot D \big ( \varphi (t') \cdot F(t',x) \big ) (t) = \varphi (t) \int _0^t S(t-t') \varphi (t') F(t',x) \, dt'. \end{aligned}$$

We say that $u\in C({\mathbb {R}}; {\mathcal {F}}L^{\frac{1}{2},p}({\mathbb {T}}))$ is a solution of (1.8) with initial data $u_0\in {\mathcal {F}}L^{\frac{1}{2}, p}({\mathbb {T}})$ if it satisfies the following integral equation, the Duhamel formulation^1^

2.5 $$\begin{aligned} u(t) = S(t) u_0 + D \mathcal {NR}(u, \overline{u}, u) (t) + D {\mathcal {R}}(u,u,u)(t). \end{aligned}$$

Since we are only concerned with local well-posedness, let $0< T \le 1$ and consider the truncated Duhamel formulation:

2.6 $$\begin{aligned} u(t) = \varphi \cdot S(t) u_0 + \varphi _T \cdot {\mathcal {D}}\mathcal {NR} (u, \overline{u}, u) (t) + \varphi _T \cdot {\mathcal {D}}{\mathcal {R}}(u,u,u)(t). \end{aligned}$$

### Auxiliary Results

The following proposition allows us to gain a small power of the time of existence *T*, needed to close the contraction mapping argument.

#### Lemma 2.5

Suppose that *F* is a smooth function such that $F(0)=0$. Then, we have the following estimates

$$\begin{aligned} \Vert \varphi _T\cdot F \Vert _{Y_0} \lesssim T^\theta \Vert F\Vert _{Y_1}, \qquad \Vert \varphi _T \cdot F \Vert _{Z_0} \lesssim T^\theta \Vert F\Vert _{Z_1}, \end{aligned}$$

for any $0<\theta \le \frac{\delta }{2}$ and $0<T\le 1$.

#### Proof

We want to estimate the following quantity

$$\begin{aligned} \Vert \varphi _T \cdot F \Vert _{X^{s,b}_{p,q}}&= \Big \Vert \langle n \rangle ^s \langle \tau \rangle ^b \big ( \widehat{\varphi }_T *_\tau \widehat{F}(\cdot +n^3,n) \big )(\tau ) \Big \Vert _{\ell ^p_n L^{q}_\tau }. \end{aligned}$$

Both estimates follow once we show

2.7 $$\begin{aligned} \Vert \langle \tau \rangle ^b \widehat{\varphi }_T *f (\tau ) \Vert _{L^q_\tau } \lesssim T^{\frac{1}{{\tilde{q}}} - \frac{1}{q}} \Vert \langle \tau \rangle ^b f (\tau ) \Vert _{L^{{\tilde{q}}}_\tau }, \end{aligned}$$

for *f* satisfying $\int _{\mathbb {R}}f(\tau ) \, d\tau = 0$, $1<{\tilde{q}}<q<\infty$ and $b<1<b+\frac{1}{{\tilde{q}}}$. To estimate the left-hand of (2.7) restricted to $\{|\tau | \ge T^{-1}\}$, it suffices to show

2.8 $$\begin{aligned} \bigg \Vert {{\,\mathrm{\int }\,}}_{\mathbb {R}}\frac{\langle \tau \rangle ^b}{\langle \lambda \rangle ^b} \mathbb {1}_{|\lambda | \ge T^{-1}} \widehat{\varphi }_T(\tau - \lambda ) f (\lambda ) \, d\lambda \bigg \Vert _{L^q_{\tau }} \lesssim T^{\frac{1}{{\tilde{q}}} - \frac{1}{q}} \Vert f\Vert _{L^{{\tilde{q}}}_\tau }. \end{aligned}$$

Using Young’s inequality with $1 + \frac{1}{q} = \frac{1}{{\tilde{q}}} + \frac{1}{r}$ gives

$$\begin{aligned} \text {LHS of }(6.11)&\lesssim \bigg \Vert {{\,\mathrm{\int }\,}}_{\mathbb {R}}\langle T(\tau -\lambda ) \rangle ^b T \widehat{\varphi }(T(\tau -\lambda )) f (\lambda ) \ d \lambda \bigg \Vert _{L^q_\tau } \lesssim T \Vert \langle T\tau \rangle ^b \widehat{\varphi }(T\tau ) \Vert _{L^r_{\tau }} \Vert f\Vert _{L^{{\tilde{q}}}_\tau }. \end{aligned}$$

The estimate follows from $T \Vert \langle T\tau \rangle ^b \widehat{\varphi }(T\tau ) \Vert _{L^r_\tau } \lesssim _\varphi T^{1-\frac{1}{r}} = T^{\frac{1}{{\tilde{q}}} - \frac{1}{q}}$.

To estimate the left-hand side of (2.7) restricted to $\{|\tau | \le T^{-1}\}$, using the fact that $\int _{\mathbb {R}}f(\tau ) \, d\tau = 0$, we note that

2.9 $$\begin{aligned} \widehat{\varphi }_T *\big ( \mathbb {1}_{|\tau |< T^{-1}} f \big ) (\tau ) =\!\!\!\!\! {{\,\mathrm{\int }\,}}_{|\lambda | < T^{-1}} \!\!\!\!\! f(\lambda ) T \big [ \widehat{\varphi }(T(\tau - \lambda )) - \widehat{\varphi }(T\tau ) \big ] \, d\lambda -\!\!\!\! {{\,\mathrm{\int }\,}}_{|\lambda |\ge T^{-1}} \!\!\!\!\! f(\lambda ) T\widehat{\varphi }(T\tau ) \, d\lambda . \end{aligned}$$

For the first contribution in (2.9), we distinguish between the regions $\{\tau : \, |\tau | \lesssim T^{-1}\}$ and $\{\tau : \, |\tau | \gg T^{-1}\}$, and use mean value theorem to obtain

$$\begin{aligned} {{\,\mathrm{\int }\,}}_{|\lambda |< T^{-1}} T |f(\lambda )| |\widehat{\varphi } (T(\tau -\lambda )) - \widehat{\varphi }(T\tau )| \, d\lambda \lesssim _{\varphi } \frac{T}{\langle T\tau \rangle ^\alpha } {{\,\mathrm{\int }\,}}_{|\lambda | <T^{-1}} |T\lambda | \, |f(\lambda )| \, d\lambda , \end{aligned}$$

for any $\alpha >0$. For the second contribution in (2.9), we have

$$\begin{aligned} {{\,\mathrm{\int }\,}}_{|\lambda | \ge T^{-1}} |f(\lambda )| T|\widehat{\varphi } (T\tau )| d\lambda&\lesssim _{\varphi } \frac{T}{\langle T\tau \rangle ^\alpha } {{\,\mathrm{\int }\,}}_{|\lambda | \ge T^{-1}} |f(\lambda )| \ d\lambda . \end{aligned}$$

Combining the estimates for the two contributions in (2.9), we obtain

$$\begin{aligned} \bigg \Vert \langle \tau \rangle ^b \widehat{\varphi }_T *\big ( \mathbb {1}_{|\tau | < T^{-1}} f \big ) (\tau ) \bigg \Vert _{L^q_\tau }&\lesssim \bigg \Vert \frac{T\langle \tau \rangle ^b}{\langle T\tau \rangle ^\alpha } \bigg \Vert _{L^q_\tau } \Vert \min (1, |T\lambda |) \langle \lambda \rangle ^{-b} \Vert _{L^r_\lambda } \Vert \langle \tau \rangle ^b f(\tau ) \Vert _{L^{{\tilde{q}}}_\tau }, \end{aligned}$$

by using Hölder’s inequality for the last step with $1 = \frac{1}{r} + \frac{1}{{\tilde{q}}}$. The first and second factors are controlled by $T^{1 - b - \frac{1}{q}}$ and $T^{b-\frac{1}{r}}$, respectively, given that $b>1 - \frac{1}{{\tilde{q}}}$ and $b<1$. The intended estimate follows.

$$\square $$

#### Remark 2.6

Lemma 2.5 will only be applied to functions of the form $F(t) = \int _0^t G(t') \, dt'$ which satisfy the assumption $F(0)=0$, namely the Duhamel operator ${\mathcal {D}}$ and the operators ${\mathbf {G}}, {\mathbf {B}}$ defined in Sect. 3.

The following lemma, adapted from [9], estimates the number of divisors of a given natural number.

#### Lemma 2.7

Fix $0<\varepsilon <1$, $\rho \ge 1$ and let $k,q\in {\mathbb {Z}}$ such that $|q| \gtrsim |k|^\varepsilon >0$. Then, the number of divisors $r\in {\mathbb {Z}}$ of *k* that satisfy $|r-q| \lesssim \rho$ is at most ${\mathcal {O}}_\varepsilon (\rho ^\varepsilon )$.

Lastly, recall the following well-known tool (see [10, Lemma 4.2]).

#### Lemma 2.8

Let $0\le \alpha \le \beta$ such that $\alpha + \beta >1$ and $\varepsilon >0$. Then, we have

$$\begin{aligned} \int _{\mathbb {R}}\frac{1}{\langle x-a \rangle ^{\alpha } \langle x-b \rangle ^{\beta } } dx \lesssim \frac{1}{ \langle a-b \rangle ^{\gamma } }, \end{aligned}$$

where

$$\begin{aligned} \quad \gamma = {\left\{ \begin{array}{ll} \alpha + \beta -1 , &{} \beta <1, \\ \alpha - \varepsilon , &{} \beta =1, \\ \alpha , &{} \beta >1. \end{array}\right. } \end{aligned}$$

## Splitting the Duhamel Operator

In this section, we explicitly establish the smoothing in time of the Duhamel operator by estimating its kernel. Moreover, we introduce the modified version of the Duhamel operator and the kernel estimate for the nonlinear contributions localized to ${\mathbb {X}}_A(n), {\mathbb {X}}_B(n)$.

### Proposition 3.1

The truncated Duhamel operator ${\mathcal {D}}$ has the following space-time Fourier transform

$$\begin{aligned} {{\mathcal {F}}}_{t,x} \big ( {\mathcal {D}}F \big )(\tau ,n) = {{\,\mathrm{\int }\,}}_{\mathbb {R}}K(\tau - n^3, \lambda - n^3) \widehat{F}(\lambda , n) \, d\lambda \end{aligned}$$

where the kernel *K* is given by the following expression

3.1 $$\begin{aligned} K(\tau , \lambda ) = -i {{\,\mathrm{\int }\,}}_{\mathbb {R}}\widehat{\varphi }(\mu - \lambda ) \frac{\widehat{\varphi }(\tau - \mu ) - \widehat{\varphi }(\tau )}{\mu } \, d\mu \end{aligned}$$

and satisfies the following estimates

3.2 $$\begin{aligned} | K (\tau , \lambda ) | \lesssim \bigg ( \frac{1}{\langle \tau - \lambda \rangle ^\alpha } + \frac{1}{\langle \tau \rangle ^\alpha } \bigg )\frac{1}{\langle \lambda \rangle } \lesssim \frac{1}{\langle \tau \rangle \langle \tau - \lambda \rangle } \end{aligned}$$

where $\alpha$ is a large enough positive number.

### Proof

We start by calculating the space-time Fourier transform of ${\mathcal {D}}F$,

$$\begin{aligned} {{\mathcal {F}}}_{t,x} \big ( {\mathcal {D}}F \big )(\tau ,n)&= {{\,\mathrm{\int }\,}}_{\mathbb {R}}\widehat{\varphi }(\tau - \mu ) {{\mathcal {F}}}_t \bigg ( \int _0^t e^{i(t-t')n^3} \varphi (t') \widehat{F}(t',n) \ dt' \bigg )(\mu ) \, d\mu . \end{aligned}$$

Using the fact that $\int _0^t f(t') \ dt' = \tfrac{1}{2} {{\,\mathrm{\int }\,}}_{\mathbb {R}}f(t') \big ( {{\,\mathrm{sgn}\,}}(t-t') + {{\,\mathrm{sgn}\,}}(t') \big ) \, dt'$, we have

$$\begin{aligned}&{{\mathcal {F}}}_t \bigg ( \int _0^t e^{i(t-t')n^3} \varphi (t') \widehat{F}(t',n) \ dt' \bigg )(\mu ) = \frac{1}{4\pi } {{\,\mathrm{\int }\,}}_{\mathbb {R}}e^{-it'\mu } \varphi (t') \widehat{F}(t',n) \, dt' {{\,\mathrm{\int }\,}}_{\mathbb {R}}\\&\qquad e^{-it(\mu -n^3)} {{\,\mathrm{sgn}\,}}(t) dt \\&\quad + \frac{1}{4\pi } {{\,\mathrm{\int }\,}}_{\mathbb {R}}e^{-it'n^3} \varphi (t') \widehat{F}(t',n) {{\,\mathrm{sgn}\,}}(t') \,dt' {{\,\mathrm{\int }\,}}_{\mathbb {R}}e^{-it(\mu -n^3)} \, dt. \end{aligned}$$

Consequently, since ${{\mathcal {F}}}_t({{\,\mathrm{sgn}\,}})(\tau ) = \frac{1}{i\pi \tau }$, we have

$$\begin{aligned}&{{\mathcal {F}}}_t \bigg ( \int _0^t e^{i(t-t')n^3} \varphi (t') \widehat{F}(t',n) \ dt' \bigg )(\mu ) = \frac{-i}{\mu -n^3} {{\,\mathrm{\int }\,}}_{\mathbb {R}}\widehat{\varphi }(\mu -\lambda ) \widehat{F}(\lambda , n) \, d\lambda \\&\quad -i\delta (\mu -n^3) {{\,\mathrm{\int }\,}}_{\mathbb {R}}{{\,\mathrm{\int }\,}}_{\mathbb {R}}\widehat{\varphi }(n^3 - \lambda - \mu ') \frac{1}{\mu '} \widehat{F}(\lambda , n) \, d\mu ' \, d\lambda . \end{aligned}$$

Calculating the convolution with $\widehat{\varphi }$, we get the intended expression. It remains to show the estimate on the kernel. In the region $\{|\mu |>1\}$, using Cauchy–Schwarz inequality and Lemma 2.8, we have

$$\begin{aligned} {{\,\mathrm{\int }\,}}_{|\mu |>1} \frac{|\widehat{\varphi }(\tau - \mu ) \widehat{\varphi }(\mu - \lambda ) |}{|\mu |} \, d\mu&\lesssim \bigg ({{\,\mathrm{\int }\,}}_{\mathbb {R}}\frac{d\mu }{\langle \tau -\mu \rangle ^{2\alpha } \langle \mu -\lambda \rangle ^{1+2\alpha }} \bigg )^\frac{1}{2} \bigg ( {{\,\mathrm{\int }\,}}_R \frac{d\mu }{\langle \mu \rangle ^2 \langle \mu -\lambda \rangle ^2} \bigg )^\frac{1}{2} \\&\lesssim \frac{1}{\langle \lambda \rangle \langle \tau - \lambda \rangle ^\alpha },\\ {{\,\mathrm{\int }\,}}_{|\mu |>1} \frac{|\widehat{\varphi }(\tau ) \widehat{\varphi }(\mu - \lambda )|}{ |\mu | } d\mu&\lesssim \frac{1}{\langle \tau \rangle ^\alpha }{{\,\mathrm{\int }\,}}_{\mathbb {R}}\frac{1}{\langle \mu -\lambda \rangle ^\alpha \langle \mu \rangle } \, d\mu \lesssim \frac{1}{\langle \lambda \rangle \langle \tau \rangle ^\alpha }, \end{aligned}$$

for any $\alpha >0$. In the region $\{|\mu |\le 1 \}$, we consider two subregions: $\{|\tau |\lesssim 1\}$ or $\{|\tau | \gg 1\}$. In both cases, using mean value theorem for any $\alpha >0$, we obtain

$$\begin{aligned} {{\,\mathrm{\int }\,}}_{|\mu |\le 1} |\widehat{\varphi }(\mu - \lambda )| \frac{|\widehat{\varphi }(\tau - \mu ) - \widehat{\varphi }(\tau )|}{|\mu |} d\mu&\lesssim _{\varphi } \frac{1}{\langle \lambda \rangle \langle \tau \rangle ^\alpha }. \end{aligned}$$

Combining the estimates, we get

$$\begin{aligned} |K(\tau , \lambda )| \lesssim \bigg ( \frac{1}{\langle \tau - \lambda \rangle ^\alpha } + \frac{1}{\langle \tau \rangle ^\alpha } \bigg )\frac{1}{\langle \lambda \rangle }. \end{aligned}$$

To show (3.2), note that $\langle \tau \rangle \lesssim \langle \tau -\lambda \rangle \langle \lambda \rangle$ and $\langle \tau -\lambda \rangle \lesssim \langle \tau \rangle \langle \lambda \rangle$. Thus, for $\alpha \ge 2$,

$$\begin{aligned} \bigg ( \frac{1}{\langle \tau - \lambda \rangle ^\alpha } + \frac{1}{\langle \tau \rangle ^\alpha } \bigg )\frac{1}{\langle \lambda \rangle } \lesssim \frac{1}{\langle \tau \rangle \langle \tau -\lambda \rangle ^{\alpha -1}} + \frac{1}{\langle \tau -\lambda \rangle \langle \tau \rangle ^{\alpha -1}} \lesssim \frac{1}{\langle \tau \rangle \langle \tau -\lambda \rangle }. \end{aligned}$$

$$\square $$

We want to split each of the non-resonant contributions ${\mathcal {D}}\mathcal {NR}_{*, \ge }, {\mathcal {D}}\mathcal {NR}_{*,>}$ for $*\in \{A,B\}$ into two components:

$$\begin{aligned} {\mathcal {D}}\mathcal {NR}_{*, \ge } = {\mathbf {G}}_{*, \ge } + {\mathbf {B}}_{*, \ge }, \qquad {\mathcal {D}}\mathcal {NR}_{*,>} = {\mathbf {G}}_{*,>} + {\mathbf {B}}_{*, >}. \end{aligned}$$

The ${\mathbf {G}}$ contributions will depend on the modified Duhamel operator. By introducing a convolution with a smooth function $\eta$ parameterized by the resonance relation $\Phi ({\overline{n}}_{123})$, we induce sufficient smoothing in space to control the derivative nonlinearity. Consider a Schwartz function $\eta$ satisfying

3.3 $$\begin{aligned} \widehat{\eta }(-1) = 0, \quad {\mathcal {H}}\widehat{\eta }(-1) =-1, \end{aligned}$$

where ${\mathcal {H}}$ denotes the Hilbert transform, i.e., principal value convolution with the function $\frac{1}{\tau }$. We define the operators ${\mathbf {G}}_{*, \ge }, {\mathbf {B}}_{*,\ge }$ through their spatial Fourier transform as follows

$$\begin{aligned}&{{\mathcal {F}}}_x\big ( {\mathbf {G}}_{*, \ge } (u_1,u_2,u_3) \big )(t,n) \\&\quad = \varphi (t) \sum _{\begin{array}{c} n=n_1+n_2+n_3,\\ {\overline{n}}_{123} \in {\mathbb {X}}_*(n),\\ |n_2| \ge |n_3| \end{array}}i n_1 \int _0^t e^{i(t-t')n^3} \eta \big (\Phi ({\overline{n}}_{123})(t-t')\big ) \varphi (t') \prod _{j=1}^3 \widehat{u}_j(t',n_j) \, dt',\\&{{\mathcal {F}}}_x\big ( {\mathbf {B}}_{*, \ge } (u_1,u_2,u_3)\big )(t,n) \\&\quad = \varphi (t) \sum _{\begin{array}{c} n=n_1+n_2+n_3,\\ {\overline{n}}_{123}\in {\mathbb {X}}_*(n), \\ |n_2| \ge |n_3| \end{array}} i n_1 \int _0^t e^{i(t-t')n^3} \big [1 - \eta \big (\Phi ({\overline{n}}_{123})(t-t')\big )\big ] \varphi (t')\prod _{j=1}^3 \widehat{u}_j(t',n_j) \, dt', \end{aligned}$$

with equivalent definitions for ${\mathbf {G}}_{*,>}, {\mathbf {B}}_{*,>}$ imposing the condition $|n_2| > |n_3|$ to the sum. In the following, we estimate the kernels for the above operators when $*\in \{A,B\}$.

### Proposition 3.2

Let $*\in \{A,B\}$. Then, the convolution operators ${\mathbf {G}}_{*, \ge }, {\mathbf {G}}_{*,>}$ have the following space-time Fourier transform

$$\begin{aligned}&{{\mathcal {F}}}_{t,x} \big ( {\mathbf {G}}_{*,\underset{(>)}{\ge }} (u_1,u_2,u_3) \big )(\tau ,n) \\&\quad = \sum _{\begin{array}{c} n=n_1+n_2+n_3,\\ {\overline{n}}_{123}\in {\mathbb {X}}_*(n), \\ |n_2| \underset{ (>)}{\ge } |n_3| \end{array} } n_1 {{\,\mathrm{\int }\,}}_{\mathbb {R}}K_G\big (\tau -n^3, \lambda - n^3, \Phi ({\overline{n}}_{123})\big ) {{\,\mathrm{\int }\,}}_{\lambda = \tau _1 + \tau _2 + \tau _3} \\&\qquad \prod _{j=1}^3 \widehat{u}_j (\tau _j, n_j) \, d\tau _1 \, d\tau _2 \,d\lambda , \end{aligned}$$

where the kernel $K_G$ is given by the following expression

3.4 $$\begin{aligned} K_G (\tau , \lambda , \Phi ) = {{\,\mathrm{\int }\,}}_{\mathbb {R}}\bigg (\widehat{\varphi }(\tau -\mu ) \widehat{\varphi }(\mu - \lambda ) \frac{1}{\Phi } {\mathcal {H}}\widehat{\eta }\Big (\frac{\mu }{\Phi }\Big ) + \widehat{\varphi }(\tau - \mu ) {\mathcal {H}}\widehat{\varphi } (\mu - \lambda ) \frac{1}{\Phi } \widehat{\eta } \Big (\frac{\mu }{\Phi }\Big ) \bigg )\, d\mu , \end{aligned}$$

and satisfies the following estimates

3.5 $$\begin{aligned} | K_G (\tau , \lambda , \Phi ) |&\lesssim \frac{1}{\langle \tau -\lambda \rangle ^\alpha } \min \bigg (\frac{1}{\langle \Phi \rangle }, \frac{1}{\langle \lambda \rangle }\bigg ) + \frac{1}{\langle \tau -\lambda \rangle } \min \bigg ( \frac{1}{\langle \Phi \rangle }, \frac{1}{\langle \tau \rangle } \bigg ), \nonumber \\&\lesssim \frac{1}{\langle \tau -\lambda \rangle } \min \bigg ( \frac{1}{\langle \Phi \rangle }, \frac{1}{\langle \tau \rangle } \bigg ), \end{aligned}$$

where $\alpha$ is a large enough positive number and $|\Phi |\ge 1$.

### Proof

Since the relation between $|n_2|$ and $|n_3|$ will not play an important role in the proof, we will use ${\mathbf {G}}_{*}$ to denote both ${\mathbf {G}}_{*, \ge },{\mathbf {G}}_{*,>}$. We want to calculate the following

$$\begin{aligned}&{{\mathcal {F}}}_{t,x} \big ( {\mathbf {G}}_*(u_1, u_2,u_3) \big ) (\tau , n) \\&\quad = \sum _{\begin{array}{c} n=n_1+n_2+n_3,\\ {\overline{n}}_{123} \in {\mathbb {X}}_*(n) \end{array}} in_1 {{\,\mathrm{\int }\,}}_{\mathbb {R}}\widehat{\varphi } (\tau -\mu ) {{\mathcal {F}}}_t \bigg ( \int _0^t e^{i(t-t')n^3} \eta \big ( \Phi ({\overline{n}}_{123})(t-t')\big ) F(t') \, dt' \bigg ) (\mu ) \, d\mu , \end{aligned}$$

where $F(t) = \varphi (t) \prod _{j=1}^3 \widehat{u}_j(t, n_j)$. Note that $|\Phi ({\overline{n}}_{123})| \ge 1$ for ${\overline{n}}_{123}\in {\mathbb {X}}_*(n)$ and denote it by $\Phi$, for simplicity. Proceeding as in the proof of Proposition 3.1, we have

$$\begin{aligned}&{{\mathcal {F}}}_{t,x} \big ({\mathbf {G}}_* (u_1, u_2,u_3) \big )(\tau , n) \\&= \sum _{\begin{array}{c} n=n_1+n_2+n_3,\\ {\overline{n}}_{123} \in {\mathbb {X}}_*(n) \end{array}} n_1 {{\,\mathrm{\int }\,}}_{\mathbb {R}}\widehat{\varphi }(\tau -\mu ) \bigg [ \widehat{F}(\mu ) \frac{1}{\Phi } {\mathcal {H}}\widehat{\eta } \bigg (\frac{\mu -n^3}{\Phi }\bigg ) + {\mathcal {H}}\widehat{F}(\mu ) \frac{1}{\Phi } \widehat{\eta } \bigg (\frac{\mu -n^3}{\Phi } \bigg ) \bigg ] d\mu \end{aligned}$$

Substituting $\widehat{F}(\mu )$ and ${\mathcal {H}}\widehat{F}(\mu )$, we obtain the intended expression. It remains to show the kernel estimate. First, note that for a Schwartz function *f*, we have

$$\begin{aligned} |{\mathcal {H}}f(\xi )|&\le \lim _{\varepsilon \rightarrow 0} {{\,\mathrm{\int }\,}}_{\varepsilon<|\mu |<1} \bigg | \frac{f(\xi -\mu ) - f(\xi )}{\mu } \bigg | d \mu \\&\quad + \bigg | \lim _{\varepsilon \rightarrow 0} {{\,\mathrm{\int }\,}}_{\varepsilon<|\mu | <1} \frac{f(\xi )}{\mu } d\mu \bigg | + {{\,\mathrm{\int }\,}}_{|\mu | \ge 1} \bigg | \frac{f(\xi -\mu )}{\mu } \Bigg | d\mu . \end{aligned}$$

Using mean value theorem and distinguishing the cases $|\xi |\lesssim 1$ and $|\xi |\gg 1$, we can estimate the first term by $\langle \xi \rangle ^{-\alpha }$ for any $\alpha >0$. The second contribution is equal to zero. Lastly, using Lemma 2.8, the third term is controlled by $\langle \xi \rangle ^{-1}$. Consequently,

3.6 $$\begin{aligned} |{\mathcal {H}}f(\xi )| \lesssim \frac{1}{\langle \xi \rangle }. \end{aligned}$$

Since $\widehat{\varphi }$ is a Schwartz function, using (3.6),

$$\begin{aligned} \frac{1}{\langle \Phi \rangle }\bigg | {\mathcal {H}}\widehat{\eta } \bigg (\frac{\mu }{\Phi }\bigg )\bigg | \lesssim \frac{1}{\langle \Phi \rangle } \bigg (\mathbb {1}_{|\mu | \le |\Phi |} + \mathbb {1}_{|\mu | \ge |\Phi |\ge 1} \frac{\langle \Phi \rangle }{\langle \mu \rangle } \bigg ) \lesssim \min \bigg (\frac{1}{\langle \Phi \rangle }, \frac{1}{\langle \mu \rangle } \bigg ). \end{aligned}$$

Now, considering the kernel and the estimates for ${\mathcal {H}}\widehat{\eta }, {\mathcal {H}}\widehat{\varphi }$, we have the following

$$\begin{aligned} |K_G(\tau , \lambda , \Phi )|&\lesssim {{\,\mathrm{\int }\,}}_{\mathbb {R}}|\widehat{\varphi }(\tau -\mu ) \widehat{\varphi }(\mu - \lambda )| \min \bigg (\frac{1}{\langle \Phi \rangle }, \frac{1}{\langle \mu \rangle } \bigg ) d\mu \\&\quad + {{\,\mathrm{\int }\,}}_{\mathbb {R}}\frac{1}{\langle \Phi \rangle \langle \mu -\lambda \rangle } \bigg |\widehat{\varphi }(\tau -\mu ) \widehat{\eta }\bigg (\frac{\mu }{\Phi }\bigg )\bigg | d\mu =: \text {I}+ \text {I I} . \end{aligned}$$

Applying Lemma 2.8 the $\text {I}$, we have

$$\begin{aligned} \text {I}\lesssim {{\,\mathrm{\int }\,}}_{\mathbb {R}}\frac{1}{\langle \tau -\mu \rangle ^{\alpha +1} \langle \mu -\lambda \rangle ^{\alpha +1}} \min \bigg (\frac{1}{\langle \Phi \rangle }, \frac{1}{\langle \mu \rangle } \bigg ) d\mu \lesssim \frac{1}{\langle \tau -\lambda \rangle ^\alpha } \min \bigg ( \frac{1}{\langle \Phi \rangle }, \frac{1}{\langle \lambda \rangle }\bigg ). \end{aligned}$$

For the second contribution $\text {I I}$, applying Lemma 2.8 or Cauchy–Schwarz inequality give the following estimates

$$\begin{aligned} \text {I I}&\lesssim {{\,\mathrm{\int }\,}}_{\mathbb {R}}\frac{1}{\langle \Phi \rangle \langle \mu -\lambda \rangle \langle \tau -\mu \rangle ^{1+}} d\mu \lesssim \frac{1}{\langle \Phi \rangle \langle \tau -\lambda \rangle },\\ \text {I I}&\lesssim \bigg ({{\,\mathrm{\int }\,}}_{\mathbb {R}}\frac{d\mu }{\langle \tau -\mu \rangle ^{2} \langle \mu \rangle ^{2}} \bigg )^\frac{1}{2} \bigg ({{\,\mathrm{\int }\,}}_{\mathbb {R}}\frac{d\mu }{\langle \tau -\mu \rangle ^{2} \langle \mu -\lambda \rangle ^{2}} \bigg )^\frac{1}{2} \lesssim \frac{1}{\langle \tau \rangle \langle \tau - \lambda \rangle }. \end{aligned}$$

Consequently, $\text {I I} \lesssim \frac{1}{\langle \tau -\lambda \rangle } \min \bigg (\frac{1}{\langle \Phi \rangle }, \frac{1}{\langle \tau \rangle } \bigg )$. For (3.5), we consider different cases $\max (\langle \Phi \rangle , \langle \lambda \rangle ) \gtrsim \max (\langle \Phi \rangle , \langle \tau \rangle )$ or $\max (\langle \Phi \rangle , \langle \lambda \rangle ) \ll \max (\langle \Phi \rangle , \langle \tau \rangle )$. Note that for the latter, $\max (\langle \Phi \rangle , \langle \tau \rangle ) = \langle \tau \rangle$ and $\langle \tau -\lambda \rangle \sim \langle \tau \rangle$. The estimate follows by choosing $\alpha \ge 2$.

$$\square $$

### Remark 3.3

For $*\in \{A,B\}$, consider the operators ${\mathcal {D}}\mathcal {NR}_{*, \ge }(u_1,u_2,u_3)$ and ${\mathbf {G}}_{*,\ge }(u_1,u_2,u_3)$, and the kernel estimates in Propositions 3.1 and 3.2. Then,

$$\begin{aligned} \big | {{\mathcal {F}}}_{t,x} \big ({\mathcal {D}}\mathcal {NR}_{*, \ge } (u_1,u_2,u_3)\big ) (\tau ,n) \big |&\lesssim \sum _{\begin{array}{c} n=n_1+n_2+n_3, \\ {\overline{n}}_{123}\in {\mathbb {X}}_*(n), \\ |n_2| \ge |n_3| \end{array}} {{\,\mathrm{\int }\,}}_{\mathbb {R}}\frac{|n_1|}{\langle \tau -\lambda \rangle \langle \tau -n^3 \rangle } \widehat{F}(\lambda ,{\overline{n}}_{123}) \, d\lambda , \\ \big | {{\mathcal {F}}}_{t,x} \big ({\mathbf {G}}_{*, \ge } (u_1,u_2,u_3) \big ) (\tau ,n) \big |&\lesssim \sum _{\begin{array}{c} n=n_1+n_2+n_3 ,\\ {\overline{n}}_{123}\in {\mathbb {X}}_*(n), \\ |n_2| \ge |n_3| \end{array}} {{\,\mathrm{\int }\,}}_{\mathbb {R}}\frac{|n_1|}{\langle \tau -\lambda \rangle } \min \bigg ( \frac{1}{\langle \Phi ({\overline{n}}_{123}) \rangle }, \frac{1}{\langle \tau -n^3 \rangle } \bigg ) \\&\quad \times \widehat{F}(\lambda ,{\overline{n}}_{123}) \, d\lambda , \end{aligned}$$

where $\widehat{F}(\lambda ,{\overline{n}}_{123}) = \big (|\widehat{u}_1(\cdot ,n_1)| *|\widehat{u}_2(\cdot ,n_2)| *|\widehat{u}_3(\cdot ,n_3)| \big )(\lambda )$. Thus, for the modified Duhamel operators ${\mathbf {G}}_{*, \ge }$ we can ‘exchange’ the smoothing in time through $\langle \tau -n^3 \rangle$ for smoothing in space through $\langle \Phi ({\overline{n}}_{123}) \rangle$, unlike the usual Duhamel operator.

### Proposition 3.4

Let $*\in \{A,B\}$. Then, the convolution operators ${\mathbf {B}}_{*, \ge }, {\mathbf {B}}_{*,>}$ have the following space-time Fourier transform

$$\begin{aligned}&{{\mathcal {F}}}_{t,x} \big ( {\mathbf {B}}_{*, \underset{(>)}{\ge }} (u_1,u_2,u_3) \big )(\tau ,n) \\&\quad = \sum _{\begin{array}{c} n=n_1+n_2+n_3, \\ {\overline{n}}_{123}\in {\mathbb {X}}_*(n), \\ |n_2| \underset{(>)}{\ge } |n_3| \end{array} } n_1 {{\,\mathrm{\int }\,}}_{\mathbb {R}}K_B(\tau -n^3, \lambda - n^3, \Phi ({\overline{n}}_{123})) {{\,\mathrm{\int }\,}}_{\lambda = \tau _1 + \tau _2 + \tau _3}\\&\qquad \prod _{j=1}^3 \widehat{u}_j (\tau _j, n_j) \, d\tau _1 \, d\tau _2 \, d\lambda , \end{aligned}$$

where the kernel $K_B$ is given by

$$\begin{aligned}&K_B (\tau , \lambda , \Phi ) = {{\,\mathrm{\int }\,}}_{\mathbb {R}}\frac{\widehat{\varphi }(\tau - \mu ) - \widehat{\varphi }(\tau )}{\mu } \widehat{\varphi }(\mu -\lambda ) \, d\mu - {{\,\mathrm{\int }\,}}_{\mathbb {R}}\widehat{\varphi }(\tau -\mu ) \widehat{\varphi }(\mu -\lambda ) \frac{1}{\Phi }{\mathcal {H}}\widehat{\eta }\Big (\frac{\mu }{\Phi }\Big )d\mu \\&\quad + {{\,\mathrm{\int }\,}}_{\mathbb {R}}\widehat{\varphi }(\tau - \mu ) {\mathcal {H}}\widehat{\varphi }(\mu -\lambda ) \frac{1}{\Phi }\widehat{\eta }\bigg (\frac{\mu }{\Phi }\bigg ) \, d\mu , \end{aligned}$$

and satisfies the following estimate

$$\begin{aligned} | K_B (\tau , \lambda , \Phi ) | \lesssim \frac{1}{\langle \tau \rangle ^\alpha \langle \lambda \rangle } + \frac{\langle \lambda + \Phi \rangle }{\langle \tau -\lambda \rangle ^\alpha \langle \lambda \rangle } \min \bigg ( \frac{1}{\langle \Phi \rangle }, \frac{1}{\langle \lambda \rangle }\bigg ) + \frac{\langle \tau +\Phi \rangle }{\langle \tau -\lambda \rangle } \min \bigg ( \frac{1}{\langle \Phi \rangle }, \frac{1}{\langle \tau \rangle } \bigg )^2, \end{aligned}$$

for any $\alpha >0$ and $|\Phi |\ge 1$.

### Proof

Let $*\in \{A,B\}$ and let ${\mathbf {B}}_*$ denote both ${\mathbf {B}}_{*, \ge }$ and ${\mathbf {B}}_{*,>}$. By definition, we have that ${\mathbf {B}}_* = {\mathcal {D}}\mathcal {NR}_* - {\mathbf {G}}_*$. From Propositions 3.1 and 3.2, the kernel $K_B$ defined as $K_B(\tau , \lambda , \Phi ) = -i K(\tau , \lambda ) - K_G(\tau , \lambda , \Phi )$ giving the intended formula for the kernel $K_B$. Using the expressions (3.1) and (3.4) for the kernels, we can rewrite $K_B(\tau , \lambda , \Phi )$ as

$$\begin{aligned}&{{\,\mathrm{\int }\,}}_{|\mu | \le 1} \big (\widehat{\varphi }(\tau -\mu ) - \widehat{\varphi }(\tau ) \big ) \widehat{\varphi }(\mu -\lambda ) \frac{1}{\mu }\, d\mu - {{\,\mathrm{\int }\,}}_{|\mu |\le 1} \widehat{\varphi }(\tau - \mu ) \widehat{\varphi }(\mu -\lambda ) \frac{1}{\Phi }{\mathcal {H}}\widehat{\eta }\bigg (\frac{\mu }{\Phi }\bigg ) \, d\mu \\&\qquad + {{\,\mathrm{\int }\,}}_{|\mu |>1} \widehat{\varphi }(\tau - \mu ) \widehat{\varphi }(\mu -\lambda ) \bigg \{ \frac{1}{\mu }- \frac{1}{\Phi }{\mathcal {H}}\widehat{\eta }\bigg (\frac{\mu }{\Phi }\bigg ) \bigg \}\, d\mu \\&\qquad + {{\,\mathrm{\int }\,}}_{\mathbb {R}}\widehat{\varphi }(\tau - \mu ) {\mathcal {H}}\widehat{\varphi }(\mu -\lambda ) \frac{1}{\Phi }\widehat{\eta }\bigg (\frac{\mu }{\Phi }\bigg ) \, d\mu \\&\qquad - {{\,\mathrm{\int }\,}}_{|\mu |>1} \widehat{\varphi }(\tau ) \widehat{\varphi }(\mu -\lambda ) \frac{1}{\mu }\, d\mu \\&\quad =: \text {I}_1 + \text {I}_2 + \text {I}_3 + \text {I}_4 + \text {I}_5. \end{aligned}$$

For $\text {I}_1$, using mean value theorem gives, for any $\alpha >0$,

$$\begin{aligned} |\text {I}_1|&\lesssim {{\,\mathrm{\int }\,}}_{|\mu | \le 1} \mathbb {1}_{|\tau | \lesssim 1} \frac{d\mu }{\langle \tau \rangle ^\alpha \langle \mu \rangle ^\alpha \langle \mu -\lambda \rangle ^{1+\alpha }} \\&\, + {{\,\mathrm{\int }\,}}_{|\mu | \le 1} \mathbb {1}_{|\tau | \gg 1} \frac{d\mu }{\langle \tau \rangle ^\alpha \langle \mu \rangle ^\alpha \langle \mu -\lambda \rangle ^{1+\alpha }} \lesssim \frac{1}{\langle \tau \rangle ^\alpha \langle \lambda \rangle ^\alpha }. \end{aligned}$$

Using (3.6) and Cauchy–Schwarz inequality gives

$$\begin{aligned} |\text {I}_2|&\lesssim {{\,\mathrm{\int }\,}}_{|\mu |\le 1} \frac{d\mu }{\langle \Phi \rangle \langle \tau -\mu \rangle ^{\alpha } \langle \mu -\lambda \rangle ^{\alpha +1} \langle \mu \rangle ^{\alpha }} \lesssim \frac{1}{\langle \Phi \rangle \langle \tau -\lambda \rangle ^\alpha \langle \lambda \rangle ^\alpha }. \end{aligned}$$

Before estimating $\text {I}_3$, note that since ${\mathcal {H}}\widehat{\eta }(-1) = -1$ and using mean value theorem, we get

$$\begin{aligned} \bigg | \frac{1}{\mu }- \frac{1}{\Phi }{\mathcal {H}}\widehat{\eta }\bigg (\frac{\mu }{\Phi }\bigg ) \bigg |&\sim \frac{1}{\langle \mu \rangle } \bigg | {\mathcal {H}}\widehat{\eta }(-1) - \frac{\mu }{\Phi } {\mathcal {H}}\widehat{\eta } \bigg (\frac{\mu }{\Phi }\bigg )\bigg | \\&\lesssim \mathbb {1}_{\langle \Phi \rangle \gtrsim \langle \mu \rangle } \frac{1}{\langle \mu \rangle } \bigg | -1 - \frac{\mu }{\Phi }\bigg | + \mathbb {1}_{\langle \Phi \rangle \ll \langle \mu \rangle } \frac{\langle \mu +\Phi \rangle }{\langle \mu \rangle ^2} \\&\lesssim \frac{\langle \mu +\Phi \rangle }{\langle \mu \rangle } \min \bigg ( \frac{1}{\langle \Phi \rangle }, \frac{1}{\langle \mu \rangle } \bigg ). \end{aligned}$$

Using the above estimate, it follows from previous arguments that, for any $\alpha >0$,

$$\begin{aligned} |\text {I}_3|&\lesssim \mathbb {1}_{\langle \Phi \rangle \gtrsim \langle \tau \rangle } \frac{\langle \lambda + \Phi \rangle }{\langle \tau -\lambda \rangle ^\alpha \langle \lambda \rangle \langle \Phi \rangle }+ \mathbb {1}_{\langle \Phi \rangle \ll \langle \tau \rangle } \frac{\langle \lambda +\Phi \rangle }{\langle \tau -\lambda \rangle ^\alpha \langle \lambda \rangle ^2} \lesssim \frac{\langle \lambda + \Phi \rangle }{\langle \tau -\lambda \rangle ^\alpha \langle \lambda \rangle } \min \bigg ( \frac{1}{\langle \Phi \rangle }, \frac{1}{\langle \lambda \rangle }\bigg ). \end{aligned}$$

In order to estimate $\text {I}_4$, we start by showing a bound for $\frac{1}{\Phi } \widehat{\eta } \big (\frac{\mu }{\Phi }\big )$. If $|\Phi | \gtrsim |\mu |$, we use the fact that $\widehat{\eta }(-1) =0$ and mean value theorem. Otherwise, $|\Phi | \ll |\mu |$ and $\langle \mu - \Phi \rangle \sim \langle \mu \rangle$. It follows that

$$\begin{aligned} \bigg | \frac{1}{\Phi } \widehat{\eta } \bigg (\frac{\mu }{\Phi }\bigg ) \bigg |&\lesssim \mathbb {1}_{|\Phi | \gtrsim |\mu |} \frac{1}{\langle \Phi \rangle } \bigg | \widehat{\eta }\bigg (\frac{\mu }{\Phi }\bigg ) - \widehat{\eta }(-1) \bigg | + \mathbb {1}_{|\Phi | \ll |\mu |} \frac{1}{\langle \mu \rangle } \bigg | \frac{\mu }{\Phi } \widehat{\eta } \bigg (\frac{\mu }{\Phi } \bigg ) \bigg | \\&\lesssim \langle \mu +\Phi \rangle \min \bigg ( \frac{1}{\langle \Phi \rangle }, \frac{1}{\langle \mu \rangle }\bigg )^2. \end{aligned}$$

Using the above estimate and the fact that $|{\mathcal {H}}\widehat{\varphi }(\mu -\lambda )| \lesssim \langle \mu -\lambda \rangle ^{-1}$ in (3.6), we have

$$\begin{aligned} |\text {I}_4|&\lesssim \mathbb {1}_{\langle \Phi \rangle \gtrsim \langle \tau \rangle } \frac{\langle \tau + \Phi \rangle }{\langle \Phi \rangle ^2 \langle \tau -\lambda \rangle } + \mathbb {1}_{\langle \Phi \rangle \ll \langle \tau \rangle } \frac{\langle \tau +\Phi \rangle }{\langle \tau -\lambda \rangle \langle \tau \rangle ^2} \lesssim \frac{\langle \tau +\Phi \rangle }{\langle \tau -\lambda \rangle } \min \bigg ( \frac{1}{\langle \Phi \rangle }, \frac{1}{\langle \tau \rangle } \bigg )^2. \end{aligned}$$

For the last contribution, for any $\alpha >0$, we have

$$\begin{aligned} |\text {I}_5|&\lesssim {{\,\mathrm{\int }\,}}_{|\mu |>1} \frac{\langle \tau \rangle ^{\alpha } |\widehat{\varphi }(\tau )|\langle \mu -\lambda \rangle | \widehat{\varphi }(\mu -\lambda )|}{ \langle \tau \rangle ^{\alpha } \langle \mu -\lambda \rangle \langle \mu \rangle } \ d\mu \lesssim \frac{1}{\langle \tau \rangle ^\alpha \langle \lambda \rangle }. \end{aligned}$$

$$\square $$

We want to further split the operators ${\mathbf {B}}_{*,\ge }, {\mathbf {B}}_{*,>}$, $*\in \{A,B\}$, to obtain better kernel estimates. We will split the kernel $K_B$ in two pieces: when we can estimate the multiplier directly, and when we also have to use $\sigma _{\max } = \max _{j=1,2,3} |\tau _j - n_j^3|$. Let $K_B = K_{0} + K_{+}$ where the kernels are defined below

$$\begin{aligned}&K_{0}(\tau , \lambda , \Phi ) \\&\quad = \mathbb {1}_{\langle \lambda \rangle \gtrsim \langle \Phi \rangle } \bigg ( {{\,\mathrm{\int }\,}}_{|\mu | \le 1} \big (\widehat{\varphi }(\tau -\mu ) - \widehat{\varphi }(\tau ) \big ) \widehat{\varphi }(\mu -\lambda ) \frac{1}{\mu }\ d\mu - {{\,\mathrm{\int }\,}}_{|\mu |>1} \widehat{\varphi }(\tau ) \widehat{\varphi }(\mu -\lambda ) \frac{1}{\mu }\ d\mu \bigg ) \\&\qquad \, + \mathbb {1}_{\langle \lambda +\Phi \rangle \lesssim \langle \tau -\lambda \rangle } \bigg ({{\,\mathrm{\int }\,}}_{|\mu |>1} \widehat{\varphi }(\tau - \mu ) \widehat{\varphi }(\mu -\lambda ) \bigg \{ \frac{1}{\mu }- \frac{1}{\Phi }{\mathcal {H}}\widehat{\eta }\bigg (\frac{\mu }{\Phi }\bigg ) \bigg \}\, d\mu \bigg )\\&\qquad \, + \mathbb {1}_{\langle \tau +\Phi \rangle \lesssim \langle \tau -\lambda \rangle } \bigg ({{\,\mathrm{\int }\,}}_{\mathbb {R}}\widehat{\varphi }(\tau - \mu ) {\mathcal {H}}\widehat{\varphi }(\mu -\lambda ) \frac{1}{\Phi }\widehat{\eta }\bigg (\frac{\mu }{\Phi }\bigg ) \, d\mu \bigg ) \\&\quad \, - {{\,\mathrm{\int }\,}}_{|\mu | \le 1} \widehat{\varphi }(\tau - \mu ) \widehat{\varphi }(\mu -\lambda ) \frac{1}{\Phi } {\mathcal {H}}\widehat{\eta }\bigg ( \frac{\mu }{\Phi }\bigg ) \, d \mu ,\\&K_{+} (\tau , \lambda , \Phi )\\&\quad = \mathbb {1}_{\langle \lambda \rangle \ll \langle \Phi \rangle } \bigg ( {{\,\mathrm{\int }\,}}_{|\mu | \le 1} \big (\widehat{\varphi }(\tau -\mu ) - \widehat{\varphi }(\tau ) \big ) \widehat{\varphi }(\mu -\lambda ) \frac{1}{\mu }\ d\mu - {{\,\mathrm{\int }\,}}_{|\mu |>1} \widehat{\varphi }(\tau ) \widehat{\varphi }(\mu -\lambda ) \frac{1}{\mu }\ d\mu \bigg ) \\&\qquad \, + \mathbb {1}_{\langle \lambda + \Phi \rangle \gg \langle \tau -\lambda \rangle }{{\,\mathrm{\int }\,}}_{|\mu |>1} \widehat{\varphi }(\tau - \mu ) \widehat{\varphi }(\mu -\lambda ) \bigg \{ \frac{1}{\mu }- \frac{1}{\Phi }{\mathcal {H}}\widehat{\eta }\bigg (\frac{\mu }{\Phi }\bigg ) \bigg \}\, d\mu \\&\qquad \,+ \mathbb {1}_{\langle \tau + \Phi \rangle \gg \langle \tau -\lambda \rangle } \bigg ({{\,\mathrm{\int }\,}}_{\mathbb {R}}\widehat{\varphi }(\tau - \mu ) {\mathcal {H}}\widehat{\varphi }(\mu -\lambda ) \frac{1}{\Phi }\widehat{\eta }\bigg (\frac{\mu }{\Phi }\bigg ) \, d\mu \bigg ). \end{aligned}$$

Thus, we have the following estimates for the kernels, for any $0\le \alpha \le 1$,

3.7 $$\begin{aligned} |K_{0}(\tau , \lambda , \Phi )|&\lesssim \frac{1}{\langle \tau \rangle ^{1+\alpha } \langle \Phi \rangle ^{1-\alpha }} , \end{aligned}$$

3.8 $$\begin{aligned} |K_{+}(\tau , \lambda , \Phi )|&\lesssim \mathbb {1}_{\langle \lambda \rangle \ll \langle \Phi \rangle } \frac{1}{\langle \tau -\lambda \rangle \langle \tau \rangle } + \frac{\langle \lambda +\Phi \rangle ^{1-\alpha }}{\langle \tau -\lambda \rangle \langle \tau \rangle } \min \bigg ( \frac{1}{\langle \Phi \rangle } , \frac{1}{\langle \tau \rangle } \bigg )^{1-\alpha }. \end{aligned}$$

In Sect. 6.1, we will see that the contribution corresponding to the kernel $K_0$ in ${\mathbf {B}}_{*, \ge }, {\mathbf {B}}_{*, >}$ can be easily estimated, due to the explicit smoothing in space. However, in order to estimate the one corresponding to $K_+$, we will have to use the largest modulation. In particular, from (3.8), we see that

3.9 $$\begin{aligned} \big |K_{+}\big (\tau -n^3, \lambda - n^3, \Phi ({\overline{n}}_{123})\big ) \big | \lesssim \frac{\langle \sigma _{\max } \rangle ^{1-\alpha }}{\langle \tau -\lambda \rangle \langle \tau -n^3 \rangle \langle \Phi ({\overline{n}}_{123}) \rangle ^{1-\alpha }}, \end{aligned}$$

for any $0\le \alpha \le 1$, since $\lambda = \tau _1 + \tau _2 + \tau _3$ and

$$\begin{aligned} |\lambda - n^3 + \Phi ({\overline{n}}_{123})| = |\tau _1 - n_1^3 + \tau _2 - n_2^3 + \tau _3 - n_3^3| \lesssim \max \limits _{j=1,2,3} |\tau _j - n_j^3| = \sigma _{\max }. \end{aligned}$$

Thus, we can use $\sigma _{\max }$ in order to estimate the numerator of the second contribution in (3.8), which motivates splitting the operators depending on which modulation is the largest. In particular, we have

3.10 $$\begin{aligned} {\mathbf {B}}_{*, \ge }&= {\mathbf {B}}^{0}_{*,\ge } + {\mathbf {B}}^{1}_{*,\ge } + {\mathbf {B}}^{2}_{*,\ge } + {\mathbf {B}}^{3}_{*, \ge }, \end{aligned}$$

where ${\mathbf {B}}^{0}_{*,\ge }$ has kernel $K_{0}$ and ${\mathbf {B}}^{j}_{*,\ge }$ has kernel $K_{+}$ localized to the region where ${\sigma _{\max } = |\sigma _j|}$, $j=1,2,3$. An analogous decomposition holds for ${\mathbf {B}}_{*,>}$.

## System of Equations

Instead of running a contraction mapping argument on the integral equation (2.6), we will solve an ordered system of equations. In this section, we establish the relevant equations for *u* and *w* and the main results needed to show Theorem 1.3. For a fixed *p* in $2\le p <\infty$, we will only focus on showing local well-posedness in ${\mathcal {F}}L^{\frac{1}{2},p}({\mathbb {T}})$. The result for $s>\frac{1}{2}$ follows from a persistence of regularity argument. See Remark 4.4 for more details.

For a fixed $w\in Z_0$, we consider the following equation for *u*

4.1 $$\begin{aligned} u = w + \varphi _T \big [ {\mathbf {G}}_{A, \ge }(w,\overline{u},u) + {\mathbf {G}}_{A,>}(w,u,\overline{u}) + {\mathbf {G}}_{B, \ge }(w,\overline{w},u) + {\mathbf {G}}_{B, >}(w,w, \overline{u}) \big ]. \nonumber \\ \end{aligned}$$

We first solve the equation (4.1) obtaining $u=u[w]$, i.e., *u* parameterized by *w*.

### Proposition 4.1

For any $w\in Z_0$ satisfying $\Vert w\Vert _{Z_0} \le A_2$, there exists a unique $u\in Y_0$ with $\Vert u\Vert _{Y_0} \le A_3$ satisfying (4.1), for some $T=T(A_2)>0$. The mapping $w \mapsto u[w]$ is Lipschitz from the $A_2$-ball of $Z_0$ to the $A_3$-ball of $Y_0$.

To guarantee that $u=u[w]$, the solution of (4.1), satisfies the Duhamel formulation (2.6), then *w* must satisfy the following equation

4.2 $$\begin{aligned} \begin{aligned} w&= \varphi (t) S(t) u_0 + \varphi _T \cdot \mathcal {IR}(u, u, u) \\&\quad + \varphi _T \cdot {\mathcal {D}}\mathcal {NR}_{C, \ge }(u,\overline{u}, u) + \varphi _T \cdot {\mathcal {D}}\mathcal {NR}_{C,>}(u,u, \overline{u}) \\&\quad + \varphi _T \cdot {\mathcal {D}}\mathcal {NR}_{D, \ge }(u,\overline{u},u) + \varphi _T \cdot {\mathcal {D}}\mathcal {NR}_{D,>}(u,u, \overline{u})\\&\quad + \varphi _T\big [ {\mathbf {B}}_{A, \ge }(w,\overline{u},u) + {\mathbf {B}}_{A,>}(w,u, \overline{u}) + {\mathbf {B}}_{B, \ge }(w,\overline{w}, u) + {\mathbf {B}}_{B,>}(w,w,\overline{u}) \big ] \\&\quad + \varphi _T \big [ {\mathcal {D}}\mathcal {NR}_{A, \ge } (u,\overline{u},u) - {\mathcal {D}}\mathcal {NR}_{A, \ge } (w,\overline{u},u) \big ] \\&\quad + \varphi _T \big [ {\mathcal {D}}\mathcal {NR}_{A,>} (u,u,\overline{u}) - {\mathcal {D}}\mathcal {NR}_{A,>} (w,u,\overline{u}) \big ] \\&\quad + \varphi _T \big [ {\mathcal {D}}\mathcal {NR}_{B, \ge } (u,\overline{u}, u) - {\mathcal {D}}\mathcal {NR}_{B, \ge } (w,\overline{w}, u) \big ] \\&\quad + \varphi _T \big [ {\mathcal {D}}\mathcal {NR}_{B,>} (u,u, \overline{u}) - {\mathcal {D}}\mathcal {NR}_{B, >} (w,w, \overline{u}) \big ] . \end{aligned} \end{aligned}$$

In order to solve the above equation, we use a *partial* second iteration by replacing $u=u[w]$ by its equation (4.1). The decomposition on the operators ${\mathcal {D}}\mathcal {NR}$ and ${\mathbf {B}}$, introduced in Sects. 2 and 3, explicitly identifies which entries have the largest frequencies and the largest modulations, respectively. This information will guide the second iteration process.

For the terms ${\mathcal {D}}\mathcal {NR}_{*, \ge }, {\mathcal {D}}\mathcal {NR}_{*,>}$, $*\in \{A,B,C,D\}$, we replace the equation for *u* (4.1) from left to right to obtain only cubic and quintic terms, as in the following example

$$\begin{aligned}&{\mathcal {D}}\mathcal {NR}_{C,\ge }(u,\overline{u}, u) = {\mathcal {D}}\mathcal {NR}_{C, \ge } (w,\overline{u}, u) \\&\quad + {\mathcal {D}}\mathcal {NR}_{C, \ge } \big ( \varphi _T \cdot {\mathbf {G}}_{A,\ge } [w,\overline{u}, u] , \overline{u}, u\big ) + {\mathcal {D}}\mathcal {NR}_{C, \ge } \big ( \varphi _T \cdot {\mathbf {G}}_{A,>} [w,u,\overline{u}] , \overline{u}, u\big ) \\&\quad + {\mathcal {D}}\mathcal {NR}_{C, \ge } \big ( \varphi _T \cdot {\mathbf {G}}_{B,\ge } [w,\overline{w}, u] , \overline{u}, u\big ) + {\mathcal {D}}\mathcal {NR}_{C, \ge } \big ( \varphi _T \cdot {\mathbf {G}}_{B,>} [w,w, \overline{u}] , \overline{u}, u\big ), \\&{\mathcal {D}}\mathcal {NR}_{C, \ge } (w,\overline{u}, u) ={\mathcal {D}}\mathcal {NR}_{C, \ge } (w,\overline{w}, u) \\&\quad + {\mathcal {D}}\mathcal {NR}_{C, \ge } \big ( w, \overline{\varphi _T \cdot {\mathbf {G}}_{A,\ge } [w,\overline{u}, u] },u\big ) + {\mathcal {D}}\mathcal {NR}_{C, \ge } \big ( w, \overline{\varphi _T \cdot {\mathbf {G}}_{A,>} [w,u,\overline{u}] }, u\big ) \\&\quad + {\mathcal {D}}\mathcal {NR}_{C, \ge } \big ( w, \overline{\varphi _T \cdot {\mathbf {G}}_{B,\ge } [w,\overline{w}, u]} , u\big ) + {\mathcal {D}}\mathcal {NR}_{C, \ge } \big ( w, \overline{\varphi _T \cdot {\mathbf {G}}_{B,>} [w,w, \overline{u}] }, u\big ),\\&{\mathcal {D}}\mathcal {NR}_{C, \ge } (w,\overline{w}, u) = {\mathcal {D}}\mathcal {NR}_{C, \ge } (w,\overline{w}, w) \\&\quad + {\mathcal {D}}\mathcal {NR}_{C, \ge } \big ( w, \overline{w}, \varphi _T \cdot {\mathbf {G}}_{A,\ge } [w,\overline{u}, u] \big ) + {\mathcal {D}}\mathcal {NR}_{C, \ge } \big ( w, \overline{w}, \varphi _T \cdot {\mathbf {G}}_{A,>} [w,u, \overline{u}] \big ) \\&\quad + {\mathcal {D}}\mathcal {NR}_{C, \ge } \big ( w, \overline{w}, \varphi _T \cdot {\mathbf {G}}_{B,\ge } [w,\overline{w}, u] \big ) + {\mathcal {D}}\mathcal {NR}_{C, \ge } \big ( w, \overline{w}, \varphi _T \cdot {\mathbf {G}}_{B,>} [w,w, \overline{u}] \big ). \end{aligned}$$

This strategy prioritizes the entry with the derivative followed by the one with the largest frequency between the remaining two factors. For ${\mathcal {D}}\mathcal {NR}_{*,\ge }, {\mathcal {D}}\mathcal {NR}_{*, >}$ with $*\in \{A,B\}$, there will be no cubic terms after second iteration, due to the differences in (4.2).

For the terms ${\mathbf {B}}_{*,\ge }, {\mathbf {B}}_{*, >}$, with $*\in \{A,B\}$, we split the operators into four pieces ${\mathbf {B}}^{j}_{*,\ge }, {\mathbf {B}}^{j}_{*,>}$, $j=0,1,2,3$, as defined in (3.10). The contributions corresponding to $j=0$ are easily estimated, but for $j=1,2,3$ the largest modulation plays an important role in estimating the kernel. If the *j*th entry corresponds to a *u* or $\overline{u}$ term, we replace it with the equation for *u* (4.1). For example, we have

$$\begin{aligned} {\mathbf {B}}_{B, \ge }(w, \overline{w}, u)&= {\mathbf {B}}^{0}_{B, \ge } (w, \overline{w}, w) + {\mathbf {B}}^{1}_{B,\ge } (w,\overline{w}, u) + {\mathbf {B}}^{2}_{B, \ge } (w,\overline{w}, u) + {\mathbf {B}}^{3}_{B, \ge }(w,\overline{w},w) \\&\quad + {\mathbf {B}}^{3}_{B, \ge } \big (w, \overline{w}, \varphi _T\cdot {\mathbf {G}}_{A, \ge }[w, \overline{u},u]\big ) + {\mathbf {B}}^{3}_{B, \ge } \big (w, \overline{w}, \varphi _T\cdot {\mathbf {G}}_{A,>}[w, u,\overline{u}]\big )\\&\quad + {\mathbf {B}}^{3}_{B, \ge } \big (w, \overline{u}, \varphi _T\cdot {\mathbf {G}}_{B, \ge }[w, \overline{w},u]\big ) + {\mathbf {B}}^{3}_{B, \ge } \big (w, \overline{u}, \varphi _T\cdot {\mathbf {G}}_{B, >}[w, w,\overline{u}]\big ). \end{aligned}$$

Proceeding as detailed above, we obtain a new equation for *w*. Due to its length, we have decided to only include it in “Appendix A”.

### Proposition 4.2

For any $u_0\in {\mathcal {F}}L^{\frac{1}{2},p}({\mathbb {T}})$ satisfying $\Vert u_0\Vert _{{\mathcal {F}}L^{\frac{1}{2},p}} \le A_1$, there exists a unique $w\in Z_0$ with $\Vert w\Vert _{Z_0} \le A_2$ satisfying (4.2), for some $T=T(A_1,A_2,A_3)>0$. The mapping $u_0 \mapsto w$ is Lipschitz from the $A_1$-ball of ${\mathcal {F}}L^{\frac{1}{2},p}({\mathbb {T}})$ to the $A_2$-ball of $Z_0$.

### Remark 4.3

In order to show Proposition 4.2, we will not run a contraction mapping argument for the map defined by the right-hand side of (4.2) nor the equation (A.1) included in “Appendix A”. Some quintic terms in (A.1) require the use of the equation for *u* (4.1) once again, introducing new quintic terms but also new septic terms. Given the considerable number of new terms that this additional step introduces, we have decided to omit them when presenting the equation for *w*. The strategy for obtaining the new contributions is described in Sect. 6.2 along with the estimates needed for both the quintic and septic terms.

### Remark 4.4

In Sects. 5 and 6, we will establish the estimates needed to show Propositions 4.1 and 4.2, from which Theorem 1.3 follows for $s=\frac{1}{2}$. In order to extend this result to $s>\frac{1}{2}$, note that the required estimates in

$$\begin{aligned} Y'_0 = X^{s,\frac{1}{2}}_{p,r_0}, \quad Z'_0 = X^{s,b_0}_{p,q_0}, \end{aligned}$$

follow easily from the estimates shown by associating the extra weight $\langle n \rangle ^{s-\frac{1}{2}}$ in the norm to the function with the largest frequency. Consequently, by a persistence of regularity argument, we obtain a unique solution $u\in C([-T,T];{\mathcal {F}}L^{s,p}({\mathbb {T}}))$ where $T = T\big ( \Vert u_0\Vert _{{\mathcal {F}}L^{\frac{1}{2},p}} \big )>0$.

In the remaining of the paper, we will establish the nonlinear estimates needed to show Propositions 4.1 and 4.2. The results follow from a contraction mapping argument in $Y_0$ and $Z_0$, respectively. From Lemma 2.5, it suffices to estimate the terms in Eqs. (4.1) and (A.1) in $Y_1$ and $Z_1$, respectively, dropping the factors of $\varphi _T$. For simplicity, we will use the notation ${\mathcal {D}}\mathcal {NR}_{*}$ to denote ${\mathcal {D}}\mathcal {NR}_{*, \ge }$, ${\mathcal {D}}\mathcal {NR}_{*,>}$, for $*\in \{A,B,C,D\}$, ${\mathbf {G}}_*$ to denote ${\mathbf {G}}_{*, \ge }, {\mathbf {G}}_{*,>}$ for $*\in \{A,B\}$, and ${\mathbf {B}}^{j}_{*}$ for ${\mathbf {B}}^{j}_{*, \ge }, {\mathbf {B}}^{j}_{*,>}$ for $*\in \{A,B\}$ and $j=0,1,2,3$. In the estimates, there is no distinction between the frequency regions where $|n_2|\ge |n_3|$ and $|n_2| > |n_3|$, motivating this simplified notation.

## Proof of Proposition 4.1

In this section, we establish the estimates needed to show Proposition 4.1.

### Lemma 5.1

Let $*\in \{A,B\}$. Then, the following estimate holds

$$\begin{aligned} \Vert {\mathbf {G}}_{*} (u_1,u_2,u_3) \Vert _{Y_1}&\lesssim \Vert u_1\Vert _{Z_0} \Vert u_2\Vert _{Y_0} \Vert u_3\Vert _{Y_0} . \end{aligned}$$

### Proof

Using (3.5) and the change of variables $\tau _j + n^3_j = \sigma _j$, $j=1,2,3$, it follows that

5.1 $$\begin{aligned}&\Vert {\mathbf {G}}_* (u_1,u_2,u_3) \Vert _{Y_1}\nonumber \\&\quad \!\!\!\lesssim \bigg \Vert \sum _{\begin{array}{c} n=n_1+n_2+n_3, \\ {\overline{n}}_{123} \in {\mathbb {X}}_*(n) \end{array}} {{\,\mathrm{\int }\,}}_{\mathbb {R}}\frac{\langle n \rangle ^\frac{1}{2} |n_1| }{\langle \tau - \lambda \rangle \langle \Phi ({\overline{n}}_{123}) \rangle ^\frac{1}{2}} {{\,\mathrm{\int }\,}}_{\lambda = \tau _1 + \tau _2 + \tau _3} \prod _{j=1}^3 |\widehat{u}_j (\tau _j, n_j)| \ d\lambda \bigg \Vert _{\ell ^p_n L^{r_1}_{\tau }} \nonumber \\&\quad \!\!\!\lesssim \bigg \Vert \sum _{\begin{array}{c} n=n_1+n_2+n_3, \\ {\overline{n}}_{123} \in {\mathbb {X}}_*(n) \end{array}} \!{{\,\mathrm{\int }\,}}_{\sigma _1, \sigma _2 , \sigma _3} \frac{\langle n \rangle ^\frac{1}{2} |n_1| }{\langle \tau -n^3 - {\bar{\sigma }} + \Phi ({\overline{n}}_{123}) \rangle \langle \Phi ({\overline{n}}_{123}) \rangle ^\frac{1}{2}} \prod _{j=1}^3 |\widehat{u}_j (\sigma _j + n_j^3, n_j)| \bigg \Vert _{\ell ^p_n L^{r_1}_{\tau }}, \end{aligned}$$

where ${\bar{\sigma }} = \sigma _1 + \sigma _2 + \sigma _3$. Note that

$$\begin{aligned} \frac{\langle n \rangle ^\frac{1}{2}|n_1|}{\langle \Phi ({\overline{n}}_{123}) \rangle ^\frac{1}{2}} \lesssim \frac{\langle n \rangle ^\frac{1}{2} |n_1|}{\max _{j=1,2,3}\langle n_j \rangle } \lesssim \langle n_1 \rangle ^\frac{1}{2}. \end{aligned}$$

Minkowski’s inequality gives

$$\begin{aligned}&\Vert {\mathbf {G}}_* (u_1,u_2,u_3) \Vert _{Y_1}\\&\qquad \lesssim {{\,\mathrm{\int }\,}}_{\sigma _1, \sigma _2, \sigma _3} \bigg \Vert \sum _{\begin{array}{c} n=n_1+n_2+n_3, \\ {\overline{n}}_{123} \in {\mathbb {X}}_*(n) \end{array}} \frac{\langle n_1 \rangle ^\frac{1}{2}}{\langle \tau - n^3 - {\bar{\sigma }} + \Phi ({\overline{n}}_{123}) \rangle } \prod _{j=1}^3 |\widehat{u}_j (\sigma _j + n_j^3, n_j)| \bigg \Vert _{\ell ^p_n L^{r_1}_{\tau }} . \end{aligned}$$

Denoting the inner norm by $\text {I}$, we can rewrite the sum as follows

$$\begin{aligned} \text {I}&\lesssim \Bigg \Vert \sum _{\mu } \frac{1}{\langle \tau - n^3 - {\bar{\sigma }} + \mu \rangle } \Bigg ( \sum _{\begin{array}{c} n=n_1+n_2+n_3, \\ {\overline{n}}_{123} \in {\mathbb {X}}_*^\mu (n) \end{array}} \langle n_1 \rangle ^\frac{1}{2} \prod _{j=1}^3 |\widehat{u}_j(\sigma _j + n_j^3, n_j)| \Bigg ) \Bigg \Vert _{\ell ^{p}_n L^{r_1}_\tau }, \end{aligned}$$

for $X^\mu _*(n)$ in (2.4). Since the following bounds hold uniformly in ${\bar{\sigma }}$ and *n*, for any ${\tilde{r}}>1$,

$$\begin{aligned} \sum _\mu \frac{1}{\langle \tau - n^3 - {\bar{\sigma }} + \mu \rangle ^{{\tilde{r}}}} \lesssim 1, \qquad {{\,\mathrm{\int }\,}}_{\mathbb {R}}\frac{1}{\langle \tau - n^3 - {\bar{\sigma }} + \mu \rangle ^{{\tilde{r}}}} d\tau \lesssim 1, \end{aligned}$$

choosing ${\tilde{r}} = \frac{1}{1-\delta }$, we have $\frac{1}{r_1} + 1 = \frac{1}{{\tilde{r}}} + \frac{1}{r_2}$ and we can apply Schur’s test^2^ to obtain

5.2 $$\begin{aligned} \text {I}&\lesssim \Bigg \Vert \sum _{\begin{array}{c} n=n_1+n_2+n_3, \\ {\overline{n}}_{123} \in {\mathbb {X}}^\mu _*(n) \end{array}} \langle n_1 \rangle ^\frac{1}{2} \prod _{j=1}^3 |\widehat{u}_j(\sigma _j + n_j^3,n_j)| \Bigg \Vert _{\ell ^{p}_n \ell ^{r_2}_{\mu }}. \end{aligned}$$

Let ${\mathbf {P}}_{N_j}$ denote the projection onto $\langle n \rangle \sim N_j$ and let $f_1(\sigma , n) = \langle n \rangle ^\frac{1}{2} |\widehat{u}_1(\sigma +n^3, n)|$, $f_j(\sigma ,n) = | \widehat{{\mathbf {P}}_{N_j} u}_j(\sigma +n^3, n)|$, $j=2,3$. Then, using Minkowski and Hölder’s inequalities, we get

$$\begin{aligned} \text {I}&\lesssim \sum _{N_2,N_3} \bigg \Vert |{\mathbb {X}}_*^\mu (n)|^{\frac{1}{r_2'}} \bigg (\sum _{\begin{array}{c} n=n_1+n_2+n_3,\\ {\overline{n}}_{123}\in {\mathbb {X}}_*^\mu (n) \end{array}} \prod _{j=1}^3 |f_j(\sigma _j, n_j)|^{r_2} \bigg )^{\frac{1}{r_2}} \bigg \Vert _{\ell ^{p}_n \ell ^{r_2}_{\mu }}. \end{aligned}$$

If $*=A$, we have $|\Phi ({\overline{n}}_{123})| \lesssim |n_1|^3 \sim |n|^3$, so we use Lemma 2.7 to count the divisors $d_2=n-n_2, d_3=n-n_3$ of $\mu$. Since

$$\begin{aligned} |d_2 - n| = |n_2| \le N_2, \quad |d_3 - n| = |n_3| \le N_3 \end{aligned}$$

and $1 \le |\mu |^\varepsilon \le |\mu |^\frac{1}{3} \lesssim |n|$, for any $0<\varepsilon \le \frac{1}{3}$, we conclude that there are at most ${\mathcal {O}}(N_j^\varepsilon )$ choices for $d_j$, $j=2,3$. Since *n* is fixed, this determines the choices of $n_2,n_3$ and consequently of $n_1$. If $*=B$, then $|\Phi ({\overline{n}}_{123})| \lesssim |n_2|^3$ and we can use the standard divisor counting estimate to conclude that there are at most ${\mathcal {O}}(N_2^\varepsilon )$ choices for $n_2,n_3$. Consequently, $|{\mathbb {X}}_*^\mu (n)| \lesssim (N_2N_3)^\varepsilon$ and we have

$$\begin{aligned} \text {I}&\lesssim \sum _{N_2,N_3} (N_2 N_3)^\varepsilon \bigg \Vert \bigg ( \sum _\mu \sum _{\begin{array}{c} n=n_1+n_2+n_3, \\ {\overline{n}}_{123}\in {\mathbb {X}}^\mu _*(n) \end{array}} \prod _{j=1}^3 |f_j(\sigma _j, n_j)|^{r_2} \bigg )^{\frac{1}{r_2}} \bigg \Vert _{\ell ^{p}_n }\\&\lesssim \sum _{N_2,N_3} (N_2 N_3)^\varepsilon \bigg \Vert \bigg ( \sum _{\begin{array}{c} n=n_1+n_2+n_3 \end{array}} \bigg (\sum _\mu \mathbb {1}_{\Phi ({\overline{n}}_{123}) = \mu } \bigg ) \prod _{j=1}^3 |f_j(\sigma _j, n_j)|^{r_2} \bigg )^{\frac{1}{r_2}} \bigg \Vert _{\ell ^{p}_n }\\&\lesssim \sum _{N_2,N_3} (N_2N_3)^\varepsilon \Vert f_1\Vert _{\ell ^p_n} \Vert f_2\Vert _{\ell ^{r_2}_n} \Vert f_3\Vert _{\ell ^{r_2}_n}, \end{aligned}$$

where we apply Minkowski’s inequality and the fact that $r_2<2\le p$ in the last inequality. Choosing $\varepsilon < \delta$, we obtain

$$\begin{aligned} \text {I}\lesssim \Vert \langle n \rangle ^\frac{1}{2} \widehat{u}_1(\sigma _1+n^3,n)\Vert _{\ell ^p_n} \Vert \langle n \rangle ^\delta \widehat{u}_2(\sigma _2 + n^3, n)\Vert _{\ell ^{r_2}_n} \Vert \langle n \rangle ^\delta \widehat{u}_3(\sigma _3 + n^3, n)\Vert _{\ell ^{r_2}_n}. \end{aligned}$$

Applying this estimate to (5.2) gives

$$\begin{aligned} \Vert {\mathbf {G}}_* (u_1,u_2,u_3) \Vert _{Y_1}&\lesssim \Vert \langle n \rangle ^\frac{1}{2} \widehat{u}_1(\sigma +n^3,n)\Vert _{L^1_\sigma \ell ^p_n} \prod _{j=2}^3 \Vert \langle n \rangle ^\delta \widehat{u}_j(\sigma + n^3, n)\Vert _{L^1_\sigma \ell ^{r_2}_n} . \end{aligned}$$

The estimate follows from Hölder and Minkowski’s inequalities.

$$\square $$

### Remark 5.2

The change of variables from $\tau _j$ to the modulation $\sigma _j = \tau _j - n_j^3$, $j=1,2,3$, in (5.1) is needed to guarantee that the quantity

$$\begin{aligned} \frac{1}{\langle \tau -\lambda \rangle ^{1-\delta }}&= \frac{1}{\langle \tau -n^3 - \sigma _1 - \sigma _2 - \sigma _3 + \Phi ({\overline{n}}_{123}) \rangle ^{1-\delta }} \end{aligned}$$

has an explicit dependence on the resonance relation $\Phi ({\overline{n}}_{123})$ and that when fixing its value, $\Phi ({\overline{n}}_{123}) = \mu$, there is no longer dependence on the variables $n_1, n_2,n_3$. Thus, one can consider the quantity inside the norm as a convolution operator in $\mu$, depending on $\tau$:

$$\begin{aligned} \sum _\mu \frac{1}{\langle \tau - n^3 - \sigma _1 - \sigma _2 - \sigma _3 + \mu \rangle ^{1-\delta }} F(\mu , n_1,n_2,n_3). \end{aligned}$$

This trick allows us to estimate the norm in $\tau$ and introduce a restriction on the value of the resonance relation. This strategy will be used throughout the paper.

## Proof of Proposition 4.2

In this section, we show the multilinear estimates needed to prove Proposition 4.2 by a contraction mapping argument. In particular, we estimate the trilinear and quintilinear operators on the right-hand side of (A.1). In Sect. 6.1, we focus on the cubic terms in (A.1), namely

6.1 $$\begin{aligned} \begin{aligned} \mathcal {IR}(u_1,u_2,u_3),&{\mathcal {D}}\mathcal {NR}_C(w_1,w_2,w_3) ,&{\mathcal {D}}\mathcal {NR}_D(w_1,w_2,w_3), \\ {\mathbf {B}}^{0}_A(w_1,u_2,u_3),&{\mathbf {B}}^{1}_A(w_1,u_2,u_3),&{\mathbf {B}}^{2}_A(w_1,w_2,u_3),&{\mathbf {B}}^{3}_A(w_1,u_2,w_3), \\ {\mathbf {B}}^{0}_B(w_1,w_2,u_3),&{\mathbf {B}}^{1}_B(w_1,w_2,u_3),&{\mathbf {B}}^{2}_B(w_1,w_2,u_3),&{\mathbf {B}}^{3}_B(w_1,w_2,w_3), \end{aligned}\nonumber \\ \end{aligned}$$

where $$u_j \in \{u, \overline{u}\}$$, $$w_j\in \{w,\overline{w}\}$$, $$j=1,2,3$$.

The quintic terms in (A.1) arise from substituting a *u* entry by an ${\mathbf {G}}_{\#}$-operator, for $\#\in \{A,B\}$. First, note that

$$\begin{aligned}&\big |{{\mathcal {F}}}_{t,x} \big (\varphi _T \cdot {\mathbf {G}}_{\#} (u_1,u_2,u_3) \big ) (\tau , n) \big |\\&\quad \lesssim \sum _{{\overline{n}}_{123}\in {\mathbb {X}}_{\#}(n)} {{\,\mathrm{\int }\,}}_{{\mathbb {R}}} \frac{|n_1|}{\langle \tau -\mu ' \rangle ^{1-\theta } \langle \Phi ({\overline{n}}_{123}) \rangle } {{\,\mathrm{\int }\,}}_{\mu ' = \tau _1 + \tau _2 + \tau _3} \prod _{j=1}^3 |\widehat{u}_j (\tau _j, n_j)| \ d\mu ' , \\&\big |{{\mathcal {F}}}_{t,x} \overline{\big (\varphi _T \cdot {\mathbf {G}}_{\#} (u_1,u_2,u_3) \big )} (\tau , n) \big | \\&\quad \lesssim \sum _{{\overline{n}}_{123}\in {\mathbb {X}}_{\#}(n)} {{\,\mathrm{\int }\,}}_{{\mathbb {R}}} \frac{|n_1|}{\langle \tau - \mu ' \rangle ^{1-\theta } \langle \Phi ({\overline{n}}_{123}) \rangle } {{\,\mathrm{\int }\,}}_{\mu ' = \tau _1 + \tau _2 + \tau _3} \prod _{j=1}^3 |\widehat{\overline{u}}_j (\tau _j, n_j)| \ d\mu ', \end{aligned}$$

for any $0<\theta \ll 1$. Since $\Vert \overline{u} \Vert _{X^{s,b}_{p,q}} = \Vert u\Vert _{X^{s,b}_{p,q}}$ for any choice of *s*, *b*, *p*, *q*, we will omit the contributions that depend on $\overline{{\mathbf {G}}_{\#}}$, as they can be estimated analogously. We start by calculating the space-time Fourier transform of the quintic contributions arising from $\mathcal {DN}$ terms. For example, for $*\in \{A,B,C,D\}$ and $\#\in \{A,B\}$, we have the following estimate

$$\begin{aligned}&\big |{{\mathcal {F}}}_{t,x} \big ( \mathcal {DN}_* \big ( \varphi _T \cdot {\mathbf {G}}_{\#}[u_1,u_2,u_3], u_4,u_5 \big ) (\tau ,n) \big | \\&\quad \lesssim \sum _{\begin{array}{c} {\overline{n}}_{045} \in {\mathbb {X}}_*(n),\\ {\overline{n}}_{123} \in {\mathbb {X}}_{\#}(n_0) \end{array}} {{\,\mathrm{\int }\,}}_{\mathbb {R}}{{\,\mathrm{\int }\,}}_{\lambda = \tau _1 + \ldots + \tau _5} \frac{|n_0n_1|}{\langle \tau -\lambda \rangle ^{1-\theta } \langle \tau -n^3 \rangle \langle \Phi ({\overline{n}}_{123}) \rangle } \prod _{j=1}^5 |\widehat{u}_j(\tau _j, n_j)| \, d\lambda , \end{aligned}$$

for some $0<\theta <1$. Similar estimates can be obtained for the contributions $\mathcal {DN}_*\big (u_1,\varphi _T \cdot {\mathbf {G}}_{\#}[u_2,u_3,u_4], u_5\big )$ and $\mathcal {DN}_*\big (u_1, u_2,\varphi _T \cdot {\mathbf {G}}_{\#}[u_3,u_4,u_5]\big )$, $*\in \{B,C,D\}$, $\#\in \{A,B\}$. The main difficulty is controlling the spatial multiplier defined as follows

$$\begin{aligned}\alpha (n, {\overline{n}}_{0\ldots 5}) = {\left\{ \begin{array}{ll} \displaystyle \frac{|n_0n_1|}{|\Phi ({\overline{n}}_{123})|} , &{}\text {if } {\overline{n}}_{045} \in {\mathbb {X}}_*(n), \ {\overline{n}}_{123} \in {\mathbb {X}}_{\#}(n_0) , \\ \displaystyle \frac{|n_1n_2|}{|\Phi ({\overline{n}}_{234})|} , &{} \text {if }{\overline{n}}_{105} \in {\mathbb {X}}_*(n), \ {\overline{n}}_{234} \in {\mathbb {X}}_{\#}(n_0) , \\ \displaystyle \frac{|n_1n_3|}{|\Phi ({\overline{n}}_{345})|} , &{} \text {if } {\overline{n}}_{120} \in {\mathbb {X}}_*(n), \ {\overline{n}}_{345} \in {\mathbb {X}}_{\#}(n_0) .\\ \end{array}\right. } \end{aligned}$$

We will refer to the frequencies in ${\mathbb {X}}_*(n)$ as the first generation of frequencies and those in ${\mathbb {X}}_{\#}(n_0)$ as the second when referring to the quintic terms.

In Sect. 6.2, we will estimate the contributions for which $\alpha (n, {\overline{n}}_{0\ldots 5}) \lesssim 1$, namely

6.2 $$\begin{aligned} \begin{aligned} {\mathcal {D}}\mathcal {NR}_{*} \big ( \varphi _T \cdot {\mathbf {G}}_{A}[w_1,u_2,u_3], u_4,u_5\big ),&{\mathcal {D}}\mathcal {NR}_{*} \big ( \varphi _T \cdot {\mathbf {G}}_{B}[w_1,w_2,u_3], u_4,u_5\big ), \\ {\mathcal {D}}\mathcal {NR}_{\#} \big ( w_1, \varphi _T \cdot {\mathbf {G}}_{A}[w_2,u_3,u_4],u_5\big ),&{\mathcal {D}}\mathcal {NR}_{\#} \big ( w_1, \varphi _T \cdot {\mathbf {G}}_{B}[w_2,w_3,u_4],u_5\big ), \\ {\mathcal {D}}\mathcal {NR}_{D} \big ( w_1, w_2, \varphi _T \cdot {\mathbf {G}}_{A}[w_3,u_4,u_5]\big ),&{\mathcal {D}}\mathcal {NR}_{D} \big ( w_1, w_2, \varphi _T \cdot {\mathbf {G}}_{B}[w_3,w_4,u_5]\big ), \end{aligned} \end{aligned}$$

where $*\in \{A,B,C,D\}$, $\#\in \{B,C,D\}$ and $u_j\in \{u,\overline{u}\}$, $w_j \in \{w, \overline{w}\}$, $j=1, \ldots , 5$. The estimate for these contributions follows once we control ${\mathcal {Q}}(u_1,\ldots , u_5)$ defined by its space-time Fourier transform as follows

6.3 $$\begin{aligned}&{{\mathcal {F}}}_{t,x}\big ({\mathcal {Q}}(u_1, \ldots , u_5)\big ) (\tau ,n) \nonumber \\&\quad = \sum _{n=n_1+\ldots +n_5} {{\,\mathrm{\int }\,}}_{\mathbb {R}}{{\,\mathrm{\int }\,}}_{\lambda = \tau _1 + \ldots + \tau _5} \frac{1}{\langle \tau -\lambda \rangle ^{1-\theta } \langle \tau -n^3 \rangle } \prod _{j=1}^5 |\widehat{u}_j(\tau _j, n_j)| \, d\lambda . \end{aligned}$$

In Sect. 6.2, we establish an estimate for (6.3) under particular assumptions on the frequencies. Not all the contributions in (6.2) will satisfy these additional assumptions, which forces us to use the equation for *u* once again, introducing new septic terms. It remains to consider the following quintic contributions

6.4 $$\begin{aligned} \begin{aligned} {\mathcal {D}}\mathcal {NR}_C \big ( w_1,w_2, \varphi _T \cdot {\mathbf {G}}_A[w_3,u_4,u_5] \big ),&{\mathcal {D}}\mathcal {NR}_C \big ( w_1,w_2, \varphi _T \cdot {\mathbf {G}}_B[w_3,w_4,u_5] \big ),\\ {\mathbf {B}}^{2}_A\big ( w_1, \varphi _T \cdot {\mathbf {G}}_A[w_2,u_3,u_4], u_4 \big ),&{\mathbf {B}}^{2}_A\big ( w_1, \varphi _T \cdot {\mathbf {G}}_B[w_2,w_3,u_4], u_4 \big ), \\ {\mathbf {B}}^{3}_A\big ( w_1, u_2, \varphi _T \cdot {\mathbf {G}}_A[w_3,u_4,u_5]\big ),&{\mathbf {B}}^{3}_A\big ( w_1, u_2, \varphi _T \cdot {\mathbf {G}}_B[w_3,w_4,u_5]\big ), \\ {\mathbf {B}}^{3}_B\big ( w_1, w_2, \varphi _T \cdot {\mathbf {G}}_A[w_3,u_4,u_5]\big ),&{\mathbf {B}}^{3}_B\big ( w_1, w_2, \varphi _T \cdot {\mathbf {G}}_B[w_3,w_4,u_5]\big ), \end{aligned} \end{aligned}$$

where $u_j \in \{u, \overline{u}\}$, $w_j \in \{w, \overline{w}\}$, $j=1,\ldots , 5$. The $\mathcal {DNR}_C$ contributions are not controlled by (6.3) and thus need a more refined approach. For the ${\mathbf {B}}^j_*$ contributions, not only does the *j*th modulation play an important role, but also the largest modulation of the new functions in ${\mathbf {G}}_{\#}$. This is detailed in Sect. 6.3.

### Cubic Terms

We start by estimating the cubic terms in (6.1).

#### Lemma 6.1

The following estimate holds

$$\begin{aligned} \Vert \mathcal {I R} (u_1,u_2,u_3) \Vert _{Z_1} \lesssim \Vert u_1\Vert _{Y_0} \Vert u_2\Vert _{Y_0} \Vert u_3\Vert _{Y_0}. \end{aligned}$$

#### Proof

Using the kernel estimate for ${\mathcal {D}}$ in (3.2) and Young’s inequality, we have

$$\begin{aligned} \Vert \mathcal {I R} (u_1,u_2,u_3) \Vert _{Z_1}&\lesssim \bigg \Vert {{\,\mathrm{\int }\,}}_{\mathbb {R}}\frac{1}{\langle \tau -\lambda \rangle } {{\,\mathrm{\int }\,}}_{\lambda = \tau _1 - \tau _2 + \tau _3} \prod _{j=1}^3 \langle n \rangle ^\frac{1}{2} |\widehat{u}_j(\tau _j,n)| \, d\lambda \bigg \Vert _{\ell ^p_n L^{q_0}_\tau }\\&\lesssim \bigg \Vert {{\,\mathrm{\int }\,}}_{\tau = \tau _1 - \tau _2 + \tau _3} \prod _{j=1}^3 \langle n \rangle ^\frac{1}{2} |\widehat{u}_j (\tau _j,n)| \bigg \Vert _{\ell ^p_n L^{r_0}_\tau } , \end{aligned}$$

for $\delta < \frac{1}{6}$. Applying Hölder’s inequality gives

$$\begin{aligned} \Vert \mathcal {I R} (u_1,u_2,u_3) \Vert _{Z_1}&\lesssim \sup _{\tau ,n} J(\tau ,n) \bigg \Vert \prod _{j=1}^3 \Vert \langle n \rangle ^\frac{1}{2} \langle \tau -n^3 \rangle ^\frac{1}{2} \widehat{u}_j(\tau ,n) \Vert _{L^{r_0}_\tau } \bigg \Vert _{\ell ^p_n}, \end{aligned}$$

where

$$\begin{aligned} J(\tau ,n)^{r'_0}&= {{\,\mathrm{\int }\,}}_{{\mathbb {R}}^2} \frac{d\tau _1 \, d\tau _2}{\langle \tau _1 - n^3 \rangle ^{\frac{r'_0}{2}} \langle \tau _2 - n^3 \rangle ^{\frac{r'_0}{2}} \langle \tau - \tau _1 +\tau _2 - n^3 \rangle ^{\frac{r'_0}{2}} } \lesssim 1 \end{aligned}$$

from Lemma 2.8. The result follows from Hölder’s inequality. $\square$

#### Lemma 6.2

Let $*\in \{C,D\}$. Then, the following estimate holds

$$\begin{aligned} \Vert {\mathcal {D}}\mathcal {NR}_*(u_1,u_2,u_3) \Vert _{Z_1} \lesssim \Vert u_1\Vert _{Z_0} \Vert u_2\Vert _{Z_0} \Vert u_3\Vert _{Z_0}. \end{aligned}$$

#### Proof

Let $*\in \{C,D\}$, then ${\overline{n}}_{123}\in {\mathbb {X}}_*(n)$ implies that $\langle n \rangle ^\frac{1}{2} |n_1| \lesssim \langle n_1 \rangle ^\frac{1}{2} \langle n_2 \rangle ^\frac{1}{2} \langle n_3 \rangle ^\frac{1}{2}$. Using (3.2), we have

$$\begin{aligned}&\Vert {\mathcal {D}}\mathcal {NR}_*(u_1,u_2,u_3) \Vert _{Z_1} \\&\quad \lesssim \bigg \Vert {{\,\mathrm{\int }\,}}_{\mathbb {R}}\sum _{\begin{array}{c} n=n_1+n_2+n_3,\\ {\overline{n}}_{123}\in {\mathbb {X}}_*(n) \end{array}} \frac{1}{\langle \tau -n^3 \rangle ^\delta \langle \tau -\lambda \rangle } {{\,\mathrm{\int }\,}}_{\lambda = \tau _1 + \tau _2 + \tau _3} \prod _{j=1}^3 \langle n_j \rangle ^\frac{1}{2} | \widehat{u}_j(\tau _j, n_j)| d\lambda \bigg \Vert _{\ell ^p_n L^{q_0}_\tau }. \end{aligned}$$

Let $f_j(\sigma ,n) = \langle n \rangle ^\frac{1}{2} \langle \sigma \rangle ^\delta |\widehat{u}_j (\sigma + n^3, n)|$, $j=1,2,3$, ${\bar{\sigma }}=\sigma _1+\sigma _2+\sigma _3$ and proceed as in (5.1). Using Minkowski’s and Hölder’s inequalities gives

$$\begin{aligned}&\Vert {\mathcal {D}}\mathcal {NR}_*(u_1,u_2,u_3) \Vert _{Z_1} \\&\quad \lesssim {{\,\mathrm{\int }\,}}_{\sigma _1,\sigma _2,\sigma _3} \bigg \Vert \sum _{\begin{array}{c} n=n_1+n_2+n_3,\\ {\overline{n}}_{123}\in {\mathbb {X}}_*(n) \end{array}} \frac{1}{\langle \Phi ({\overline{n}}_{123}) \rangle ^\delta \langle \tau - n^3 -{\bar{\sigma }} + \Phi ({\overline{n}}_{123}) \rangle ^{1-\delta }} \prod _{j=1}^3 f_j(\sigma _j, n_j) \bigg \Vert _{\ell ^p_n L^{q_0}_\tau } \\&\quad \lesssim {{\,\mathrm{\int }\,}}_{\sigma _1,\sigma _2,\sigma _3}\bigg \Vert \sum _{\mu } \frac{|\mu |^\varepsilon }{\langle \mu \rangle ^\delta \langle \tau - n^3-{\bar{\sigma }} + \mu \rangle ^{1-\delta }} \bigg ( \sum _{{\overline{n}}_{123} \in {\mathbb {X}}_*^\mu (n)} \prod _{j=1}^3 |f_j(\sigma _j , n_j)|^p \bigg )^\frac{1}{p} \bigg \Vert _{\ell ^p_n L^{q_0}_\tau }, \end{aligned}$$

since from the standard divisor counting estimate, we have that $|{\mathbb {X}}_*^\mu (n)| \lesssim _\varepsilon |\mu |^\varepsilon$, for any $\varepsilon >0$. Choosing $\varepsilon \le \delta$ and applying Schur’s test with $1 + \frac{1}{q_0} = \frac{1}{p} + \frac{1}{q}$, we obtain

$$\begin{aligned}&\Vert {\mathcal {D}}\mathcal {NR}_*(u_1,u_2,u_3) \Vert _{Z_1}&\lesssim {{\,\mathrm{\int }\,}}_{\sigma _1,\sigma _2,\sigma _3}\bigg \Vert \bigg ( \sum _{{\overline{n}}_{123} \in {\mathbb {X}}_*^\mu (n)} \prod _{j=1}^3 |f_j(\sigma _j , n_j)|^p \bigg )^\frac{1}{p} \bigg \Vert _{\ell ^p_n \ell ^{p}_\mu } \\&\quad \lesssim \prod _{j=1}^3 \Vert f_j(\sigma , n) \Vert _{L^1_\sigma ,\ell ^p_n}, \end{aligned}$$

for $\delta < \frac{1}{5p}$. Consequently, using Hölder’s and Minkowski’s inequalities, it follows that

$$\begin{aligned} \Vert {\mathcal {D}}\mathcal {NR}_*(u_1,u_2,u_3) \Vert _{Z_1}&\lesssim \prod _{j=1}^3 \Vert \langle n \rangle ^\frac{1}{2} \langle \sigma \rangle ^{1-3\delta +} \widehat{u}_j(\sigma + n^3, n) \Vert _{L^{q_0}_\sigma \ell ^p_n} \lesssim \prod _{j=1}^3 \Vert u_j\Vert _{Z_0}. \end{aligned}$$

$$\square $$

#### Remark 6.3

(i) The terms ${\mathcal {D}}\mathcal {NR}_A$, ${\mathcal {D}}\mathcal {NR}_B$ cannot be estimated in a similar manner because $\langle n \rangle ^\frac{1}{2} |n_1|$ is not controlled by $(\langle n_1 \rangle \langle n_2 \rangle \langle n_3 \rangle )^\frac{1}{2}$. This motivated the application of the modified Duhamel operator to introduce smoothing in space needed to control the loss of derivative from the nonlinearity, without using the largest modulation.
(ii) Consider the estimate
  $$\begin{aligned} \big \Vert {\mathcal {D}}\mathcal {NR}_D(u_1,u_2,u_3) \big \Vert _{X^{\frac{1}{2}, b}_{p,q}} \lesssim \prod _{j=1}^3 \Vert u_j\Vert _{X^{\frac{1}{2}, b}_{p,q}}, \end{aligned}$$
  for some $b\ge 0$, $2\le q < \infty$. The region ${\mathbb {X}}_D(n)$ includes the case when $|n_1| \sim |n_2| \sim |n_3|$, $\max \limits _{j=1,2,3}|n_j| \lesssim |\Phi ({\overline{n}}_{123})| \ll \max \limits _{j=1,2,3} |n_j|^2$. When attempting to show the above estimate under the nearly-resonant assumption, we must impose the conditions
  $$\begin{aligned} \max \bigg (1 - \frac{1}{2q}, 1 + \frac{1}{q} - \frac{1}{p} \bigg )&< b <1, \end{aligned}$$
  which motivate our choice of $b=1-$ and $q=\infty -$ for the definition of the $Z_0$ space.

#### Lemma 6.4

Let $*\in \{A,B\}$. The following estimate holds

$$\begin{aligned} \Vert {\mathbf {B}}_*^{0}(u_1,u_2,u_3) \Vert _{Z_1} \lesssim \Vert u_1\Vert _{Y_0} \Vert u_2\Vert _{Y_0} \Vert u_3\Vert _{Y_0}. \end{aligned}$$

#### Proof

Choosing $\alpha =4\delta$ in the kernel estimate (3.7), gives

$$\begin{aligned} \Vert {\mathbf {B}}_*^{0}(u_1,u_2,u_3) \Vert _{Z_1}&\lesssim \bigg \Vert \sum _{\begin{array}{c} n=n_1+n_2+n_3,\\ {\overline{n}}_{123}\in {\mathbb {X}}_*(n) \end{array}} {{\,\mathrm{\int }\,}}_{\tau _1,\tau _2,\tau _3} \frac{\langle n \rangle ^\frac{1}{2} |n_1| }{\langle \tau -n^3 \rangle ^{5 \delta } \langle \Phi ({\overline{n}}_{123}) \rangle ^{1-4\delta } } \prod _{j=1}^3 |\widehat{u}_j (\tau _j, n_j)| \bigg \Vert _{\ell ^p_n L^{q_0}_{\tau }}\\&\lesssim \bigg \Vert \sum _{\begin{array}{c} n=n_1+n_2+n_3,\\ {\overline{n}}_{123}\in {\mathbb {X}}_*(n) \end{array}} \frac{\langle n \rangle ^\frac{1}{2} |n_1| }{ \langle \Phi ({\overline{n}}_{123}) \rangle ^{1-4\delta } } \prod _{j=1}^3 \Vert \widehat{u}_j ( n_j)\Vert _{L^1_\tau } \bigg \Vert _{\ell ^p_n }, \end{aligned}$$

by applying Minkowski’s inequality in the last step and integrating in $\tau$. For ${\overline{n}}_{123}\in {\mathbb {X}}_*(n)$, we have $|\Phi ({\overline{n}}_{123})| \sim \max \limits _{j=1,2,3}|n_j|^2 \min \limits _{l=1,2,3}|n-n_l|$, which implies

$$\begin{aligned} \frac{\langle n \rangle ^\frac{1}{2} |n_1|}{\langle \Phi ({\overline{n}}_{123}) \rangle ^{1-4\delta }} \lesssim \frac{\langle n_1 \rangle ^\frac{1}{2}}{\max \limits _{j=1,2,3}\langle n_j \rangle ^{1 -8\delta } \min \limits _{l=1,2,3} \langle n-n_l \rangle ^{1-4\delta }}. \end{aligned}$$

Applying Hölder and Minkowski’s inequalities, it follows that

$$\begin{aligned} \Vert {\mathbf {B}}_*^{0}(u_1,u_2,u_3) \Vert _{Z_1}&\lesssim \big ( \sup _n J(n)^{\frac{1}{p'}} \big ) \Vert \langle n \rangle ^\frac{1}{2} \widehat{u}_1\Vert _{\ell ^p_n L^1_\tau } \prod _{j=2}^3 \Vert \widehat{u}_j\Vert _{\ell ^p_n L^1_\tau }, \end{aligned}$$

where *J*(*n*) is defined as follows

$$\begin{aligned} J(n):= \sum _{n=n_1+n_2+n_3} \frac{1}{\max \limits _{j=1,2,3} \langle n_j \rangle ^{(1-8\delta )p'} \min \limits _{l=1,2,3}\langle n-n_l \rangle ^{(1-4\delta )p'} }. \end{aligned}$$

Let $j,l\in \{1,2,3\}$ denote the indices at which the maximum and minimum in the definition of *J*(*n*) are attained, respectively. If $j = l$, we can use the fact that $\langle n_j \rangle \gtrsim \langle n_i \rangle$ for $i\in \{1,2,3\}{\setminus }\{j\}$ and sum in $n_i, n_j$. If $j\ne l$, we sum in $n_j, n_l$. Thus, $J(n) \lesssim 1$ uniformly in *n* for $\delta < \frac{1}{8p}$. The intended estimate, follows from Hölder’s inequality.

$$\square $$

#### Lemma 6.5

Let $*\in \{A,B\}$. Then, the following estimates hold for $j=1,2,3$

$$\begin{aligned} \Vert {\mathbf {B}}_*^{j}(u_1,u_2,u_3)\Vert _{Z_1} \lesssim \Vert u_j\Vert _{Z_0} \prod _{\begin{array}{c} k=1\\ k\ne j \end{array}}^3 \Vert u_k\Vert _{Y_0}. \end{aligned}$$

#### Proof

We will only show the estimate for $j=1$, as the remaining estimates follow an analogous proof. Let $*\in \{A,B\}$. From (3.9) with $1-\alpha = b_0 - \delta$ and for ${\overline{n}}_{123} \in {\mathbb {X}}_*(n)$ we have

$$\begin{aligned} \langle n \rangle ^\frac{1}{2} |n_1| \langle \tau -n^3 \rangle ^{b_1}\big |K_{+}\big (\tau -n^3, \lambda - n^3, \Phi ({\overline{n}}_{123})\big )\big | \lesssim \frac{\langle n_1 \rangle ^{\frac{1}{2}} \langle \tau _1 - n_1^3 \rangle ^{1-3\delta }}{\langle \tau -\lambda \rangle \langle \Phi ({\overline{n}}_{123}) \rangle ^{\frac{1}{2}-3\delta } \langle n-n_l \rangle ^{\frac{1}{2}}}, \end{aligned}$$

where $|n-n_l| = \min _{j=1,2,3}|n-n_j|$. Let $f(\tau ,n) = \langle n \rangle ^\frac{1}{2} \langle \tau _1 - n_1^3 \rangle ^{b_0 - \delta }|\widehat{u}_1(\tau ,n)|$. Then, using Minkowski’s and Young’s inequalities, we have

$$\begin{aligned}&\big \Vert {\mathbf {B}}^{1}_* (u_1,u_2,u_3) \big \Vert _{Z_1}\\&\quad \lesssim \bigg \Vert \sum _{\begin{array}{c} n=n_1+n_2+n_3,\\ {\overline{n}}_{123} \in {\mathbb {X}}_*(n) \end{array}} \frac{1}{\langle \Phi ({\overline{n}}_{123}) \rangle ^{\frac{1}{2}-3\delta } \langle n-n_l \rangle ^{\frac{1}{2}}} \bigg (\frac{1}{\langle \cdot \rangle } *f(\cdot , n_1) *_{j=2,3} |\widehat{u}_j(\cdot , n_j)|\bigg )(\tau ) \bigg \Vert _{\ell ^p_n L^{q_0}_\tau } \\&\quad \lesssim \bigg \Vert \sum _{\begin{array}{c} n=n_1+n_2+n_3,\\ {\overline{n}}_{123} \in {\mathbb {X}}_*(n) \end{array}} \frac{1}{\langle \Phi ({\overline{n}}_{123}) \rangle ^{\frac{1}{2} - 3\delta }\langle n-n_l \rangle ^{\frac{1}{2}}} \Vert f(n_1)\Vert _{L^{q_1}_\tau } \prod _{j=2}^3 \Vert \widehat{u}_j (n_j) \Vert _{L^1_\lambda } \bigg \Vert _{\ell ^p_n}. \end{aligned}$$

Using Hölder’s inequality, we obtain

$$\begin{aligned} \big \Vert {\mathbf {B}}^{1}_* (u_1,u_2,u_3) \big \Vert _{Z_1} \lesssim \bigg (\sup _n J(n)\bigg )^{\frac{1}{p'}} \Vert f\Vert _{\ell ^p_n L^{q_1}_\tau } \prod _{j=2}^3 \Vert u_j\Vert _{\ell ^p_n L^1_\tau } , \end{aligned}$$

where

$$\begin{aligned} J(n) = \sum _{n=n_1+n_2+n_3} \frac{1}{\langle n_{\max } \rangle ^{p'(1-6\delta )} \langle n-n_l \rangle ^{p'(1-3\delta )}} \lesssim \sum _{n_i, n_l} \frac{1}{\langle n_i \rangle ^{p'(1-6\delta )} \langle n-n_l \rangle ^{p'(1-3\delta )}} \lesssim 1, \end{aligned}$$

for some distinct $n_i, n_l \in \{n_1,n_2,n_3\}$ and $\delta < \frac{1}{6p}$. The intended estimate follows from applying Hölder’s inequality.

$$\square $$

### Standard Quintic Terms

In this section, we focus on estimating the quintic terms in (6.2). Before doing so, we must take into account the new ‘resonances’ introduced by using the second iteration. For the estimates to hold, we need the largest frequency to correspond to a *w* term and not be in a pairing, as defined below. Otherwise, we will use the equation for *u* (4.1), which introduces new septic terms.

Looking at ${\mathcal {Q}}$ in (6.3) in more detail, note that the sum in (6.3) over $n=n_1+\cdots + n_5$ does not exclude all resonances, i.e., we can have $n_i + n_j =0$ for distinct $i, j \in \{1,\ldots , 5\}$. If this holds, we say that (*i*, *j*) is a pairing.

We will show a general estimate for ${\mathcal {Q}}$ in (6.3), given that one of the following holds:

(i) There are no pairings in $(n_1, \ldots , n_5)$ and the largest frequency corresponds to a function in $Z_0$;
(ii) There is one pairing (*i*, *j*) and the largest frequency in $\{|n_k|: \ 1\le k \le 5, \ k\ne i,j \}$ corresponds to a function in $Z_0$;
(iii) There are two pairings and the remaining frequency corresponds to a function in $Z_0$.

Note that if (i), (ii) or (iii) hold, we can always use the largest frequency which is not in a pairing to control the spatial weight from the norm $\langle n \rangle ^\frac{1}{2}$. If the contributions do not satisfy any of the above conditions, then the largest frequency that is not in a pairing corresponds to a function *u* and we want to use the equation for *u* again. This leads to one quintic term that satisfies the assumptions above and two septic terms, which are easily estimated.

To further clarify, let ${\mathcal {Q}}'(u_1, \ldots , u_5)$ denote a contribution in (6.2), $u_j \in \{u, \overline{u}, w, \overline{w}\}$. Let $n_j$ correspond to the spatial Fourier variable of $\widehat{u}_j$, $j=1,\ldots ,5$. If $n_1$ is the largest frequency that is not in a pairing and $u_1 \in \{w,\overline{w}\}$, then we keep the contribution as is. Otherwise, $u_1 \in \{u,\overline{u}\}$ and we will use the equation (4.1) to replace the first entry in ${\mathcal {Q}}'$. For simplicity, assume that $u_1 = u$, then we have

$$\begin{aligned}&{\mathcal {Q}}' (u_1, \ldots , u_5) \\&\quad = {\mathcal {Q}}' (w, u_2, \ldots , u_5) \\&\qquad \quad + {\mathcal {Q}}' \big (\varphi _T\cdot {\mathbf {G}}_{A,\ge }[w, \overline{u}, u], u_2, \ldots , u_5 \big ) + {\mathcal {Q}}'\big (\varphi _T\cdot {\mathbf {G}}_{A,>}[w, u, \overline{u}], u_2 \ldots , u_5\big ) \\&\qquad \quad + {\mathcal {Q}}' \big (\varphi _T\cdot {\mathbf {G}}_{B,\ge }[w, \overline{w}, u], u_2, \ldots , u_5\big ) + {\mathcal {Q}}' \big (\varphi _T\cdot {\mathbf {G}}_{B,>}[w, w, \overline{u}], u_2, \ldots , u_5\big ). \end{aligned}$$

By carefully examining the frequencies and pairings of the terms in (6.2) and applying the above modification, we obtain the final equation for *w*. Due to its length, we have decided to not include the full equation.

All the resulting quintic and septic terms arising from (6.2), can be estimated by the two following propositions.

#### Proposition 6.6

Let ${\mathcal {Q}}$ as defined in (6.3) where the first factor has the largest spatial Fourier frequency which is not in a pairing. Then, the following estimate holds

$$\begin{aligned} \big \Vert {\mathcal {Q}}(u_1, \ldots , u_5) \big \Vert _{Z_1} \lesssim \Vert u_1\Vert _{Z_0} \prod _{j=2}^5 \Vert u_j\Vert _{Y_0}. \end{aligned}$$

#### Proof

**Case 1: no pairing**

Let ${\mathbf {P}}_{N_j}$ denote the Dirichlet projection onto $\langle n_j \rangle \sim N_j$, $j=1,\ldots , 5$. Since there is no pairing we have $|n| \lesssim |n_1|$, therefore using Minkowski’s inequality gives

$$\begin{aligned} \Vert {\mathcal {Q}}(u_1, \ldots , u_5) \Vert _{Z_1}&\lesssim \sum _{N_2, \ldots , N_5} {{\,\mathrm{\int }\,}}_{\tau _1, \ldots , \tau _5} \bigg \Vert \sum _{n=n_1 + \ldots + n_5} \frac{1}{\langle \tau - \tau _1 - \ldots - \tau _5 \rangle ^{1-\theta }}\\&\quad \times \langle n_1 \rangle ^\frac{1}{2} |\widehat{u}_1 (\tau _1,n_1)| \prod _{j=2}^5 |\widehat{{\mathbf {P}}_{N_j} u}(\tau _j,n_j)| \bigg \Vert _{\ell ^p_n L^{q_0}_\tau }. \end{aligned}$$

Using the change of variables $\sigma _j = \tau _j - n_j^3$, $j=1,\ldots , 5$, and Schur’s test, we get

$$\begin{aligned} \Vert {\mathcal {Q}} (u_1, \ldots , u_5) \Vert _{Z_1}&\lesssim \sum _{N_2, \ldots , N_5} {{\,\mathrm{\int }\,}}_{\sigma _1,\ldots , \sigma _5} \bigg \Vert \sum _{n=n_1 + \ldots + n_5} \frac{1}{\langle \tau - n^3 - \sigma _1 - \ldots - \sigma _5 + \Psi (n, {\overline{n}}_{1\ldots 5}) \rangle } \\&\quad \times \langle n_1 \rangle ^\frac{1}{2} |\widehat{u}_1 (\sigma _1 + n_1^3,n_1)| \prod _{j=2}^5 |\widehat{{\mathbf {P}}_{N_j} u}(\sigma _j + n_j^3,n_j)| \bigg \Vert _{\ell ^p_n L^{q_0}_\tau } \ d\sigma _1 \cdots d\sigma _5 \\&\lesssim \sum _{N_2, \ldots , N_5} {{\,\mathrm{\int }\,}}_{\sigma _1, \ldots , \sigma _5} \bigg \Vert \sum _{\begin{array}{c} n=n_1 + \ldots + n_5,\\ \Psi (n,{\overline{n}}_{1\ldots 5})= \mu \end{array}} \prod _{j=1}^5 f_j(\sigma _j, n_j) \bigg \Vert _{\ell ^p_n \ell ^{q_1}_\mu }, \end{aligned}$$

where $\Psi (n, {\overline{n}}_{1\ldots 5}) = n^3 - n_1^3 - \ldots - n_5^3$, $f_1(\sigma ,n) = \langle n \rangle ^\frac{1}{2} |\widehat{u}_1(\sigma +n^3,n)|$ and $f_j(\sigma ,n) = |\widehat{P_{N_j} u_j}(\sigma +n^3,n)|$, $j=2,\ldots ,5$. Note that we can trivially restrict $\mu$ to the following region

$$\begin{aligned} A(n,N_2,\ldots , N_5)&= \big \{ \mu \in {\mathbb {Z}}: \ \mu = n - (n-n_2 - \ldots - n_5)^3 - n_2^3 - \ldots - n_5^3 , \\&\quad |n_j|\sim N_j, \ j=2,\ldots ,5 \big \}, \end{aligned}$$

which satisfies $|A(n,N_2,\ldots ,N_5)| \lesssim N^4_2$ for fixed *n*. Thus, it follows from Hölder’s inequality in $\mu$ that

6.5 $$\begin{aligned} \Vert {\mathcal {Q}} (u_1, \ldots , u_5)\Vert _{Z_1}&\sim \sum _{N_2, \ldots , N_5} {{\,\mathrm{\int }\,}}_{\sigma _1, \ldots , \sigma _5} \bigg \Vert \mathbb {1}_{\mu \in A(n,N_2, \ldots , N_5)}\sum _{\begin{array}{c} n=n_1 + \ldots + n_5,\\ \Psi (n,{\overline{n}}_{1\ldots 5})= \mu \end{array}} \prod _{j=1}^5 f_j(\sigma _j,n_j) \bigg \Vert _{\ell ^p_n \ell ^{q_1}_\mu } \nonumber \\&\lesssim \sum _{N_2, \ldots , N_5} N_2^{\frac{4}{q_1}} {{\,\mathrm{\int }\,}}_{\sigma _1, \ldots , \sigma _5} \bigg \Vert \sum _{\begin{array}{c} n=n_1 + \ldots + n_5,\\ \Psi (n,{\overline{n}}_{1\ldots 5})= \mu \end{array}}\prod _{j=1}^5 f_j(\sigma _j,n_j) \bigg \Vert _{\ell ^p_n \ell ^\infty _\mu }. \end{aligned}$$

**Subcase 1.1: **$$N_3 \ge N_2^{4\sqrt{\delta }}$$

Using Cauchy’s inequality with $\alpha >0$, omitting the time dependence, we have

$$\begin{aligned} \sum _{\begin{array}{c} n=n_1 + \ldots + n_5,\\ \Psi (n,{\overline{n}}_{1\ldots 5})= \mu \end{array}} \prod _{j=1}^5 f_j (n_j)&\lesssim \sum _{\begin{array}{c} n=n_1 + \ldots + n_5,\\ \Psi (n,{\overline{n}}_{1\ldots 5})= \mu \end{array}} f_1(n_1) \big (\alpha |f_2(n_2) f_3(n_3)|^2 + \alpha ^{-1}|f_4(n_4)f_5(n_5)|^2 \big ) \\&\lesssim \sum _{n_2,n_3} \sum _{(n_1,n_4, n_5)\in B(n,n_2,n_3,\mu )} \alpha f_1(n_1) |f_2(n_2) f_3(n_3)|^2 \\&\quad + \sum _{n_4,n_5} \sum _{(n_1, n_2, n_3)\in B(n,n_4,n_5,\mu )} \alpha ^{-1} f_1(n_1) |f_4(n_4) f_5(n_5)|^2, \end{aligned}$$

where

$$\begin{aligned} B(k,k_1,k_2,\mu ) := \big \{ (n_1,n_2,n_3)\in {\mathbb {Z}}^3: \ n_1 + n_2 + n_3 = k - k_1 - k_2 =: l , \\ 3(n_2+n_3) (l - n_2)(l - n_3) = \mu - k^3 + k_1^3 + k_2^3 + l^3 \big \}. \end{aligned}$$

Taking a supremum in $n_1$, we obtain

$$\begin{aligned} \sum _{\begin{array}{c} n=n_1 + \ldots + n_5,\\ \Psi (n,{\overline{n}}_{1\ldots 5})= \mu \end{array}} \prod _{j=1}^5 f_j (n_j)&\lesssim \alpha \sup _{|n-n_1| \lesssim N_2} f_1(n_1) \sum _{n_2,n_3} |B(n,n_2,n_3, \mu )| \cdot |f_2(n_2)f_3(n_3)|^2 \\&\quad + \alpha ^{-1} \sup _{|n-n_1| \lesssim N_2} f_1(n_1) \sum _{n_4,n_5} |B(n,n_4,n_5, \mu )| \cdot |f_4(n_4)f_5(n_5)|^2. \end{aligned}$$

In order to estimate $|B(n, n_2,n_3, \mu )|, |B(n, n_4,n_5, \mu )|$, we use Lemma 2.7. For the first one, to count the choices of $(n_1,n_4,n_5)$ it suffices to count the number of divisors $l-n_4, l-n_5$, where $l=n-n_2-n_3$, of ${\tilde{\Psi }}:=3(n_4+n+5)(n-n_2-n_3-n_4)(n-n_2-n_3-n_5) = 3(n_4+n_5)(l-n_4)(l-n_5)$. If $|n|\gg |n_2|$, then

$$\begin{aligned} |{\tilde{\Psi }}| \sim |(n_4 + n_5)(n-n_2-n_3-n_4)(n-n_2-n_3-n_5)| \lesssim |n|^3 \implies |{\tilde{\Psi }}|^\varepsilon \le |{\tilde{\Psi }}|^\frac{1}{3} \lesssim |n|, \end{aligned}$$

for any $\varepsilon >0$. Otherwise, $|n| \lesssim |n_2|$ and we have

$$\begin{aligned} |{\tilde{\Psi }}|&\sim |(n_4 + n_5)(n-n_2-n_3-n_4)(n-n_2-n_3-n_5)| \lesssim |n_2|^3 \implies |{\tilde{\Psi }}|^\varepsilon \\&\quad \le |{\tilde{\Psi }}|^\frac{1}{3} \lesssim |n_2|, \end{aligned}$$

for any $$\varepsilon >0$$. Applying the lemma, the number of divisors $$d_4 = n-n_2-n_3-n_4 $$, $$d_5 = n-n_2-n_3-n_5$$ satisfying

$$\begin{aligned} {\left\{ \begin{array}{ll} |d_j - n| = |n_2+n_3+n_j| \lesssim N_2 ,&{} \text {if } |n| \gg |n_2|,\\ |d_j - n_2| = |n - n_3 - n_j| \lesssim N_2, &{} \text {if } |n| \lesssim |n_2|, \end{array}\right. } \end{aligned}$$

is bounded by $N_2^\varepsilon$, for $j=4,5$. Thus, $|B(n,n_2,n_3, \mu )| \lesssim N_2^\varepsilon$, for any $\varepsilon >0$. An analogous approach gives $|B(n,n_4,n_5, \mu )| \lesssim N_2^\varepsilon$. Consequently, we have

$$\begin{aligned} \sum _{\begin{array}{c} n=n_1 + \ldots + n_5,\\ \Psi (n,{\overline{n}}_{1\ldots 5})= \mu \end{array}} \prod _{j=1}^5 f_j (n_j)&\lesssim N_2^\varepsilon \sup _{|n-n_1| \lesssim N_2} f_1(n_1) \prod _{j=2}^5 \Vert f_j\Vert _{\ell ^2_n}, \end{aligned}$$

by choosing $\alpha = (\Vert f_2\Vert _{\ell ^2_n} \Vert f_3\Vert _{\ell ^2_n})^{-1}\Vert f_4\Vert _{\ell ^2_n} \Vert f_5\Vert _{\ell ^2_n}$. Looking at (6.5), since $|n-n_1| \lesssim N_2$, taking a supremum in $n_1$ gives

$$\begin{aligned}&\Vert {\mathcal {Q}}(u_1, \ldots , u_5)\Vert _{Z_1}\\&\quad \lesssim \sum _{N_2,\ldots , N_5} N_2^{\frac{4}{q_1} + \varepsilon } {{\,\mathrm{\int }\,}}_{\sigma _1, \ldots , \sigma _5}\bigg \Vert \sup _{|n-n_1|\lesssim N_2} f_1(\sigma _1,n_1) \bigg \Vert _{l^p_n} \prod _{j=2}^5 \Vert f_j(\sigma _j)\Vert _{ \ell ^2_n} \\&\quad \lesssim \sum _{N_2,\ldots , N_5} N_2^{\frac{4}{q_1} + \varepsilon } {{\,\mathrm{\int }\,}}_{\sigma _1, \ldots , \sigma _5} \bigg (\sum _{n_1} |f_1(\sigma _1,n_1)|^p \sum _{|n-n_1|\lesssim N_2} 1 \bigg )^{\frac{1}{p}} \prod _{j=2}^5 \Vert f_j(\sigma _j)\Vert _{ \ell ^2_n} \\&\quad \lesssim \sum _{N_2, \ldots , N_5} N_2^{\frac{4}{q_1} + \frac{1}{p} + \varepsilon } \Vert f_1\Vert _{L^1_\sigma \ell ^p_n} \prod _{j=2}^5 \Vert f_j\Vert _{L^1_\sigma \ell ^2_n}. \end{aligned}$$

Using Hölder’s and Minkowski’s inequalities, we have

$$\begin{aligned} \Vert {\mathcal {Q}}(u_1, \ldots , u_5)\Vert _{Z_1}&\lesssim \sum _{N_2,\ldots , N_5} N_2^{\frac{4}{q_1} + \frac{1}{p}+ \varepsilon } (N_2 N_3 N_4 N_5)^{ \delta - \frac{1}{p} +} \Vert u_1 \Vert _{Z_0} \prod _{j=2}^5 \Vert u_j \Vert _{Y_0} . \end{aligned}$$

It only remains to sum in the dyadic numbers $N_j$. Using the fact that $N_3 \ge N_2^{4\sqrt{\delta }}$, we have

$$\begin{aligned} \sum _{N_2, \ldots , N_5} N_2^{\frac{4}{q_1} + \delta + } ( N_3 N_4 N_5)^{\delta - \frac{1}{p} +}&\lesssim \sum _{N_2, \ldots , N_5} (N_2 N_3 N_4 N_5)^{-\delta } N_3^{2\delta + 5\sqrt{\delta } - \frac{1}{p} +} (N_4 N_5 )^{2\delta - \frac{1}{p}+} \lesssim 1 \end{aligned}$$

for $$\delta < \frac{1}{2p}$$ and $$2\delta + 5\sqrt{\delta } - \frac{1}{p}<0 \implies 0< \sqrt{\delta } < -\frac{5}{4} + \sqrt{\big (\frac{5}{4}\big )^2 + \frac{1}{2p}}$$.

**Subcase 1.2:** $$N_3 \le N_2^{4\sqrt{\delta }}$$

Using Cauchy–Schwarz inequality, we have

$$\begin{aligned}&\sum _{\begin{array}{c} n=n_1 + \ldots + n_5,\\ \Psi (n,{\overline{n}}_{1\ldots 5})= \mu \end{array}} \prod _{j=1}^5 f_j (n_j) \\&\quad = \sum _{n_3, n_4, n_5} \sum _{n_1 \in C(n, n_3,n_4,n_5, \mu )} f_1(n_1) f_2(n-n_1 - n_3 - n_4 - n_5) f_3 (n_3) f_4(n_4) f_5(n_5)\\&\quad \lesssim N_2^\varepsilon \sum _{n_3, n_4, n_5} f_3 (n_3) f_4(n_4) f_5(n_5) \big (\sup _{n_1} f_1(n_1) f_2(n-n_1 - n_3 - n_4 - n_5) \big ) \end{aligned}$$

where

$$\begin{aligned} C(n, n_3,n_4,n_5, \mu ) : = \{n_1 \in {\mathbb {Z}}: l:= n_3 + n_4 + n_5, |l| \lesssim |n-n_1-l| \lesssim |n_1|, \\ |n-n_1 -l|\lesssim N_2, \ 3(n-l)(n_1 + l)(n-n_1) = \mu - l^3 + n_3^3 + n_4^3 + n_5^3\}, \end{aligned}$$

for which $|C(n,n_3,n_4,n_5, \mu )| \lesssim N_2^\varepsilon$ for any $\varepsilon >0$ from Lemma 2.7. Note that if $|n| \gg |n-n_1 - l|$, then ${\tilde{\Psi }}:= 3(n-l)(n_1 + l)(n-n_1)$ satisfies $|{\tilde{\Psi }}| \lesssim |n|^3$ and $|{\tilde{\Psi }}|^\varepsilon \le |{\tilde{\Psi }}|^\frac{1}{3} \lesssim |n|$ for any $0<\varepsilon <\frac{1}{3}$. Counting the number of $n_1$ is equivalent to counting the number of divisors $d = n_1 + l$. Since $|d - n| = |n-n_1-l| \lesssim N_2$, from Lemma 2.7, there exist at most $N_2^\varepsilon$ values for $n_1$. If $|n| \lesssim |n-n_1-l|$, then $|{\tilde{\Psi }}| \lesssim |n-n_1-l|^3\lesssim N_2^3$, so by the standard divisor counting lemma, there are at most $N_2^\varepsilon$ divisors $n_1+l$. Consequently, $|C(n,n_3,n_4,n_5, \mu )| \lesssim N_2^\varepsilon$, for any $0<\varepsilon <\frac{1}{3}$.

Minkowski’s inequality gives the following

$$\begin{aligned} \bigg \Vert \sum _{\begin{array}{c} n=n_1 + \ldots + n_5,\\ \Psi (n,{\overline{n}}_{1\ldots 5})= \mu \end{array}} \prod _{j=1}^5 f_j (n_j) \bigg \Vert _{\ell ^p_n \ell ^\infty _\mu }&\lesssim N_2^\varepsilon \Vert f_1\Vert _{\ell ^p_n} \Vert f_2\Vert _{\ell ^p_n} \prod _{j=3}^5 \Vert f_j\Vert _{\ell ^1_n}. \end{aligned}$$

Applying the previous estimate to (6.5) and Hölder’s inequality give

$$\begin{aligned} \Vert {\mathcal {Q}}(u_1, \ldots , u_5)\Vert _{Z_1}&\lesssim \sum _{N_2, \ldots , N_5} N_2^{\frac{4}{q_1} + \varepsilon } \Vert f_1\Vert _{L^1_\sigma \ell ^p_n} \Vert f_2\Vert _{L^1_\sigma \ell ^p_n} \prod _{j=3}^5 \Vert f_j\Vert _{\ell ^1_n L^1_\lambda } \\&\lesssim \sum _{N_2, \ldots , N_5} N_2^{\frac{4}{q_1} + \frac{1}{r_0} - \frac{1}{p} - \frac{1}{2}+} (N_3 N_4 N_5)^{\frac{1}{2} - \frac{1}{p} +} \Vert u_1\Vert _{Z_0} \prod _{j=2}^5 \Vert u_j\Vert _{Y_0}. \end{aligned}$$

It only remains to sum in the dyadics $N_j$:

$$\begin{aligned} \sum _{N_2, \ldots , N_5} N_2^{\frac{4}{q_1} + \delta - \frac{1}{p}+ } (N_3 N_4 N_5)^{\frac{1}{2} - \frac{1}{p}+}&\lesssim \sum _{N_2, \ldots , N_5} (N_3 N_4 N_5)^{-\delta } N_2^{\frac{4}{q_1} + \delta - \frac{1}{p} + 12\sqrt{\delta }(\frac{1}{2} - \frac{1}{p} + 3 \sqrt{\delta }) } \lesssim 1 \end{aligned}$$

if $$\frac{4}{q_1} + \delta - \frac{1}{p} + 12\sqrt{\delta }(\frac{1}{2} - \frac{1}{p} + 3 \sqrt{\delta })<0 \implies 0< \sqrt{\delta } < - \frac{6}{21}\big (\frac{1}{2} - \frac{1}{p}\big ) + \sqrt{\frac{6^2}{21^2}\big (\frac{1}{2} - \frac{1}{p}\big )^2 + \frac{21}{p}}$$.

**Case 2: one pairing (4, 5)**

In this case, we have $n=n_1 + n_2 + n_3$, $n_4 + n_5 =0$. Let $f_1(\sigma ,n) = \langle n \rangle ^\frac{1}{2} |\widehat{u}_1(\sigma +n^3,n)|$ and $f_j(\sigma ,n) = |\widehat{{\mathbf {P}}_{N_j} u_j} (\sigma +n^3, n)|$, $j=2,3$. Using Cauchy–Schwarz inequality in $n_4$ and proceeding as in Case 1 gives the following

6.6 $$\begin{aligned}&\Vert {\mathcal {Q}}(u_1, \ldots , u_5)\Vert _{Z_1}\nonumber \\&\quad \lesssim \sum _{N_2,N_3} {{\,\mathrm{\int }\,}}_{\sigma _1,\sigma _2,\sigma _3} \Bigg \Vert \sum _{\begin{array}{c} n=n_1+n_2+n_3\\ \Phi ({\overline{n}}_{123}) = \mu \end{array}} \prod _{j=1}^3 f_j(\sigma _j,n_j) \Bigg \Vert _{\ell ^p_n \ell ^{q_1}_\mu } \prod _{k=4}^5\Vert \widehat{u}_k \Vert _{L^1_\tau \ell ^2_n} \nonumber \\&\quad \lesssim \sum _{N_2,N_3} {{\,\mathrm{\int }\,}}_{\sigma _1, \sigma _2, \sigma _3} \Bigg \Vert \mathbb {1}_{\mu \in A(n,N_2,N_3)} \sum _{\begin{array}{c} n=n_1+n_2+n_3,\\ \Phi ({\overline{n}}_{123}) = \mu \end{array}} \prod _{j=1}^3 f_j(\sigma _j, n_j) \Bigg \Vert _{\ell ^p_n } \prod _{k=4}^5\Vert \widehat{u}_k \Vert _{L^1_\tau \ell ^2_n} \nonumber \\&\quad \lesssim \sum _{N_2,N_3} N_2^{\frac{2}{q_1}} {{\,\mathrm{\int }\,}}_{\sigma _1,\sigma _2,\sigma _3} \Bigg \Vert \sum _{\begin{array}{c} n=n_1+n_2+n_3,\\ \Phi ({\overline{n}}_{123}) = \mu \end{array}} \prod _{j=1}^3 f_j(\sigma _j, n_j) \Bigg \Vert _{\ell ^p_n \ell ^\infty _\mu } \prod _{k=4}^5\Vert \widehat{u}_k \Vert _{L^1_\tau \ell ^2_n}, \end{aligned}$$

where $A(n,N_2,N_3) := \big \{ \mu \in {\mathbb {Z}}: \ \mu = n^3 - (n-n_2-n_3)^3 - n_2^3 - n_3^3, \ |n_j| \sim N_j, j=2,3 \big \}$, which satisfies $|A(n,N_2,N_3)| \lesssim N_2^2$ uniformly in *n*.

**Subcase 2.1:** $$N_2^{4\sqrt{\delta }} \le N_3$$

Focusing on the inner sum, we apply Cauchy’s inequality, with $\alpha >0$, to obtain the following

$$\begin{aligned}&\sum _{\begin{array}{c} n=n_1+n_2+n_3,\\ \Phi ({\overline{n}}_{123}) = \mu \end{array}} f_1( n_1) f_2( n_2) f_3(n_3)\\&\quad \lesssim \sum _{n_2} \sum _{n_1 \in B(n,n_2,\mu )} \alpha f_1(n_1) |f_2(n_2)|^2 + \sum _{n_3} \sum _{n_1 \in B(n,n_3,\mu )} \alpha ^{-1} f_1(n_1) |f_3( n_3)|^2 , \end{aligned}$$

where $B(n,n_j, \mu ) = \big \{ n_1\in {\mathbb {Z}}: \ 3(n-n_1)(n-n_j)(n_1+n_j) = \mu \big \}$, $j=2,3$. Note that $|B(n,n_j,\mu )| \le 2$ because the given equation is quadratic in $n_1$. Thus, taking a supremum in $n_1$ and using the fact that $|n-n_1|\lesssim N_2$, we get

$$\begin{aligned} \sum _{\begin{array}{c} n=n_1+n_2+n_3,\\ \Phi ({\overline{n}}_{123}) = \mu \end{array}} \prod _{j=1}^3 f_j(\sigma _j, n_j)&\lesssim \sup _{|n-n_1|\lesssim N_2} f_1(\sigma _1,n_1) \big (\alpha \Vert f_2(\sigma _2)\Vert _{\ell ^2_n}^2 + \alpha ^{-1} \Vert f_3(\sigma _3)\Vert _{\ell ^2_n}^2 \big ) \\&\lesssim \sup _{|n-n_1|\lesssim N_2} f_1(\sigma _1,n_1) \prod _{j=2}^3 \Vert f_j(\sigma _j)\Vert _{\ell ^2_n}, \end{aligned}$$

by choosing $\alpha = \Vert f_2(\sigma _2)\Vert _{\ell ^2_n}^{-1} \Vert f_3(\sigma _3) \Vert _{\ell ^2_n}$. Using this estimate on ${\mathcal {Q}}$ gives

$$\begin{aligned} \Vert {\mathcal {Q}}(u_1, \ldots , u_5)\Vert _{Z_1}&\lesssim \sum _{N_2, N_3} N_2^{\frac{2}{q_1}+\frac{1}{p}} \Vert f_1\Vert _{L^1_{\sigma }\ell ^p_n} \bigg (\prod _{j=2}^3 \Vert f_j\Vert _{L^1_\sigma \ell ^2_n} \bigg ) \bigg (\prod _{k=4}^5 \Vert \widehat{u}_k\Vert _{L^1_\tau \ell ^2_n}\bigg ) \\&\lesssim \sum _{N_2,N_3} N_2^{\frac{2}{q_1} + \frac{1}{p}} (N_2 N_3)^{\frac{1}{r_0} - \frac{1}{p} - \frac{1}{2} +} \Vert u_1\Vert _{Z_0} \prod _{j=2}^5 \Vert u_j\Vert _{Y_0}. \end{aligned}$$

The estimate follows from summing in the dyadics.

**Subcase 2.2:** $$N_2^{4\sqrt{\delta }} \ge N_3$$

Focusing on the spatial norm on (6.6), we have

$$\begin{aligned}&\Bigg \Vert \sum _{\begin{array}{c} n=n_1+n_2+n_3,\\ \Phi ({\overline{n}}_{123}) = \mu \end{array}} \prod _{j=1}^3 f_j(\sigma _j,n_j) \Bigg \Vert _{\ell ^p_n \ell ^{\infty }_\mu } \\&\quad \lesssim \bigg \Vert \sum _{n_3} \bigg ( \sum _{n_1 \in C(n,n_3,\mu )} f_1(\sigma _1,n_1) f_2(\sigma _2, n-n_1-n_3) \bigg ) f_3(\sigma _3,n_3) \bigg \Vert _{\ell ^p_n \ell ^\infty _\mu }\\&\quad \lesssim \sum _{n_3} f_3(\sigma _3,n_3) \big \Vert \sup _{n_1} f_1(\sigma _1,n_1) f_2(\sigma _2,n-n_1-n_3) \big \Vert _{\ell ^p_n}, \end{aligned}$$

where $C(n,n_3,\mu ) = \big \{ n_1\in {\mathbb {Z}}: 3(n-n_1) (n-n_3) (n_1+n_3) = \mu \big \}$ satisfies $|C(n,n_3,\mu )| \le 2$. Substituting this estimate in (6.6) and using Hölder’s inequality gives

$$\begin{aligned} \Vert {\mathcal {Q}}(u_1, \ldots , u_5)\Vert _{Z_1}&\lesssim \sum _{N_2,N_3} N_2^{\frac{2}{q_1} + \frac{1}{r_0} - \frac{1}{p} - \frac{1}{2} + } N_3^{\frac{1}{2} - \frac{1}{p}+} \Vert u_1\Vert _{Z_0} \prod _{j=2}^5 \Vert u_j\Vert _{Y_0}. \end{aligned}$$

The estimate follows from summing in the dyadics.

**Case 3: two pairings (2, 3), (4, 5)**

Using Minkowski’s and Cauchy–Schwarz inequalities, we get the following

$$\begin{aligned}&\Vert {\mathcal {Q}}(u_1, \ldots , u_5)\Vert _{Z_1} \\&\quad \lesssim \bigg \Vert \Vert \langle n \rangle ^\frac{1}{2} \widehat{u}_1(n)\Vert _{L^1_\tau } \sum _{n_2} \Vert \widehat{u}_2(n_2)\Vert _{L^1_\tau } \Vert \widehat{u}_3( -n_2)\Vert _{L^1_\tau } \sum _{n_4} \Vert \widehat{u}_4(n_4)\Vert _{L^1_\tau } \Vert \widehat{u}_5 (- n_4)\Vert _{L^1_\tau } \bigg \Vert _{\ell ^p_n }\\&\quad \lesssim \Vert \langle n \rangle ^\frac{1}{2} \widehat{u}_1 \Vert _{\ell ^p_n L^1_\tau } \prod _{j=2}^5 \Vert \widehat{u}_j \Vert _{\ell ^2_n L^1_\tau }. \end{aligned}$$

The result follows from Hölder’s inequality.

$$\square $$

#### Remark 6.7

Note that the above estimate still holds if we include a factor of $\langle n_j \rangle ^\varepsilon$ in the multiplier, for some $j\in \{2,\ldots , 5\}$ and a small $0<\varepsilon \ll 1$.

#### Proposition 6.8

Let the first entry in ${\mathcal {Q}}$ have the highest frequency which is not associated to a pairing and $\#\in \{A,B\}$. Then, the following estimates hold

6.7 $$\begin{aligned}&\big \Vert {\mathcal {Q}} \big ( \varphi _T \cdot {\mathbf {G}}_{\#}[u_1,u_2,u_3], u_4,\ldots , u_7\big ) \big \Vert _{Z_1} , \big \Vert {\mathcal {Q}} \big ( \overline{\varphi _T \cdot {\mathbf {G}}_{\#}[u_1,u_2,u_3]}, u_4,\ldots , u_7\big )\big \Vert _{Z_1} \nonumber \\&\quad \lesssim \Vert u_1 \Vert _{Z_0} \prod _{j=2}^7 \Vert u_j\Vert _{Y_0}. \end{aligned}$$

#### Proof

Note that the left-hand side of (6.7) is controlled by the following quantity

6.8 $$\begin{aligned} \bigg \Vert \sum _{n=n_1 + \ldots + n_7} {{\,\mathrm{\int }\,}}_{{\mathbb {R}}} \frac{\langle n \rangle ^\frac{1}{2} |n_1|}{\langle \Phi ({\overline{n}}_{123}) \rangle \langle \tau - \lambda \rangle ^{1-\theta } } {{\,\mathrm{\int }\,}}_{\lambda = \tau _1 + \ldots + \tau _7} \prod _{j=1}^7 |\widehat{u}_j(\tau _j, n_j)| \ d\lambda \bigg \Vert _{\ell ^p_n L^{q_0}_\tau } , \end{aligned}$$

with $\widehat{u}_j$ substituted by $\widehat{\overline{u}}_j$, $j=1,2,3$ for the second term. It suffices to estimate (6.8).

Since $(n_1,n_2,n_3)\in {\mathbb {X}}_*(n_0)$ and $|n_0| = \max (|n_0|, |n_4|, \ldots , |n_7|)$, we have

$$\begin{aligned} \frac{\langle n \rangle ^\frac{1}{2} |n_1|}{\langle \Phi ({\overline{n}}_{123}) \rangle } \lesssim \frac{1}{\max \limits _{j=1,\ldots ,7}\langle n_j \rangle ^\frac{1}{2}}. \end{aligned}$$

Consider the change of variables $\sigma _j = \tau _j - n_j^3$, $j=1,\ldots , 7$ and let $f_j(\sigma , n) = | \widehat{{\mathbf {P}}_{N_j} u}_j(\sigma + n^3, n)|$, $j=1, \ldots , 7$.

**Case 1: no pairings**

Assume that $*=A$. Then, $|n_1| \ge |n_j|$, $j=2,\ldots ,7$. Using Minkowski’s inequality and Schur’s test, we have

$$\begin{aligned} (6.8)&\lesssim \sum _{N_1, \ldots , N_7} N_1^{-\frac{1}{2}} {{\,\mathrm{\int }\,}}_{{\mathbb {R}}^7} \bigg \Vert \sum _{n=n_1 + \ldots + n_7} \frac{1}{\langle \tau - \tau _1 - \ldots - \tau _7 + \Psi (n, {\overline{n}}_{1\ldots 7}) \rangle ^{1-\theta }} \prod _{j=1}^7 f_j(\sigma _j, n_j) \bigg \Vert _{\ell ^p_n L^{q_0}_\tau }\\&\lesssim \sum _{N_1, \ldots , N_7} {{\,\mathrm{\int }\,}}_{{\mathbb {R}}^7} N_1^{-\frac{1}{2}} \Bigg \Vert \sum _{\begin{array}{c} n=n_1+ \ldots + n_7,\\ \Psi (n,{\overline{n}}_{1\ldots 7} ) = \mu \end{array}} \prod _{j=1}^7 f_j(\sigma _j, n_j) \Bigg \Vert _{\ell ^p_n \ell ^{q_1}_{\mu }}, \end{aligned}$$

where $\Psi (n,{\overline{n}}_{1\ldots 7}) = n^3 -n_1^3 - \ldots - n_7^3$. Using Hölder’s inequality, it follows that

$$\begin{aligned} (6.8)&\lesssim \sum _{N_1, \ldots , N_7} {{\,\mathrm{\int }\,}}_{{\mathbb {R}}^7} N_1^{-\frac{1}{2}} \Bigg \Vert \mathbb {1}_{\mu \in A(n,N_1, \ldots , N_7)}\sum _{\begin{array}{c} n=n_1+ \ldots + n_7,\\ \Psi (n,{\overline{n}}_{1\ldots 7} ) = \mu \end{array}} \prod _{j=1}^7 f_j(\sigma _j, n_j) \Bigg \Vert _{\ell ^p_n \ell ^{q_1}_{\mu }} \\&\lesssim \sum _{N_1, \ldots , N_7} {{\,\mathrm{\int }\,}}_{{\mathbb {R}}^7} N_1^{-\frac{1}{2}} (N_2 \cdots N_7)^{\frac{1}{q_1}} \bigg \Vert \sum _{\begin{array}{c} n=n_1+ \ldots + n_7,\\ \Psi (n,{\overline{n}}_{1\ldots 7} ) = \mu \end{array}} \prod _{j=1}^7 f_j(\sigma _j, n_j) \Bigg \Vert _{\ell ^p_n \ell ^{\infty }_{\mu }}, \end{aligned}$$

where

$$\begin{aligned} A(n, N_1, \ldots ,N_7)&= \big \{ \mu \in {\mathbb {Z}}: \ \mu = n^3 - (n-n_2-\ldots -n_7)^3 - n_2^3 - \ldots - n_7^3, \\&\quad |n_j|\sim N_j, j=2, \ldots , 7 \big \} \end{aligned}$$

which satisfies $|A(n,N_2, \ldots , N_7)| \lesssim N_2 \cdots N_7$, uniformly in *n*. Focusing on the inner sum and omitting the time dependence, we have for $\alpha >0$

$$\begin{aligned} \sum _{\begin{array}{c} n=n_1+ \ldots + n_7,\\ \Psi (n,{\overline{n}}_{1\ldots 7} ) = \mu \end{array}} \prod _{j=1}^7 f_j(n_j)&\lesssim \sum _{(n_2, n_3, n_4)} \sum _{n_1} f_1(n_1)\sum _{\begin{array}{c} (n_5,n_6,n_7) \\ \in B(n,n_1,n_2,n_3, n_4, \mu ) \end{array}} \alpha |f_2(n_2) f_3(n_3) f_4(n_4)|^2 \\&\quad + \sum _{(n_5,n_6,n_7)} \sum _{n_1} f_1(n_1) \sum _{\begin{array}{c} (n_2, n_3, n_4)\\ \in B(n,n_1,n_5,n_6,n_7, \mu ) \end{array}} \alpha ^{-1}|f_5(n_5) f_6(n_6) f_7(n_7)|^2 , \end{aligned}$$

where

$$\begin{aligned} B(n,n_1,n_2,n_3,n_4, \mu )&= \big \{ (n_5,n_6,n_7)\in {\mathbb {Z}}^3: \ n_5 + n_6 + n_7 = n-n_1 - n_2 - n_3 - n_4, \\&\quad n_5^3 + n_6^3 + n_7^3 = n^3 - n_1^3 - n_2^3 - n_3^3 - n_4^3 - \mu \big \}. \end{aligned}$$

Using Lemma 2.7, we have that $|B(n,n_1, \ldots , n_4, \mu )|, |B(n, n_1, n_5, n_6, n_7, \mu )| \lesssim N_{2+}^\varepsilon$, for any $\varepsilon >0$ and $N_{2+} = \max (N_2, \ldots , N_7)$. In addition, we know that $|n-n_1| \lesssim N_{2+}$, giving

$$\begin{aligned} \sum _{\begin{array}{c} n=n_1+ \ldots + n_7,\\ \Psi (n,{\overline{n}}_{1\ldots 7} ) = \mu \end{array}} \prod _{j=1}^7 f_j(n_j)&\lesssim N_{2+}^\varepsilon \bigg ( \sum _{|n-n_1| \lesssim N_{2+}} f_1(n_1) \bigg ) \prod _{j=2}^7 \Vert f_j\Vert _{\ell ^2_n}, \end{aligned}$$

by choosing $\alpha = (\Vert f_2\Vert _{\ell ^2_n} \Vert f_3\Vert _{\ell ^2_n}\Vert f_4\Vert _{\ell ^2_n})^{-1} \Vert f_5\Vert _{\ell ^2_n} \Vert f_6\Vert _{\ell ^2_n}\Vert f_7\Vert _{\ell ^2_n}$. Consequently, using Hölder’s and Minkowski’s inequality gives the following

$$\begin{aligned} (6.8)&\lesssim \sum _{N_1, \ldots , N_7} N_1^{\frac{1}{2} -\frac{1}{p} + } N_{2+}^{\frac{1}{p} + \varepsilon } (N_2 \cdots N_7)^{\frac{1}{q_1}} \Vert f_1\Vert _{L^1_\tau \ell ^p_n} \prod _{j=1}^7 \Vert f_j\Vert _{L^1_\sigma \ell ^2_n}\\&\lesssim \sum _{N_1, \ldots , N_7} N_1^{ - \frac{1}{p} +} N_{2+}^{ \frac{1}{p} + \varepsilon } (N_2 \cdots N_7)^{\frac{1}{q_1} + \frac{1}{r_0} - \frac{1}{p} - \frac{1}{2} +} \Vert u_1 \Vert _{Z_0} \prod _{j=2}^7 \Vert u_j\Vert _{Y_0}. \end{aligned}$$

The estimate follows from summing in the dyadics. If $*=B$, then $|n_2|$ is the largest frequency and we can proceed as for $*=A$ by swapping the roles of $u_1$ and $u_2$.

**Case 2: at least one pairing**

If there is at least one pairing, we can show a stronger estimate, where every function belongs in $Y_0$, due to the negative power of the largest frequency which is not in a pairing. This case will follow a similar strategy to the proof of Proposition 6.6, thus we will omit the details.

$$\square $$

### Remaining Quintic Terms

It remains to estimate the terms in (6.4). These terms cannot be written as (6.3) and thus require a finer analysis. For the ${\mathbf {B}}^j_*$ terms, we need to use the modulations. For example, calculating the space-time Fourier transform of the ${\mathbf {B}}_*^3$ terms in (6.4), $*\in \{A,B\}$, we have

$$\begin{aligned}&\big | {{\mathcal {F}}}_{t,x} {\mathbf {B}}^{3}_{*}\big (u_1, u_2, \varphi _T \cdot {\mathbf {G}}_{\#} [u_3, u_4,u_5]\big ) (\tau , n) \big | \\&\quad \lesssim \sum _{\begin{array}{c} {\overline{n}}_{120}\in {\mathbb {X}}_*(n), \\ {\overline{n}}_{345} \in {\mathbb {X}}_{\#}(n_0) \end{array}} {{\,\mathrm{\int }\,}}_{{\mathbb {R}}^3} {{\,\mathrm{\int }\,}}_{\begin{array}{c} \lambda = \tau _1 + \tau _2 + \tau _0,\\ \sigma = \tau _3 + \tau _4 + \tau _5 \end{array}} |n_1n_3| \big | K_{+} (\tau -n^3, \lambda - n^3, \Phi ({\overline{n}}_{120})) \big |\\&\qquad \times \frac{ \mathbb {1}_{|\tau _0 - n_0^3| \gtrsim |\lambda - n^3 + \Phi ({\overline{n}}_{120})|}}{\langle \tau _0 - \mu \rangle \langle \mu - \sigma \rangle } \min \bigg (\frac{1}{\langle \Phi ({\overline{n}}_{345}) \rangle }, \frac{1}{\langle \tau _0 - n_0^3 \rangle }\bigg ) \prod _{j=1}^5 |\widehat{u}_j(\tau _j, n_j)| \ d \sigma \ d \mu \ d \lambda , \end{aligned}$$

using (3.5). In order to control the multiplier, we must consider two cases depending on the modulations of the second generation:

6.9 $$\begin{aligned} |\tau _0 - n_0^3|&\gg |\sigma - n_0^3| , \end{aligned}$$

6.10 $$\begin{aligned} |\tau _0 - n_0^3|&\lesssim |\sigma - n_0^3| . \end{aligned}$$

If (6.9) holds, then $|\tau _0 - \sigma | \sim |\tau _0 - n_0^3| \gtrsim |\lambda - n^3 + \Phi ({\overline{n}}_{105})|$ and we can obtain powers of the resonance relation of the first and the second generations:

$$\begin{aligned}&\mathbb {1}_{|\tau _0 - n_0^3| \gg |\sigma - n_0^3|} \big | {{\mathcal {F}}}_{t,x} {\mathbf {B}}^{3}_{*}\big (u_1, u_2, \varphi _T \cdot {\mathbf {G}}_{\#} [ u_3,u_4, u_5] \big ) (\tau , n) \big | \\&\quad \lesssim \sum _{\begin{array}{c} {\overline{n}}_{120}\in {\mathbb {X}}_*(n), \\ {\overline{n}}_{345} \in {\mathbb {X}}_{\#}(n_0) \end{array}} {{\,\mathrm{\int }\,}}_{\mathbb {R}}\frac{\max \limits _{j=1, \ldots , 5} \langle n_j \rangle ^{9\theta } |n_1n_3|}{\langle \Phi ({\overline{n}}_{120}) \rangle \langle \Phi ({\overline{n}}_{345}) \rangle \langle \tau -n^3 \rangle ^{1+4\delta }} {{\,\mathrm{\int }\,}}_{\sigma ' = \tau _1 + \ldots + \tau _5 } \prod _{j=1}^5 |\widehat{u}_j(\tau _j, n_j)| \, d\sigma '. \end{aligned}$$

If (6.10) holds, we can only gain a power of the resonance relation of the first generation

$$\begin{aligned}&\mathbb {1}_{|\tau _0 - n_0^3| \lesssim |\sigma - n_0^3|} \big | {{\mathcal {F}}}_{t,x} {\mathbf {B}}^{3}_{*}\big (u_1, u_2,\varphi _T \cdot {\mathbf {G}}_{\#} [ u_3,u_4, u_5] \big ) (\tau , n) \big | \\&\quad \lesssim \sum _{\begin{array}{c} {\overline{n}}_{120}\in {\mathbb {X}}_*(n), \\ {\overline{n}}_{345} \in {\mathbb {X}}_{\#}(n_0) \end{array}} {{\,\mathrm{\int }\,}}_{\mathbb {R}}\frac{\max \limits _{j=1, \ldots , 5} \langle n_j \rangle ^{9\theta }|n_1n_3|}{\langle \Phi ({\overline{n}}_{120}) \rangle \langle \tau -n^3 \rangle ^{1+\theta } \langle \tau -\sigma ' \rangle ^{1-\theta }} {{\,\mathrm{\int }\,}}_{\sigma ' = \tau _1 + \ldots + \tau _5 } \prod _{j=1}^5 |\widehat{u}_j(\tau _j, n_j)| \, d\sigma '. \end{aligned}$$

Analogous estimates hold for the ${\mathbf {B}}^2_A$ contributions in (6.4), where the sums are over ${\overline{n}}_{105}\in {\mathbb {X}}_A(n)$ and ${\overline{n}}_{234}\in {\mathbb {X}}_{\#}(n_0)$, $\#\in \{A,B\}$. For the ${\mathbf {B}}^j_*$ contributions restricted to the region where (6.10) holds and the $\mathcal {DNR}_C$ terms in (6.4), we start by looking at the spatial frequencies in more detail. In the frequency regions where the spatial multipliers can be bounded, the contributions are controlled by (6.3) and we can use Proposition 6.6. Otherwise, we can apply the following result.

#### Proposition 6.9

Assume that the frequencies are ordered as follows $|n_1| \ge \cdots \ge |n_5|$. If $|n_1| \sim |n_2| \gg |n_3|\gtrsim |n|$, (1, 2) not a pairing and

$$\begin{aligned} \alpha (n,n_1, \ldots , n_5) \lesssim \frac{|n_1| \max \limits _{j=1, \ldots , 5}|n_j|^{3\theta }}{|n_3|}, \end{aligned}$$

then the following estimate holds

6.11 $$\begin{aligned}&\bigg \Vert \langle n \rangle ^\frac{1}{2} \langle \tau -n^3 \rangle ^{b_1} \sum _{n=n_1+\ldots +n_5} {{\,\mathrm{\int }\,}}_{\mathbb {R}}\frac{\alpha (n, n_1, \ldots , n_5)}{\langle \tau -n^3 \rangle ^{1-\theta } \langle \tau - \sigma ' \rangle ^{1-\theta }} {{\,\mathrm{\int }\,}}_{\sigma ' = \tau _1 + \ldots + \tau _5} \prod _{j=1}^5 |\widehat{u}_j (\tau _j, n_j)| \ d\sigma ' \bigg \Vert _{\ell ^p_n L^{q_0}_\tau } \nonumber \\&\quad \lesssim \bigg (\prod _{j=1}^3\Vert u_j\Vert _{Z_0}\bigg )\Vert u_4\Vert _{Y_0} \Vert u_5\Vert _{Y_0}. \end{aligned}$$

The ${\mathbf {B}}^j_*$ terms in (6.4) localized to (6.9) can be estimated by the following proposition.

#### Proposition 6.10

Let ${\mathcal {Q}}'(u_1,\ldots ,u_5)$ be such that

$$\begin{aligned}&\big | {{\mathcal {F}}}_{t,x}{\mathcal {Q}}'(u_1, \ldots , u_5) (\tau ,n)\big | \\&\quad \lesssim \sum _{n=n_1+n_2+n_0} \sum _{n_0=n_3+n_4+n_5} {{\,\mathrm{\int }\,}}_{{\mathbb {R}}^5} \frac{\max _j \langle n_j \rangle ^{9\theta }}{\langle n_{\max } \rangle \langle n'_{\max } \rangle \langle \tau -n^3 \rangle ^{1+4\delta }} \prod _{j=1}^5 | \widehat{u}_j(\tau _j, n_j) | \ d\tau _1 \cdots d\tau _5, \end{aligned}$$

where $n_{\max } = \max (|n_1|, |n_2|) \ge |n_0|$ and $n'_{\max } = \max _{j=3,4,5} |n_j|$. Then, the following estimate holds

$$\begin{aligned} \big \Vert {\mathcal {Q}}' (u_1, \ldots , u_5) \Vert _{Z_1}&\lesssim \prod _{j=1}^5 \Vert u_j\Vert _{Y_0}. \end{aligned}$$

#### Proof of Proposition 6.9

Due to the $\theta$ loss in the largest frequency when estimating $\alpha$, we will distinguish two cases: when $|n_1|^{\frac{1}{2}}\lesssim |n_3|$ and when $|n_1|^{\frac{1}{2}}\gg |n_3|$.

**Case 1:** $$|n_1|^{\frac{1}{2}}\lesssim |n_3|$$

Using the notation $\Psi (n, {\overline{n}}_{1\ldots 5}) = n^3 - n_1^3 - \ldots - n^3_5$ and the change of variables $\sigma _j = \tau _j - n_j^3$, $j=1,\ldots ,5$, we start by applying Minkowski’s inequality and Schur’s test to obtain

$$\begin{aligned}&\text {LHS of }(6.11) \\&\lesssim {{\,\mathrm{\int }\,}}_{\sigma _1, \ldots , \sigma _5} \bigg \Vert \sum _{\mu } \frac{1}{\langle \tau -n^3 - {\bar{\sigma }} + \mu \rangle ^{1-\theta }}\bigg ( \sum _{\begin{array}{c} n=n_1+\ldots +n_5,\\ \Psi (n, {\overline{n}}_{1\ldots 5}) = \mu \end{array}} \frac{|n_1| \langle n_1 \rangle ^{3\theta } }{ \max (\langle n_3 \rangle , \langle n \rangle )^\frac{1}{2} } \prod _{j=1}^5 |\widehat{u}_j(\sigma _j+n_j^3, n_j)| \bigg ) \bigg \Vert _{\ell ^p_n L^{q_0}_\tau } \\&\lesssim {{\,\mathrm{\int }\,}}_{\sigma _1, \ldots , \sigma _5} \bigg \Vert \sum _{\begin{array}{c} n=n_1+\ldots +n_5,\\ \Psi (n, {\overline{n}}_{1\ldots 5}) = \mu \end{array}} \frac{|n_1| \langle n_1 \rangle ^{3\theta } }{ \max (\langle n_3 \rangle , \langle n \rangle )^\frac{1}{2}} \prod _{j=1}^5 |\widehat{u}_j(\sigma _j + n_j^3, n_j)| \bigg \Vert _{\ell ^p_n \ell ^{q_1}_\mu }, \end{aligned}$$

where ${\bar{\sigma }} = \sigma _1 + \ldots + \sigma _5$ and $\theta < \frac{\delta }{2}$. Let $f_j(\sigma ,n) = \langle n \rangle ^\frac{1}{2} | \widehat{{\mathbf {P}}_{N_j} u}_j(\sigma + n^3, n)|$, $j=1,2$, and $f_k(\sigma ,n) = |\widehat{{\mathbf {P}}_{N_k} u}_k(\sigma + n^3,n)|$, $k=3,4,5$, with $N_1\sim N_2 \gg N_3 \ge N_4 \ge N_5$ dyadic numbers with $N_1 \lesssim N_3^2$. Omitting the time dependence, using Hölder’s inequality and the standard divisor counting estimate, we have

$$\begin{aligned}&N_1^{3\theta } N_3^{-\frac{1}{2}} \bigg \Vert \sum _{\begin{array}{c} n=n_1+\ldots +n_5,\\ \Psi (n, {\overline{n}}_{1\ldots 5}) = \mu \end{array}} \prod _{j=1}^5 f_j(n_j) \bigg \Vert _{\ell ^p_n \ell ^{q_1}_\mu } \\&\lesssim N_1^{3\theta + \varepsilon } N_3^{-\frac{1}{2}} \bigg \Vert \sum _{(n_4, n_5)} f_4(n_4) f_5(n_5) \bigg (\sum _{\begin{array}{c} n_1 + n_2 + n_3 = n - n_4 - n_5 ,\\ n_1^3 + n_2^3 + n_3^3 = n^3 - n_4^3 - n_5^3 - \mu \end{array}} \prod _{j=1}^3 |f_j(n_j)|^p \bigg )^{\frac{1}{p}} \bigg \Vert _{\ell ^p_n \ell ^{q_1}_\mu } \\&\lesssim N_1^{3\theta + \varepsilon } N_3^{-\frac{1}{2}} \Vert f_1\Vert _{\ell ^p_n} \Vert f_2\Vert _{\ell ^p_n} \Vert f_3\Vert _{\ell ^p_n} \Vert f_4\Vert _{\ell ^1_n} \Vert f_5\Vert _{\ell ^1_n}. \end{aligned}$$

Applying the previous estimate, we obtain

6.12 $$\begin{aligned} \text {LHS of }(6.11)&\lesssim \sum _{N_1, \ldots , N_5} N_1^{3\theta +\varepsilon } N_3^{-\frac{1}{2}} \bigg (\prod _{j=1}^3 \Vert f_j\Vert _{L^1_\sigma \ell ^p_n} \bigg ) \bigg ( \prod _{k=4}^5 \Vert f_k\Vert _{L^1_\sigma \ell ^1_n} \bigg ) \nonumber \\&\lesssim \sum _{N_1, \ldots , N_5} N_1^{3\theta +\varepsilon } N_3^{-1} (N_4 N_5)^{1-\frac{1}{p} - \frac{1}{2} +} \bigg (\prod _{j=1}^3\Vert u_j\Vert _{Z_0} \bigg ) \bigg ( \prod _{k=4}^5 \Vert u_k\Vert _{ Y_0} \bigg ). \end{aligned}$$

Using the fact that $N_1 \sim N_2 \lesssim N_3^2$, for $\varepsilon , \theta <\frac{\delta }{2}$ and $\delta$ small enough, the estimate follows from summing in the dyadic numbers.

**Case 2:** $$|n_1|^{\frac{1}{2}}\gg |n_3|$$

In this case, we need a different approach to control the small power of $N_1$ in the multiplier as well as the $\varepsilon$-loss from using the divisor counting estimate. Note that $\Psi (n, {\overline{n}}_{1\ldots 5}) = 3(n_1+n_2)(n_1 + n_3 + n_4 + n_5)(n_2 + n_3 + n_4 + n_5) + 3(n_3+n_4)(n_3+n_5)(n_4+n_5)$. Since

$$\begin{aligned}&|(n_3+n_4)(n_3+n_5)(n_4+n_5)| \lesssim |n_3|^3 \ll |n_1|^{\frac{3}{2}},\\&|(n_1+n_2)(n_1 + n_3 + n_4 + n_5)(n_2 + n_3 + n_4 + n_5)| \gtrsim |n_1|^2, \end{aligned}$$

then $|\Psi (n, {\overline{n}}_{1\ldots 5})| \gtrsim |n_1|^2$. Following the previous strategy, we have

$$\begin{aligned} \text {LHS of } (6.9)&\lesssim {{\,\mathrm{\int }\,}}_{\sigma _1, \ldots , \sigma _5} \bigg \Vert \sum _{n=n_1+ \ldots +n_5} \frac{|n_1| \langle n_1 \rangle ^{3\theta }}{\max (\langle n \rangle , \langle n_3 \rangle )^\frac{1}{2}} \\&\quad \times \frac{1}{ \langle \tau - n^3 - {\bar{\sigma }} + \Psi (n, {\overline{n}}_{1\ldots 5}) \rangle ^{1-\theta } \langle \tau -n^3 \rangle ^{\delta - \theta }}\prod _{j=1}^5 |\widehat{u}_j(\sigma _j + n_j^3, n_j)| \bigg \Vert _{\ell ^p_n L^{q_0}_\tau } \end{aligned}$$

Thus, in order to control the small powers of $\langle n_1 \rangle$, we use the following fact

$$\begin{aligned} |n_1|^2 \lesssim \langle \Psi (n, {\overline{n}}_{1\ldots 5}) \rangle \lesssim \langle \tau - n^3 - {\bar{\sigma }} + \Psi (n, {\overline{n}}_{1\ldots 5}) \rangle \langle \tau - n^3 \rangle \langle \sigma _1 \rangle \cdots \langle \sigma _5 \rangle . \end{aligned}$$

To gain a power of $\langle \tau -n^3 \rangle$, we impose $\theta \le \frac{\delta }{2}$. For $\langle \tau -n^3 - {\bar{\sigma }}+ \Psi (n, {\overline{n}}_{1\ldots 5}) \rangle$, when applying Schur’s estimate, we want to keep $\tfrac{\delta }{4}$ of this quantity. Thus, with $1 + \frac{1}{q_0} = \frac{1}{q_1} + \frac{1}{r}$, we need

$$\begin{aligned} 1-\theta - \frac{\delta }{4} > \frac{1}{r} = 1 - \frac{\delta }{2} \implies \theta < \frac{\delta }{4}. \end{aligned}$$

We can obtain a power of $\langle \sigma _k \rangle ^{\alpha }$ for $k=4,5$, given that

$$\begin{aligned} \Vert \langle \sigma \rangle ^\alpha f_k\Vert _{\ell ^1_n L^1_\sigma } \lesssim \Vert \langle \sigma \rangle ^{\alpha + \frac{1}{2} - \delta +} f_k \Vert _{\ell ^1_n L^{r_0}_\sigma } \lesssim \Vert \langle \sigma \rangle ^{\frac{1}{2}} f_k \Vert _{\ell ^1_n L^{r_0}_\sigma }, \end{aligned}$$

given that $\alpha + \frac{1}{2} - \delta< \frac{1}{2} \implies \alpha < \delta$, thus we can choose $\alpha = \frac{\delta }{4}$. Similarly, for $\langle \sigma _j \rangle ^\beta$, $j=1,2,3$, we have

$$\begin{aligned} \Vert \langle \sigma \rangle ^\beta f_j \Vert _{L^1_\sigma \ell ^p_n} \lesssim \Vert \langle \sigma \rangle ^{\beta + 1 - 4\delta +} f_j \Vert _{L^{q_0}_\sigma \ell ^p_n} \lesssim \Vert \langle \sigma \rangle ^{1- 2\delta } f_j \Vert _{L^{q_0}_\sigma \ell ^p_n}, \end{aligned}$$

for $\beta = \frac{\delta }{4}$. Combining all of these powers, we get $\langle \Psi (n, {\overline{n}}_{1\ldots 5}) \rangle ^{-\frac{\delta }{4}}\lesssim N_1^{-\frac{\delta }{2}}$ which we use in (6.12) instead of the condition $N_1\lesssim N_3^2$.

$$\square $$

#### Proof of Proposition 6.10

We have the following estimate

$$\begin{aligned}&\Vert {\mathcal {Q}}'(u_1, \ldots , u_5) \Vert _{Z_1} \\&\lesssim \bigg \Vert \sum _{n=n_1+n_2+n_0} \sum _{n_0=n_3+n_4+n_5} {{\,\mathrm{\int }\,}}_{{\mathbb {R}}^5} \frac{\max _j \langle n_j \rangle ^{9\theta } \langle n \rangle ^\frac{1}{2}}{\langle n_{\max } \rangle \langle n'_{\max } \rangle \langle \tau -n^3 \rangle ^{5 \delta }} \prod _{j=1}^5 | \widehat{u}_j(\tau _j, n_j) | \ d\tau _1 \cdots d\tau _5 \bigg \Vert _{\ell ^p_n L^{q_0}_\tau } \\&\lesssim \bigg \Vert \sum _{n=n_1+n_2+n_0} \sum _{n_0=n_3+n_4+n_5} \frac{\max _j \langle n_j \rangle ^{9\theta } \langle n \rangle ^\frac{1}{2}}{\langle n_{\max } \rangle \langle n'_{\max } \rangle } \prod _{j=1}^5 \Vert \widehat{u}_j( n_j) \Vert _{L^1_\tau } \bigg \Vert _{\ell ^p_n }. \end{aligned}$$

Let $f_j(\tau ,n) = \langle n \rangle ^\frac{1}{2} | \widehat{{\mathbf {P}}_{N_j} u} (\tau ,n)|$, for dyadic numbers $N_j$, $j=1, \ldots , 5$. By symmetry, we can assume without loss of generality that $N_1 \ge N_2$, $N_3 \ge N_4 \ge N_5$. We will consider two cases: $|n_1| \ge |n_3|$ or $|n_1| < |n_3|$. Since the strategies follow a similar approach, we will only focus on the former.

Assume that $|n_1| \ge |n_3|$. Using Young’s and Hölder’s inequality, we get

$$\begin{aligned} \Vert {\mathcal {Q}}' (u_1, \ldots , u_5)\Vert _{Z_1}&\lesssim \sum _{N_1, \ldots , N_5} N_1^{9\theta - 1} (N_2 N_4 N_5)^{-\frac{1}{2} } N_3^{-\frac{3}{2}} \bigg \Vert \sum _{n=n_1+ \ldots + n_5} \Vert f_j(n_j)\Vert _{L^1_\tau } \bigg \Vert _{\ell ^p_n} \\&\lesssim \sum _{N_1, \ldots , N_5} N_1^{9\theta - 1} (N_2 N_4 N_5)^{\frac{1}{2} - \frac{1}{p} + } N_3^{-\frac{1}{2} - \frac{1}{p} +} \prod _{j=1}^5 \Vert u_j\Vert _{Y_0}. \end{aligned}$$

It only remains to sum in the dyadics

$$\begin{aligned}&\sum _{N_1, \ldots , N_5} N_1^{9\theta - 1} (N_2 N_4 N_5)^{\frac{1}{2} - \frac{1}{p} + } N_3^{-\frac{1}{2} - \frac{1}{p} +} \\&\quad \lesssim \sum _{N_1, \ldots , N_5} (N_1 N_2)^{-\theta } (N_3N_4N_5)^{\frac{1}{6} - \frac{1}{p} + (-\frac{1}{2} - \frac{1}{p} + 11\theta )/3 +} \end{aligned}$$

and the estimate follows if $3\delta< \theta < \frac{4}{11p}.$

$$\square $$

## Appendix A: Equation for *w*

Here we present the equation for *w* after using second iteration once, following the strategy described in Sect. 4.

$$\begin{aligned} w&= \varphi (t) S(t) u_0 + \varphi _T\cdot \mathcal {IR}(u, u, u) \nonumber \\&\quad + \varphi _T\cdot ({\mathcal {D}}\mathcal {NR}_{C,\ge } + {\mathcal {D}}\mathcal {NR}_{D,\ge })(w,\overline{w},w) + \varphi _T\cdot ({\mathcal {D}}\mathcal {NR}_{C,>} + {\mathcal {D}}\mathcal {NR}_{D,>})(w,w,\overline{w}) \nonumber \\&\quad + \varphi _T \big ( {\mathbf {B}}^{ 0}_{A,\ge } (w, \overline{u},u) + {\mathbf {B}}^{ 1}_{A,\ge }(w, \overline{u},u) + {\mathbf {B}}^{ 2}_{A,\ge } (w, \overline{w},u) + {\mathbf {B}}^{ 3}_{A,\ge }(w, \overline{u},w) \big ) \nonumber \\&\quad + \varphi _T \big ( {\mathbf {B}}^{ 0}_{A,>} (w, u,\overline{u}) + {\mathbf {B}}^{ 1}_{A,>}(w, u,\overline{u}) + {\mathbf {B}}^{ 2}_{A,>} (w, w,\overline{u}) + {\mathbf {B}}^{ 3}_{A,>}(w, u,\overline{w}) \big ) \nonumber \\&\quad + \varphi _T \big ( {\mathbf {B}}^{ 0}_{B,\ge } (w, \overline{w},u) + {\mathbf {B}}^{ 1}_{B,\ge } (w, \overline{w},u) + {\mathbf {B}}^{ 2}_{B, \ge } (w, \overline{w},u) + {\mathbf {B}}^{ 3}_{B, \ge } (w, \overline{w},w) \big ) \nonumber \\&\quad + \varphi _T \big ( {\mathbf {B}}^{ 0}_{B,>} (w, w,\overline{u}) + {\mathbf {B}}^{ 1}_{B,>} (w, w,\overline{u}) + {\mathbf {B}}^{ 2}_{B,>} (w, w,\overline{u}) + {\mathbf {B}}^{ 3}_{B,>} (w, w,\overline{w}) \big ) \nonumber \\&\quad + \varphi _T({\mathcal {D}}\mathcal {NR}_{A, \ge } + {\mathcal {D}}\mathcal {NR}_{B, \ge }+ {\mathcal {D}}\mathcal {NR}_{C, \ge } + {\mathcal {D}}\mathcal {NR}_{D, \ge })\big (\varphi _T\cdot {\mathbf {G}}_{A, \ge }[w,\overline{u}, u],\overline{u}, u\big ) \nonumber \\&\quad + \varphi _T({\mathcal {D}}\mathcal {NR}_{A, \ge } + {\mathcal {D}}\mathcal {NR}_{B, \ge }+ {\mathcal {D}}\mathcal {NR}_{C, \ge } + {\mathcal {D}}\mathcal {NR}_{D, \ge })\big (\varphi _T\cdot {\mathbf {G}}_{A,>}[w,u,\overline{u}],\overline{u}, u\big ) \nonumber \\&\quad + \varphi _T({\mathcal {D}}\mathcal {NR}_{A, \ge } + {\mathcal {D}}\mathcal {NR}_{B, \ge }+ {\mathcal {D}}\mathcal {NR}_{C, \ge } + {\mathcal {D}}\mathcal {NR}_{D, \ge })\big (\varphi _T\cdot {\mathbf {G}}_{B, \ge }[w,\overline{w},u],\overline{u}, u\big ) \nonumber \\&\quad + \varphi _T({\mathcal {D}}\mathcal {NR}_{A, \ge } + {\mathcal {D}}\mathcal {NR}_{B, \ge }+ {\mathcal {D}}\mathcal {NR}_{C, \ge } + {\mathcal {D}}\mathcal {NR}_{D, \ge })\big (\varphi _T\cdot {\mathbf {G}}_{B,>}[w,w,\overline{u}],\overline{u}, u\big ) \nonumber \\&\quad + \varphi _T({\mathcal {D}}\mathcal {NR}_{A,>} + {\mathcal {D}}\mathcal {NR}_{B,>}+ {\mathcal {D}}\mathcal {NR}_{C,>} + {\mathcal {D}}\mathcal {NR}_{D,>})\big (\varphi _T\cdot {\mathbf {G}}_{A, \ge }[w,\overline{u}, u],u,\overline{u}\big ) \nonumber \\&\quad + \varphi _T({\mathcal {D}}\mathcal {NR}_{A,>} + {\mathcal {D}}\mathcal {NR}_{B,>}+ {\mathcal {D}}\mathcal {NR}_{C,>} + {\mathcal {D}}\mathcal {NR}_{D,>})\big (\varphi _T\cdot {\mathbf {G}}_{A,>}[w,u,\overline{u}],u,\overline{u}\big ) \nonumber \\&\quad + \varphi _T({\mathcal {D}}\mathcal {NR}_{A,>} + {\mathcal {D}}\mathcal {NR}_{B,>}+ {\mathcal {D}}\mathcal {NR}_{C,>} + {\mathcal {D}}\mathcal {NR}_{D,>})\big (\varphi _T\cdot {\mathbf {G}}_{B, \ge }[w,\overline{w},u],u,\overline{u}\big ) \nonumber \\&\quad + \varphi _T({\mathcal {D}}\mathcal {NR}_{A,>} + {\mathcal {D}}\mathcal {NR}_{B,>}+ {\mathcal {D}}\mathcal {NR}_{C,>} + {\mathcal {D}}\mathcal {NR}_{D,>})\big (\varphi _T\cdot {\mathbf {G}}_{B, >}[w,w,\overline{u}],u,\overline{u}\big ) \nonumber \\&\quad + \varphi _T({\mathcal {D}}\mathcal {NR}_{B, \ge }+{\mathcal {D}}\mathcal {NR}_{C, \ge } + {\mathcal {D}}\mathcal {NR}_{D, \ge })\big (w, \overline{\varphi _T\cdot {\mathbf {G}}_{A, \ge }[w,\overline{u}, u] }, u\big ) \nonumber \end{aligned}$$

$$\begin{aligned}&\quad + \varphi _T({\mathcal {D}}\mathcal {NR}_{B, \ge }+{\mathcal {D}}\mathcal {NR}_{C, \ge } + {\mathcal {D}}\mathcal {NR}_{D, \ge })\big (w, \overline{\varphi _T\cdot {\mathbf {G}}_{A,>}[w,u, \overline{u}] }, u\big ) \nonumber \\&\quad + \varphi _T({\mathcal {D}}\mathcal {NR}_{B, \ge }+{\mathcal {D}}\mathcal {NR}_{C, \ge } + {\mathcal {D}}\mathcal {NR}_{D, \ge })\big (w, \overline{\varphi _T\cdot {\mathbf {G}}_{B, \ge }[w,\overline{w},u] }, u\big ) \nonumber \\&\quad + \varphi _T({\mathcal {D}}\mathcal {NR}_{B, \ge }+{\mathcal {D}}\mathcal {NR}_{C, \ge } + {\mathcal {D}}\mathcal {NR}_{D, \ge })\big (w, \overline{\varphi _T\cdot {\mathbf {G}}_{B,>}[w,w, \overline{u}] }, u\big ) \nonumber \\&\quad + \varphi _T({\mathcal {D}}\mathcal {NR}_{B,>}+{\mathcal {D}}\mathcal {NR}_{C,>} + {\mathcal {D}}\mathcal {NR}_{D,>})\big (w, \varphi _T\cdot {\mathbf {G}}_{A, \ge }[w,\overline{u}, u] , \overline{u}\big ) \nonumber \\&\quad + \varphi _T({\mathcal {D}}\mathcal {NR}_{B,>}+{\mathcal {D}}\mathcal {NR}_{C,>} + {\mathcal {D}}\mathcal {NR}_{D,>})\big (w, \varphi _T\cdot {\mathbf {G}}_{A,>}[w,u, \overline{u}] , \overline{u}\big ) \nonumber \\&\quad + \varphi _T({\mathcal {D}}\mathcal {NR}_{B,>}+{\mathcal {D}}\mathcal {NR}_{C,>} + {\mathcal {D}}\mathcal {NR}_{D,>})\big (w, \varphi _T\cdot {\mathbf {G}}_{B, \ge }[w,\overline{w},u] , \overline{u}\big ) \nonumber \\&\quad + \varphi _T({\mathcal {D}}\mathcal {NR}_{B,>}+{\mathcal {D}}\mathcal {NR}_{C,>} + {\mathcal {D}}\mathcal {NR}_{D,>})\big (w, \varphi _T\cdot {\mathbf {G}}_{B,>}[w,w, \overline{u}] , \overline{u}\big ) \nonumber \\&\quad + \varphi _T({\mathcal {D}}\mathcal {NR}_{C,\ge } + {\mathcal {D}}\mathcal {NR}_{D, \ge })\big (w, \overline{w},\varphi _T\cdot {\mathbf {G}}_{A, \ge }[w,\overline{u}, u]\big ) \nonumber \\&\quad + \varphi _T({\mathcal {D}}\mathcal {NR}_{C,\ge } + {\mathcal {D}}\mathcal {NR}_{D, \ge })\big (w, \overline{w},\varphi _T\cdot {\mathbf {G}}_{A,>}[w,u,\overline{u}]\big ) \nonumber \\&\quad + \varphi _T({\mathcal {D}}\mathcal {NR}_{C,\ge } + {\mathcal {D}}\mathcal {NR}_{D, \ge })\big (w, \overline{w},\varphi _T\cdot {\mathbf {G}}_{B, \ge }[w,\overline{w}, u]\big ) \nonumber \\&\quad + \varphi _T({\mathcal {D}}\mathcal {NR}_{C,\ge } + {\mathcal {D}}\mathcal {NR}_{D, \ge })\big (w, \overline{w},\varphi _T\cdot {\mathbf {G}}_{B,>}[w,w,\overline{u}]\big ) \nonumber \\&\quad + \varphi _T({\mathcal {D}}\mathcal {NR}_{C,>} + {\mathcal {D}}\mathcal {NR}_{D,>})\big (w,w, \overline{\varphi _T\cdot {\mathbf {G}}_{A, \ge }[w,\overline{u}, u]} \big ) \nonumber \\&\quad + \varphi _T({\mathcal {D}}\mathcal {NR}_{C,>} + {\mathcal {D}}\mathcal {NR}_{D,>})\big (w, w,\overline{\varphi _T\cdot {\mathbf {G}}_{A,>}[w,u,\overline{u}] }\big ) \nonumber \\&\quad + \varphi _T({\mathcal {D}}\mathcal {NR}_{C,>} + {\mathcal {D}}\mathcal {NR}_{D,>})\big (w, w, \overline{\varphi _T\cdot {\mathbf {G}}_{B, \ge }[w,\overline{w}, u]} \big ) \nonumber \\&\quad + \varphi _T({\mathcal {D}}\mathcal {NR}_{C,>} + {\mathcal {D}}\mathcal {NR}_{D,>})\big (w, w, \overline{\varphi _T\cdot {\mathbf {G}}_{B,>}[w,w,\overline{u}] }\big ) \nonumber \\&\quad + \varphi _T \Big ( {\mathbf {B}}^{ 2}_{A, \ge } \big (w, \overline{\varphi _T \cdot {\mathbf {G}}_{A, \ge }[w,\overline{u}, u]}, u \big ) + {\mathbf {B}}^{ 2}_{A, \ge } \big (w, \overline{\varphi _T \cdot {\mathbf {G}}_{A, >}[w,u,\overline{u}]}, u \big ) \Big )\qquad \qquad \end{aligned}$$

A.1 $$\begin{aligned}&\quad +\varphi _T \Big ( {\mathbf {B}}^{ 2}_{A, \ge } \big (w, \overline{\varphi _T \cdot {\mathbf {G}}_{B, \ge }[w,\overline{w}, u]}, u \big ) + {\mathbf {B}}^{ 2}_{A, \ge } \big (w, \overline{\varphi _T \cdot {\mathbf {G}}_{B,>}[w,w,\overline{u}]}, u \big ) \Big ) \nonumber \\&\quad + \varphi _T \Big ( {\mathbf {B}}^{ 2}_{A,>} \big (w, \varphi _T \cdot {\mathbf {G}}_{A, \ge }[w,\overline{u}, u], \overline{ u} \big ) + {\mathbf {B}}^{ 2}_{A,>} \big (w, \varphi _T \cdot {\mathbf {G}}_{A,>}[w,u,\overline{u}], \overline{u} \big ) \Big ) \nonumber \\&\quad +\varphi _T \Big ( {\mathbf {B}}^{ 2}_{A,>} \big (w, \varphi _T \cdot {\mathbf {G}}_{B, \ge }[w,\overline{w}, u], \overline{u} \big ) + {\mathbf {B}}^{ 2}_{A,>} \big (w, \varphi _T \cdot {\mathbf {G}}_{B,>}[w,w,\overline{u}], \overline{u} \big ) \Big ) \nonumber \\&\quad + \varphi _T \Big ( {\mathbf {B}}^{ 3}_{A, \ge } \big (w, \overline{u}, \varphi _T\cdot {\mathbf {G}}_{A, \ge }[w, \overline{u}, u] \big ) + {\mathbf {B}}^{ 3}_{A, \ge } \big (w, \overline{u}, \varphi _T\cdot {\mathbf {G}}_{A,>}[w,u, \overline{u}] \big ) \Big ) \nonumber \\&\quad + \varphi _T \Big ( {\mathbf {B}}^{ 3}_{A, \ge } \big (w, \overline{u}, \varphi _T\cdot {\mathbf {G}}_{B, \ge }[w,\overline{w}, u] \big ) + {\mathbf {B}}^{ 3}_{A, \ge } \big (w, \overline{u}, \varphi _T\cdot {\mathbf {G}}_{B,>}[w,w, \overline{u}] \big ) \Big ) \nonumber \\&\quad + \varphi _T \Big ( {\mathbf {B}}^{ 3}_{A,>} \big (w, u, \overline{\varphi _T\cdot {\mathbf {G}}_{A, \ge }[w, \overline{u}, u]} \big ) + {\mathbf {B}}^{ 3}_{A,>} \big (w, u, \overline{\varphi _T\cdot {\mathbf {G}}_{A,>}[w,u, \overline{u}]} \big ) \Big ) \nonumber \\&\quad + \varphi _T \Big ( {\mathbf {B}}^{ 3}_{A,>} \big (w, u, \overline{\varphi _T\cdot {\mathbf {G}}_{B, \ge }[w,\overline{w}, u]} \big ) + {\mathbf {B}}^{ 3}_{A,>} \big (w, u, \overline{\varphi _T\cdot {\mathbf {G}}_{B,>}[w,w, \overline{u}]} \big ) \Big ) \nonumber \\&\quad + \varphi _T\Big ( {\mathbf {B}}^{ 3}_{B, \ge } \big (w,\overline{w}, \varphi _T\cdot {\mathbf {G}}_{A, \ge }[w, \overline{u}, u] \big ) + {\mathbf {B}}^{ 3}_{B, \ge } \big (w,\overline{w}, \varphi _T\cdot {\mathbf {G}}_{A,>}[w, u, \overline{u}] \big ) \Big ) \nonumber \\&\quad +\varphi _T \Big ({\mathbf {B}}^{ 3}_{B, \ge } \big (w,\overline{w}, \varphi _T\cdot {\mathbf {G}}_{B, \ge }[w, \overline{w}, u] \big ) + {\mathbf {B}}^{ 3}_{B, \ge } \big (w,\overline{w}, \varphi _T\cdot {\mathbf {G}}_{B,>}[w, w,\overline{u}] \big ) \Big ) \nonumber \\&\quad + \varphi _T\Big ( {\mathbf {B}}^{ 3}_{B,>} \big (w,w,\overline{ \varphi _T\cdot {\mathbf {G}}_{A, \ge }[w, \overline{u}, u] } \big ) + {\mathbf {B}}^{ 3}_{B,>} \big (w,w, \overline{ \varphi _T\cdot {\mathbf {G}}_{A,>}[w, u, \overline{u}]} \big ) \Big )\nonumber \\&\quad +\varphi _T \Big ({\mathbf {B}}^{ 3}_{B,>} \big (w,w,\overline{ \varphi _T\cdot {\mathbf {G}}_{B, \ge }[w, \overline{w}, u]} \big ) + {\mathbf {B}}^{ 3}_{B,>} \big (w,w,\overline{ \varphi _T\cdot {\mathbf {G}}_{B, >}[w, w,\overline{u}]} \big ) \Big ) . \end{aligned}$$

## Footnotes

1 In the following, we choose to take the ‘$+$’ sign in front of the nonlinearity in (1.8) for simplicity, as the sign will not play a role in the analysis.

2 **Schur’s test:** Let *X*, *Y* be measurable spaces, $K:X\times Y \rightarrow {\mathbb {R}}$ a non-negative Schwartz kernel and $1\le p,q,r\le \infty$ such that $1 + \frac{1}{q} = \frac{1}{p} + \frac{1}{r}$. If for some $C>0$ we have

$$\begin{aligned} \sup _{x\in X}{{\,\mathrm{\int }\,}}_Y |K(x,y)|^r dy + \sup _{y\in Y} {{\,\mathrm{\int }\,}}_X |K(x,y)|^r dx \le C^r \quad \text {then} \quad \bigg \Vert {{\,\mathrm{\int }\,}}_Y K(x,y) f(y) \ dy \bigg \Vert _{L^q(X)} \le C \Vert f\Vert _{L^p(Y)}. \end{aligned}$$
